# Supplementary material for: Of pathogens and party lines: Social conservatism positively associates with COVID-19 precautions among U.S. Democrats but not Republicans
Source: PLoS One. 2021 Jun 29;16(6):e0253326. doi: 10.1371/journal.pone.0253326 (PMC8241032; doi:10.1371/journal.pone.0253326)
Supplement: S1 Appendix — (PDF) [file pone.0253326.s001.pdf]

S1 APPENDIX – SUPPLEMENTARY PROCEDURE TO: Social Conservatism Positively Associates with COVID-19 Precaution among Democrats but not Republicans

Contents

Studies 1 and 2 composite scales 3

Pilot Study scales 11

Analysis software 15

Differences between pre-registered measures, and final measures 16

Software version and source information 19

References 24

Studies 1 and 2 composite scales

Full survey items can be found in the open archives.

Political orientation composites: Modification of Dodd et al.'s (2012) version of Wilson and Patterson's (1968) issues index.

Please indicate whether you agree or disagree, or are uncertain, with regard to each topic listed below. [agree, disagree, uncertain]

<u>Social conservatism composite:</u>

1. Women's equality (r)
2. Charter schools
3. Prayer in public schools
4. Pornography (r)
5. Death penalty
6. Premarital sex (r)
7. Gay marriage (r)
8. Abortion rights (r)
9. Theory of evolution (r)
10. Biblical truth

<u>Economic conservatism composite:</u>

1. Welfare spending (r)
2. Tax cuts
3. Pollution control (r)
4. Aid to foreign countries (r)
5. Socialism (r)
6. Globalization (r)
7. Small government

<u>Militaristic conservatism composite:</u>

1. Bomb cities controlled by terrorists
2. Gun control (r)

3. Military spending
4. Warrantless searches
5. Obedience to authorities
6. Compromise/negotiation with enemies (r)
7. Use nuclear weapons against threats to the US
8. Drone attacks on terror suspects
9. Illegal immigration
10. Waterboarding terror suspects
11. Patriotism
12. Pacifism (r)

Right-wing authoritarianism composites: Aggression-Submission-Conventionalism authoritarianism scale (Dunwoody & Funke, 2016).

The following questions concern values that people may or may not hold. Please select a number to indicate the degree to which you agree or disagree with each statement. [1 – strongly disagree … 7 – strongly agree]

<u>Traditionalism:</u>

1. People emphasize tradition too much. (r)
2. It would be better for society if more people followed social norms.
3. People should respect social norms.
4. Traditions are the foundation of a healthy society and should be respected.
5. Traditions interfere with progress. (r)
6. People should challenge social traditions in order to advance society. (r)

<u>Authoritarian aggression:</u>

1. People should avoid using violence against others even when ordered to do so by the proper authorities. (r)
2. Using force against people is wrong even if done so by those in authority. (r)
3. Strong punishments are necessary in order to send a message.
4. Strong force is necessary against threatening groups
5. It is necessary to use force against people who are a threat to authority.

6. Police should avoid using violence against suspects. (r)

<u>Submission to authority:</u>

1. Our leaders know what is best for us.
2. People should be critical of statements made by those in positions of authority. (r)
3. People should be skeptical of all statements made by those in positions of authority. (r)
4. We should believe what our leaders tell us.
5. People in positions of authority generally tell the truth.
6. Questioning the motives of those in power is healthy for society. (r)

Social dominance orientation composite: Four-item Short Social Dominance Orientation Scale (Pratto et al., 2013).

There are many types of groups in the world: men and women, ethnic and religious groups, nationalities, political factions, etc. Please select a number to rate the degree to which you oppose or favor each statement about groups, where higher numbers mean you favor the statement more, and lower numbers mean you oppose the statement more. [1 – extremely oppose … 7 – extremely favor]

1. In setting priorities, we must consider all groups. (r)
2. We should *not* push for group equality.
3. Group equality should be our ideal. (r)
4. Superior groups should dominate inferior groups.

Pathogen disgust sensitivity composite: Pathogen subscale of the Three-Domain Disgust Scale (Tybur et al., 2009).

Please rate how disgusting you find the concepts described in the items by selecting a number, where lower values mean that you find the concept less disgusting, and higher values mean that you find the concept more disgusting. [1 – not at all disgusting … 7 – extremely disgusting]

1. Stepping on dog poop

2. Sitting next to someone who has red sores on their arm
3. Shaking hands with a stranger who has sweaty palms
4. Seeing some mold on old leftovers in your refrigerator
5. Standing close to a person who has body odor
6. Seeing a cockroach run across the floor
7. Accidentally touching a person's bloody cut

COVID-19 precautionary health behaviors composite:

On average, how often do you currently… [1 – Never … 7 – As often as possible]

1. Wash your hands each day?
2. Use hand sanitizer each day?
3. Disinfect surfaces in your house, like doorknobs or counters, each day?
4. Take supplements to boost your immune system?

Within the last 10 weeks, it has been important to me that (I / my household) make an effort to stock up on... [1 – Not at all important … 7 – Extremely important]

1. Cleaning supplies (such as bleach, disinfectant spray, disinfectant wipes, etc.)
2. Hand sanitizer/hand soap
3. Masks and gloves

When you leave your home, how often do you do each of the following? [1 – Never… 7 – Always]

1. Wear a mask
2. Wear gloves
3. Stay farther than 6 feet away from people

To what extent are you following your local and state lockdown restrictions? [1 – Never follow… 7 – Always follow]

To what degree were you careful in the last week to keep your distance from people outside your household? [1 – Not careful at all… 7 – As careful as possible]

Trust composites:

How much do you think the following sources of information provide advice based on accurate information about what to do during the COVID-19 outbreak? Please select a number, where higher numbers mean you think the source is more accurate, and lower numbers mean you think the source is less accurate [1 – Not accurate at all … 7 – Extremely Accurate (I don't know = NA)]

Trust in liberals and moderates composite:

1. Liberal journalists
2. Centrist journalists
3. MSNBC
4. CNN
5. The New York Times
6. USA Today
7. The Young Turks
8. Chris Hayes
9. Rachel Maddow
10. Lawrence O'Donnell

Trust in conservatives composite:

1. Donald Trump
2. Fox News
3. Breitbart
4. Sean Hannity
5. Tucker Carlson
6. Rush Limbaugh
7. Conservative journalists

Trust in scientists composite:

1. Dr. Anthony Fauci
2. Centers for Disease Control and Prevention (CDC)
3. Health care providers
4. Medical scientists

COVID-19 domain-specific threat-assessments composite: Higher scores indicate finding the direct health consequences of the pandemic less serious, particularly in contrast to other COVID-19-related harms such as threats to personal liberties and the economy.

During the current COVID-19 outbreak, how focused are you on doing the following? [1 – Not focused at all … 7 – As focused as possible]

1. Speaking out to defend personal liberties

How concerned are you about… [1 – Not at all concerned … 7 – Extremely concerned]

1. Personally getting COVID-19 (r)
2. A family member getting COVID-19 (r)
3. Transmitting COVID-19 to a family member (r)
4. Transmitting COVID-19 to a stranger (r)
5. Transmitting COVID-19 to someone I live with (r)
6. The economic effects of the COVID 19 outbreak
7. Losing personal liberties because of COVID-19 lockdown orders

Within the last 10 weeks, it has been important to me that (I / my household) make an effort to stock up on guns and ammunition [1 – Not at all important… 7 – Extremely important]

How severe would the consequences of catching COVID-19 be to… [1 – Extremely small consequences … 7 – Extremely large consequences]

1. Your own health (r)
2. The health of your family members and loved ones (r)
3. The health of people in your local community (r)

How much more concerned would you be about COVID-19 if you DID NOT engage in protective behaviors? (r) [1 – No difference in concern … 7 – Intensely more concerned]

Please indicate how strongly you agree or disagree with each of the following statements [1 – Strongly disagree … 7 – Strongly agree]

1. I think that the risk of the COVID-19 outbreak has been overstated in the media.
2. I think that the worst of the COVID-19 outbreak has already occurred.
3. I am confident that this country will have COVID-19 under control by October 1st.
4. I think that the economic costs of the COVID-19 response outweigh the public health benefits.
5. I think that the threat of COVID-19 is overblown.
6. The costs to personal liberty as a result of the response to the COVID-19 outbreak outweigh the public health benefits

COVID-19 economic precautions composite:

During the current COVID-19 outbreak, how focused are you on doing the following? [1 – Not focused at all … 7 – As focused as possible]

1. Saving money
2. Reducing discretionary spending
3. Delaying major financial decisions
4. Preparing for a major economic downturn

Within the last 10 weeks, it has been important to me that (I / my household) make an effort to stock up on cash [1 – Not at all important… 7 – Extremely important]

News consumption composites:

On a scale from 1 to 7, how often do you watch, listen to, or read news from the following outlets? [1 – Never … 7 – Very frequently]

<u>Liberal news media consumption composite:</u>

1. CNN
2. The New York Times
3. The Washington Post
4. Slate
5. The Atlantic Monthly

6. The New Yorker
7. NBC news
8. Politico
9. Buzzfeed News
10. MSNBC

Conservative news media consumption composite:

1. Fox News
2. Breitbart
3. Infowars
4. The Federalist
5. The Daily Caller
6. National Review
7. Washington Examiner
8. RedState

Centrist news media consumption composite:

1. The Wall Street Journal
2. USA Today
3. FiveThirtyEight
4. NPR

Endorsement of public health interventions composite:

Please indicate how strongly you agree or disagree with each of the following statements [1 – Strongly disagree … 7 – Strongly agree]

1. The government should be able to set public health policies that restrict tobacco use (r)
2. Others' tobacco use poses a danger to my personal health (r)
3. Unrestricted tobacco use is a matter of personal liberty

Political affiliation closeness composite:

Please indicate how strongly you agree or disagree with each of the following statements [1 – Strongly disagree … 7 – Strongly agree]

1. I identify with other members of [relevant political affiliation inserted here]
2. I am like other members of [relevant political affiliation inserted here]
3. [Relevant political affiliation inserted here] is an important reflection of who I am

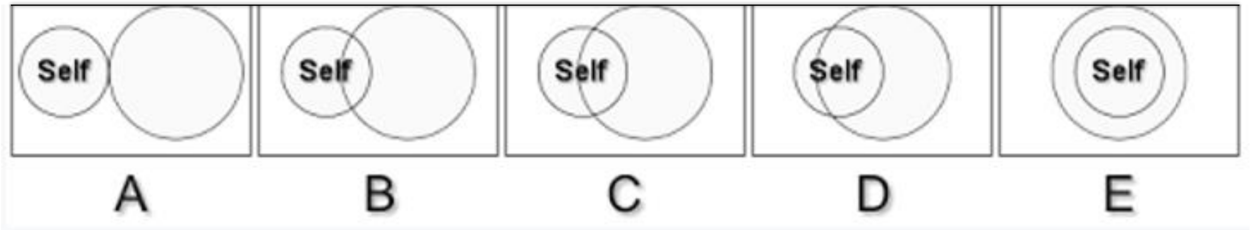

Which picture best represents the way you perceive your relationship with the [relevant political affiliation inserted here]? [A … E]

Pilot Study scales

COVID-19 precautionary health behaviors composite, Pilot Study version:

On average, how often do you currently… [1 – Never … 7 – As often as possible]

1. Wash your hands each day?
2. Use hand sanitizer each day?
3. Disinfect surfaces in your house, like doorknobs or counters, each day?

Within the last 10 weeks, it has been important to me that (I / my household) make an effort to stock up on... [1 – Not at all important … 7 – Extremely important]

1. Cleaning supplies (such as bleach, disinfectant spray, disinfectant wipes, etc.)
2. Hand sanitizer
3. Hand soap

When you leave your home, how often do you do each of the following? [1 – Never… 7 – Always]

1. Wear a mask
2. Wear gloves
3. Stay farther than 6 feet away from people

To what extent are you following your local and state lockdown restrictions? [1 – Never follow … 7 – Always follow (not applicable = NA)]

COVID-19 domain-specific threat-assessments composite, Pilot Study version: Higher scores indicate finding the direct health consequences of the pandemic less serious, particularly in contrast to other COVID-19-related harms such as threats to the economy.

How concerned are you about… [1 – Not at all concerned … 7 – Extremely concerned]

1. Personally getting COVID-19 (r)
2. Transmitting COVID-19 to someone else (r)

How much of a risk do you think COVID-19 directly poses to… [1 – Extremely small risk … 7 – Extremely large risk]

1. Your health (r)
2. The health of your family members and loved ones (r)

How worried are you about the future financial repercussions of the COVID-19 outbreak? [1 – Not worried at all … 7 – Extremely worried]

Please indicate how strongly you agree or disagree with each of the following statements [1 – Strongly disagree … 7 – Strongly agree]

1. I think that the risk of the COVID-19 outbreak has been overstated in the media.
2. I think that the worst of the COVID-19 outbreak has already occurred.
3. I am confident that this country will have COVID-19 under control by September 1st.
4. I think that the economic costs of the COVID-19 response outweigh the public health benefits.
5. I think that the threat of COVID-19 is overblown.

6. I think that many of my family and friends could get coronavirus, but by and large we'll be fine.

Pathogen disgust sensitivity images composite: Modified version of Curtis et al.'s (2004) visual disgust sensitivity measure. Note that images containing potentially identifying information have been removed. See Curtis and colleagues (2004) for full measure.

Please rate how you feel toward each of the following images. [1 – Not at all disgusting … 7 – Extremely disgusting]

1. 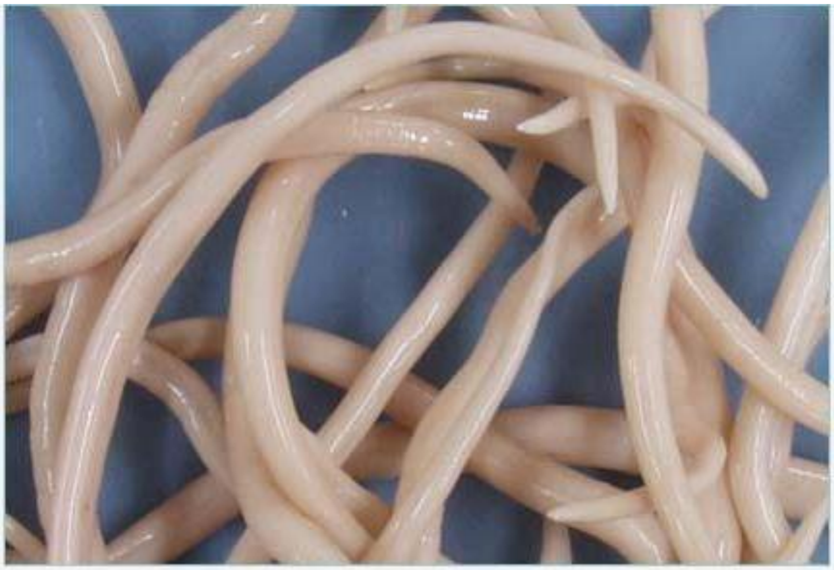
2. 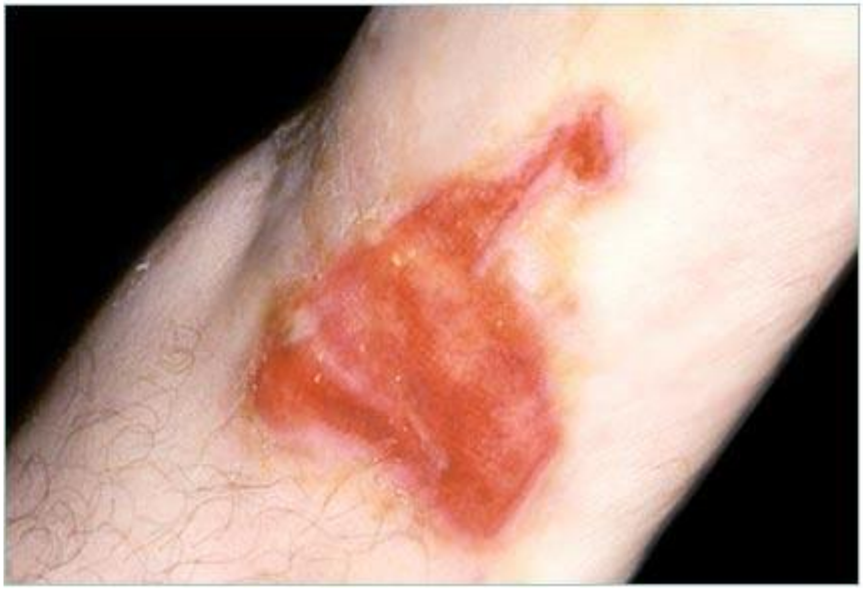
3. redacted

4. 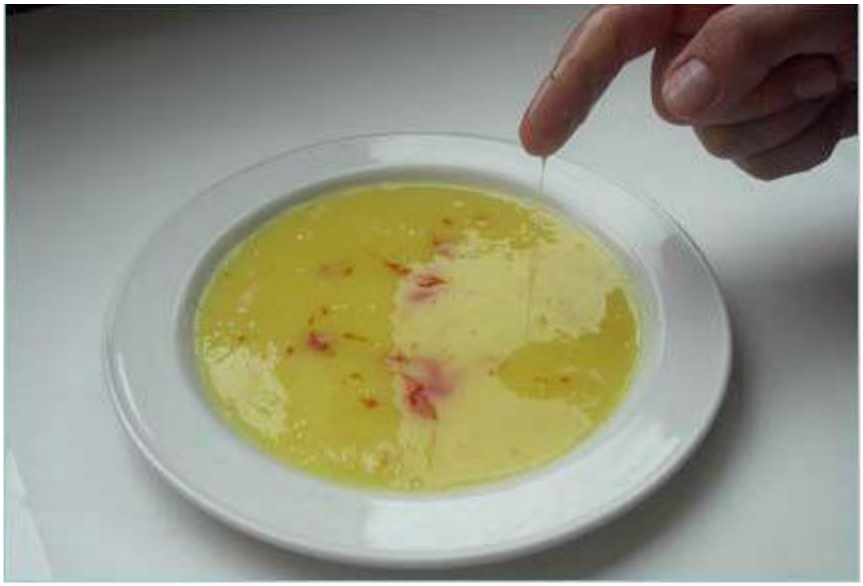

5. 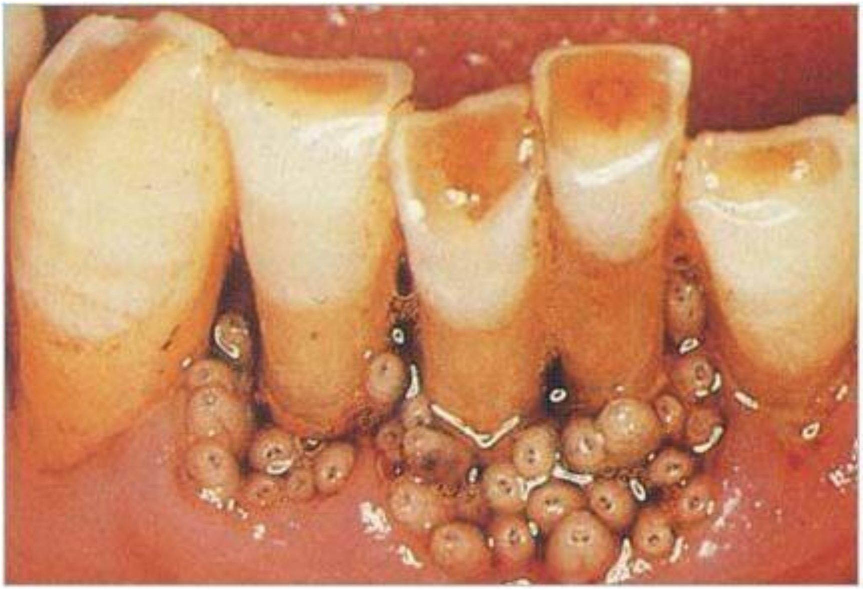

6. 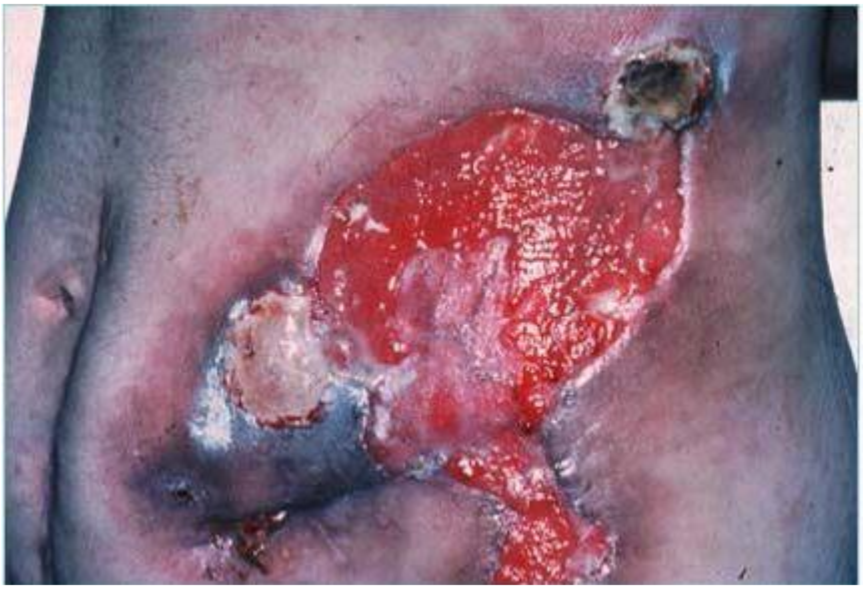
7. redacted
8. 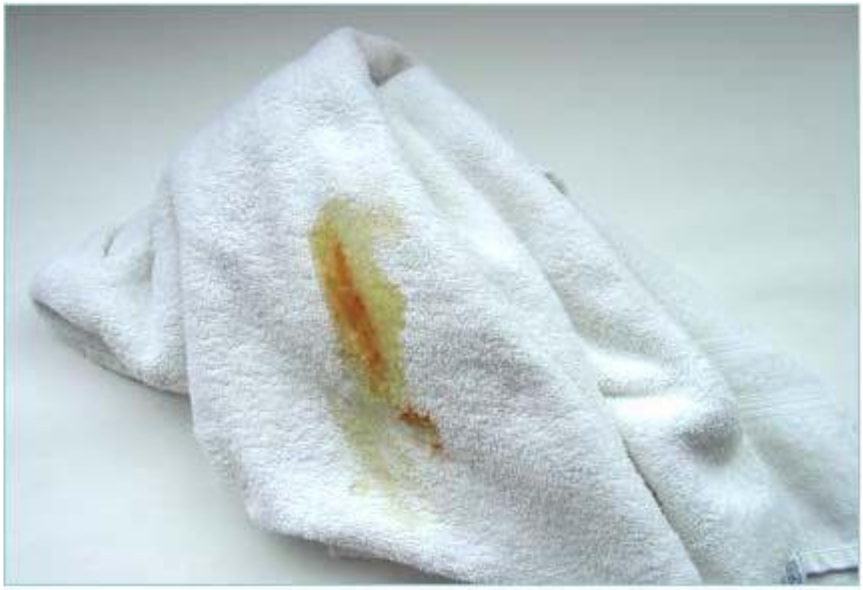

Analysis software

We used R (R Core Team, 2020) and the R-packages *car* (Fox & Weisberg, 2019), *corrplot* (Wei & Simko, 2017), *cowplot* (Wilke, 2019), *dplyr* (Wickham et al., 2020) , *ggpmisc (*Aphalo, 2020)*, ggplot2* (Wickham, 2016), *ggpubr* (Kassambara, 2020), *GPArotation* (Bernaards & Jennrich, 2005), *gridExtra* (Auguie, 2017), *interactions* (Long, 2019), *likert* (Bryer &

Speerschneider, 2016), *lm.beta* (Behrendt, 2014), *MASS* (Venables & Ripley, 2002), *papaja* (Aust & Barth, 2020), *parameters (*Lüdecke, Ben-Shachar & Makowski, 2020*)*, *psych* (Revelle, 2019), *purr* (Henry & Wickham, 2020), *readxl* (Wickham & Bryan, 2019), *sjPlot* (Lüdecke, 2020), *stringr* (Wickham, 2019), *tibble* (Müller & Wickham, 2020), *tidyr* (Wickham & Henry, 2020), *wesanderson* (Ram & Wickham, 2018), and *yarrr* (Phillips, 2017) for our analyses.

The code that produced all analyses in the main text and supplement is openly available.

Differences between pre-registration, and final manuscript

There are several differences between the pre-registered measures and those reported in the main text and supplement. Here, we explain those differences.

- COVID-19 precautionary behaviors composite measure: We originally asked participants about precautionary behaviors that ultimately did not make it into the final composite measure used in analyses here. More specifically, participants were asked how much of an effort they had made to acquire supplies such as toilet paper and canned foods. However, the precautionary behaviors composite is designed to measure health precautions specifically, and not non-health related behaviors that may result from more downstream consequences of the COVID-19 outbreak (such as shortages of food and toilet paper in stores early in the pandemic). Therefore, upon reflection, we realized that it would be erroneous to include those items in a measure of health/pathogen avoidance precautions, and those items were excluded from analysis.
- Moral disgust: In the original survey, in addition to asking participants about pathogen disgust sensitivity using the pathogen subscale of the Three-Domain Disgust Scale (Tybur et al., 2009), participants were also asked about their moral disgust sensitivity

using the moral subscale of that same scale. We reserve the analyses of this measure for a separate project, and thus do not include them here.

- Disease threat sensitivity: In the original survey, we asked participants about the perceived threat posed by non-COVID-19 diseases, such as Lyme disease and Ebola, in order to measure pathogen avoidance across a broader range of threats, which could then be compared to pathogen avoidance for COVID-19 in particular. We reserve the analyses of this measure for a separate project, and thus do not include them here.
- News media consumption composites: In the main text, we describe the process of creating two partisan news consumption composites (liberal-leaning, and conservative-leaning; the moderate-leaning composite was dropped due to a lack of reliability), based on the Allsides Media Bias ratings (*AllSides Media Bias Ratings*, 2020), a third-party organization that uses a variety of methods to rank the partisan leanings of different news sources. However, several of the news sources that we had originally thought might be useful turned out not to have been rated by Allsides, hence we did not include them in any of the composites. Those news sources were as follows: The Rush Limbaugh Show, STAT News, and the Young Turks. Additionally, participants were asked how often they consumed local broadcast news, and news from social media. Because those sources cannot be placed in any particular partisan-leaning composite, they were also excluded.
- COVID-19 domain-specific threat assessment composite: This composite was designed to measure the extent to which participants thought that the direct health threats of the COVID-19 outbreak were serious, especially in relation to the economic and personal liberty threats that may be downstream consequences of the pandemic. This scale originally included a question that stated, “If we do as we’re told by the authorities about

COVID-19, everything will be fine". However, upon further reflection, we realized that this item did not capture opinions about any of the specific threat domains. Further, we also asked participants how concerned they were about what the COVID-19 outbreak would do to the country. However, we realized that this item was underspecified, as it is impossible to determine what aspects of the pandemic participants were attending to when answering the question. Therefore, these variables were excluded from the final composite measure used for analysis.

- Engagement in non-socially distant behaviors: In the Studies 1 and 2 surveys, as an additional measure of COVID-19-related behavior, a set of five items were originally included that asked participants how often in the last week (from 0 times to 15 times) they had engaged in a series of behaviors related to social distancing (e.g., "In the last week, how often have you … attended a large social gathering … gone to a meeting in person" … etc.). However, in Study 1, 86% of participants reported engaging in zero of those behaviors within the last week, and in Study 2, 92% indicated as such. As a result of this extremely skewed distribution, such that the vast majority of responses were zero, these variables were not analyzed.
- Religiosity items: The original surveys included a number of items related to religiosity, including beliefs and practices regarding prayer, trust in the perceived accuracy of religious leaders in relation to COVID-19 information, and belief in God or other deities. We reserve the analyses of these items for a separate project, and thus do not include them here.
- Disgust ratings of images: In the Pilot Study, in addition to the images that constituted the pathogen disgust sensitivity images composite, participants ranked the disgustingness

of a variety of other images that lacked overt pathogen cues, such as images with endoparasite cues (e.g., wasps), and items that would not typically elicit disgust (e.g. an empty train, a soccer ball). We reserve the analyses of these items for a separate project, and thus do not include them here.

- In addition to these methodological differences, there was also a change to the pre-registered analytical strategy in Studies 1 and 2. More specifically, in the pre-registration we specified that we would use best subset selection and information criteria to compare the importance of different predictors. However, we later decided that this analytical approach was not suitable for testing the relative strength of the different political variables in associating with COVID-19 precautions. Instead, we decided to compare these variables within models by examining coefficients after accounting for the effects of the comparison variables. In sum, the conceptual goal of the pre-registered set of analyses—to compare the relative effects of different independent variables—was preserved, although the analytical techniques were changed.

Software version and source information

The reproducibility of the analyses found in the main text and supplement depend upon a set of R packages. It is possible that future updates to these packages could disrupt the functionality of the code. In anticipation of this possibility, we list the versions and sources of all required packages.

```
- Session info ---------------------------------------------------------------------
 setting  value
 version  R version 4.0.2 (2020-06-22)
 os       Windows 10 x64
 system   x86_64, mingw32
```

```
ui       RStudio
language (EN)
collate  English_United States.1252
ctype    English_United States.1252
tz       America/Denver
date     2020-08-06

- Packages -------------------------------------------------------------------------------
package       * version    date       lib source
abind           1.4-5      2016-07-21 [1] CRAN (R 4.0.0)
assertthat      0.2.1      2019-03-21 [1] CRAN (R 4.0.0)
backports       1.1.8      2020-06-17 [1] CRAN (R 4.0.0)
BayesFactor   * 0.9.12-4.2 2018-05-19 [1] CRAN (R 4.0.0)
bayestestR    * 0.7.2      2020-07-20 [1] CRAN (R 4.0.2)
boot            1.3-25     2020-04-26 [2] CRAN (R 4.0.2)
broom           0.7.0      2020-07-09 [1] CRAN (R 4.0.2)
callr           3.4.3      2020-03-28 [1] CRAN (R 4.0.0)
car           * 3.0-8      2020-05-21 [1] CRAN (R 4.0.0)
carData       * 3.0-4      2020-05-22 [1] CRAN (R 4.0.0)
cellranger      1.1.0      2016-07-27 [1] CRAN (R 4.0.0)
circlize      * 0.4.10     2020-06-15 [1] CRAN (R 4.0.2)
cli             2.0.2      2020-02-28 [1] CRAN (R 4.0.0)
coda          * 0.19-3     2019-07-05 [1] CRAN (R 4.0.0)
colorspace      1.4-1      2019-03-18 [1] CRAN (R 4.0.0)
correlation   * 0.3.0      2020-06-19 [1] CRAN (R 4.0.2)
corrplot      * 0.84       2017-10-16 [1] CRAN (R 4.0.0)
cowplot       * 1.0.0      2019-07-11 [1] CRAN (R 4.0.0)
crayon          1.3.4      2017-09-16 [1] CRAN (R 4.0.0)
curl            4.3        2019-12-02 [1] CRAN (R 4.0.0)
data.table      1.13.0     2020-07-24 [1] CRAN (R 4.0.2)
desc            1.2.0      2018-05-01 [1] CRAN (R 4.0.0)
devtools        2.3.1      2020-07-21 [1] CRAN (R 4.0.2)
digest          0.6.25     2020-02-23 [1] CRAN (R 4.0.0)
dplyr         * 1.0.0      2020-05-29 [1] CRAN (R 4.0.0)
effectsize    * 0.3.2      2020-07-27 [1] CRAN (R 4.0.2)
```

```
ellipsis        0.3.1      2020-05-15 [1] CRAN (R 4.0.0)
emmeans         1.4.8      2020-06-26 [1] CRAN (R 4.0.2)
estimability    1.3        2018-02-11 [1] CRAN (R 4.0.0)
evaluate        0.14       2019-05-28 [1] CRAN (R 4.0.0)
fansi           0.4.1      2020-01-08 [1] CRAN (R 4.0.0)
forcats         0.5.0      2020-03-01 [1] CRAN (R 4.0.0)
foreign         0.8-80     2020-05-24 [2] CRAN (R 4.0.2)
fs              1.4.2      2020-06-30 [1] CRAN (R 4.0.2)
generics        0.0.2      2018-11-29 [1] CRAN (R 4.0.0)
ggeffects       0.15.1     2020-07-27 [1] CRAN (R 4.0.2)
ggplot2       * 3.3.2      2020-06-19 [1] CRAN (R 4.0.2)
ggpmisc       * 0.3.7      2020-11-09 [1] CRAN (R 4.0.3)
ggpubr        * 0.4.0      2020-06-27 [1] CRAN (R 4.0.2)
ggridges        0.5.2      2020-01-12 [1] CRAN (R 4.0.0)
ggsignif        0.6.0      2019-08-08 [1] CRAN (R 4.0.0)
GlobalOptions   0.1.2      2020-06-10 [1] CRAN (R 4.0.2)
glue            1.4.1      2020-05-13 [1] CRAN (R 4.0.0)
GPArotation   * 2014.11-1  2014-11-25 [1] CRAN (R 4.0.0)
gridExtra     * 2.3        2017-09-09 [1] CRAN (R 4.0.0)
gtable          0.3.0      2019-03-25 [1] CRAN (R 4.0.0)
gtools          3.8.2      2020-03-31 [1] CRAN (R 4.0.0)
haven           2.3.1      2020-06-01 [1] CRAN (R 4.0.0)
hms             0.5.3      2020-01-08 [1] CRAN (R 4.0.0)
htmltools       0.5.0      2020-06-16 [1] CRAN (R 4.0.2)
httr            1.4.2      2020-07-20 [1] CRAN (R 4.0.2)
insight       * 0.9.0      2020-07-20 [1] CRAN (R 4.0.2)
interactions  * 1.1.3      2020-04-04 [1] CRAN (R 4.0.0)
jpeg          * 0.1-8.1    2019-10-24 [1] CRAN (R 4.0.0)
jtools          2.1.0      2020-06-23 [1] CRAN (R 4.0.0)
knitr           1.29       2020-06-23 [1] CRAN (R 4.0.0)
lattice       * 0.20-41    2020-04-02 [2] CRAN (R 4.0.2)
lifecycle       0.2.0      2020-03-06 [1] CRAN (R 4.0.0)
likert        * 1.3.5      2016-12-31 [1] CRAN (R 4.0.0)
lm.beta       * 1.5-1      2014-12-28 [1] CRAN (R 4.0.0)
lme4            1.1-23     2020-04-07 [1] CRAN (R 4.0.0)
```

```
magrittr        1.5        2014-11-22 [1] CRAN (R 4.0.0)
MASS          * 7.3-51.6   2020-04-26 [2] CRAN (R 4.0.2)
Matrix        * 1.2-18     2019-11-27 [2] CRAN (R 4.0.2)
MatrixModels    0.4-1      2015-08-22 [1] CRAN (R 4.0.0)
memoise         1.1.0      2017-04-21 [1] CRAN (R 4.0.0)
minqa           1.2.4      2014-10-09 [1] CRAN (R 4.0.0)
mnormt          2.0.1      2020-06-29 [1] CRAN (R 4.0.2)
modelr          0.1.8      2020-05-19 [1] CRAN (R 4.0.0)
munsell         0.5.0      2018-06-12 [1] CRAN (R 4.0.0)
mvtnorm         1.1-1      2020-06-09 [1] CRAN (R 4.0.0)
nlme            3.1-148    2020-05-24 [2] CRAN (R 4.0.2)
nloptr          1.2.2.2    2020-07-02 [1] CRAN (R 4.0.2)
openxlsx        4.1.5      2020-05-06 [1] CRAN (R 4.0.0)
pander          0.6.3      2018-11-06 [1] CRAN (R 4.0.0)
papaja        * 0.1.0.9997 2020-07-30 [1] Github (crsh/papaja@0457653)
parameters    * 0.8.2      2020-07-24 [1] CRAN (R 4.0.2)
pbapply         1.4-2      2019-08-31 [1] CRAN (R 4.0.0)
performance   * 0.4.8      2020-07-27 [1] CRAN (R 4.0.2)
pillar          1.4.6      2020-07-10 [1] CRAN (R 4.0.2)
pkgbuild        1.1.0      2020-07-13 [1] CRAN (R 4.0.2)
pkgconfig       2.0.3      2019-09-22 [1] CRAN (R 4.0.0)
pkgload         1.1.0      2020-05-29 [1] CRAN (R 4.0.0)
plyr            1.8.6      2020-03-03 [1] CRAN (R 4.0.0)
prettyunits     1.1.1      2020-01-24 [1] CRAN (R 4.0.0)
processx        3.4.3      2020-07-05 [1] CRAN (R 4.0.2)
ps              1.3.3      2020-05-08 [1] CRAN (R 4.0.0)
psych         * 1.9.12.31  2020-01-08 [1] CRAN (R 4.0.2)
purrr         * 0.3.4      2020-04-17 [1] CRAN (R 4.0.0)
R6              2.4.1      2019-11-12 [1] CRAN (R 4.0.0)
Rcpp            1.0.5      2020-07-06 [1] CRAN (R 4.0.2)
readxl        * 1.3.1      2019-03-13 [1] CRAN (R 4.0.0)
remotes         2.2.0      2020-07-21 [1] CRAN (R 4.0.2)
reshape2        1.4.4      2020-04-09 [1] CRAN (R 4.0.0)
rio             0.5.16     2018-11-26 [1] CRAN (R 4.0.0)
rlang           0.4.7      2020-07-09 [1] CRAN (R 4.0.2)
```

```
rmarkdown       2.3     2020-06-18 [1] CRAN (R 4.0.2)
rprojroot       1.3-2   2018-01-03 [1] CRAN (R 4.0.0)
rstatix         0.6.0   2020-06-18 [1] CRAN (R 4.0.2)
rstudioapi      0.11    2020-02-07 [1] CRAN (R 4.0.0)
rvest           0.3.6   2020-07-25 [1] CRAN (R 4.0.2)
scales          1.1.1   2020-05-11 [1] CRAN (R 4.0.0)
see           * 0.5.2   2020-07-27 [1] CRAN (R 4.0.2)
selectr         0.4-2   2019-11-20 [1] CRAN (R 4.0.0)
sessioninfo   * 1.1.1   2018-11-05 [1] CRAN (R 4.0.2)
shape           1.4.4   2018-02-07 [1] CRAN (R 4.0.0)
sjlabelled      1.1.6   2020-06-25 [1] CRAN (R 4.0.2)
sjmisc          2.8.5   2020-05-28 [1] CRAN (R 4.0.0)
sjPlot        * 2.8.4   2020-05-24 [1] CRAN (R 4.0.0)
sjstats         0.18.0  2020-05-06 [1] CRAN (R 4.0.0)
statmod         1.4.34  2020-02-17 [1] CRAN (R 4.0.0)
stringi         1.4.6   2020-02-17 [1] CRAN (R 4.0.0)
stringr       * 1.4.0   2019-02-10 [1] CRAN (R 4.0.0)
testthat        2.3.2   2020-03-02 [1] CRAN (R 4.0.0)
tibble        * 3.0.3   2020-07-10 [1] CRAN (R 4.0.2)
tidyr         * 1.1.0   2020-05-20 [1] CRAN (R 4.0.0)
tidyselect      1.1.0   2020-05-11 [1] CRAN (R 4.0.0)
tmvnsim         1.0-2   2016-12-15 [1] CRAN (R 4.0.0)
usethis         1.6.1   2020-04-29 [1] CRAN (R 4.0.0)
vctrs           0.3.2   2020-07-15 [1] CRAN (R 4.0.2)
wesanderson   * 0.3.6   2018-04-20 [1] CRAN (R 4.0.0)
withr           2.2.0   2020-04-20 [1] CRAN (R 4.0.0)
xfun            0.16    2020-07-24 [1] CRAN (R 4.0.2)
xml2            1.3.2   2020-04-23 [1] CRAN (R 4.0.0)
xtable        * 1.8-4   2019-04-21 [1] CRAN (R 4.0.0)
yaml            2.2.1   2020-02-01 [1] CRAN (R 4.0.0)
yarrr         * 0.1.5   2017-04-19 [1] CRAN (R 4.0.0)
zip             2.0.4   2019-09-01 [1] CRAN (R 4.0.0)
```

References

AllSides media bias ratings. (2020). AllSides. Retrieved July 29, 2020, from https://www.allsides.com/media-bias/media-bias-ratings

Aphalo, P. J. (2020). *ggpmisc: Miscellaneous Extensions to "ggplot2."* https://CRAN.R-project.org/package=ggpmisc

Auguie, B. (2017). *gridExtra: Miscellaneous functions for "grid" graphics*. https://CRAN.R-project.org/package=gridExtra

Aust, F., & Barth, M. (2020). *papaja: Create APA manuscripts with R Markdown*. https://github.com/crsh/papaja

Behrendt, S. (2014). *lm.beta: Add standardized regression coefficients to lm-objects*. https://CRAN.R-project.org/package=lm.beta

Bernaards, C. A., & Jennrich, R. I. (2005). Gradient projection algorithms and software for arbitrary rotation criteria in factor analysis. *Educational and Psychological Measurement, 65*, 676–696.

Bryer, J., & Speerschneider, K. (2016). *likert: Analysis and visualization Likert items*. https://CRAN.R-project.org/package=likert

Curtis, V., Aunger, R., & Rabie, T. (2004). Evidence that disgust evolved to protect from risk of disease. *Proceedings. Biological Sciences / The Royal Society*, *271 Suppl 4*, S131-3. https://doi.org/10.1098/rsbl.2003.0144

Dodd, M. D., Balzer, A., Jacobs, C. M., Gruszczynski, M. W., Smith, K. B., & Hibbing, J. R. (2012). The political left rolls with the good and the political right confronts the bad: Connecting physiology and cognition to preferences. *Philosophical Transactions of the*

Royal Society B: Biological Sciences, 367(1589), 640–649. https://doi.org/10.1098/rstb.2011.0268

Dunwoody, P., & Funke, F. (2016). The aggression-submission-conventionalism scale: Testing a new three factor measure of authoritarianism. *Journal of Social and Political Psychology, 4*, 571–600. https://doi.org/10.5964/jspp.v4i2.168

Fox, J., & Weisberg, S. (2019). *An R companion to applied regression* (Third). Sage. https://socialsciences.mcmaster.ca/jfox/Books/Companion/

Henry, L., & Wickham, H. (2020). *purrr: Functional programming tools*. https://CRAN.R-project.org/package=purrr

Kassambara, A. (2020). *ggpubr: "ggplot2" based publication ready plots*. https://CRAN.R-project.org/package=ggpubr

Kassambara, A. (2020). *rstatix: Pipe-Friendly Framework for Basic Statistical Tests*. https://CRAN.R-project.org/package=rstatix

Long, J. A. (2019). *interactions: Comprehensive, user-friendly toolkit for probing interactions*. https://cran.r-project.org/package=interactions

Lüdecke, D. (2020). *sjPlot: Data visualization for statistics in social science*. https://CRAN.R-project.org/package=sjPlot

Lüdecke, D., Ben-Shachar, M. S., & Makowski, D. (2020). Describe and understand your model's parameters. *CRAN*. https://doi.org/10.5281/zenodo.3731932

Müller, K., & Wickham, H. (2020). *tibble: Simple data frames*. https://CRAN.R-project.org/package=tibble

Phillips, N. (2017). *Yarrr: A companion to the e-Book "YaRrr!: The Pirate's Guide to R."* https://CRAN.R-project.org/package=yarrr

Pratto, F., Çidam, A., Stewart, A. L., Zeineddine, F. B., Aranda, M., Aiello, A., Chryssochoou, X., Cichocka, A., Cohrs, J. C., Durrheim, K., Eicher, V., Foels, R., Górska, P., Lee, I.-C., Licata, L., Liu, J. H., Li, L., Meyer, I., Morselli, D., … Henkel, K. E. (2013). Social dominance in context and in individuals: Contextual moderation of robust effects of social dominance orientation in 15 languages and 20 countries. *Social Psychological and Personality Science*, *4*(5), 587–599. https://doi.org/10.1177/1948550612473663

Ram, K., & Wickham, H. (2018). *wesanderson: A Wes Anderson palette generator*. https://CRAN.R-project.org/package=wesanderson

Revelle, W. (2019). *psych: Procedures for psychological, psychometric, and personality Research*. Northwestern University. https://CRAN.R-project.org/package=psych

Tybur, J., Lieberman, D., & Griskevicius, V. (2009). Microbes, mating, and morality: Individual differences in three functional domains of disgust. *Journal of Personality and Social Psychology*, *97,* 103–122. https://doi.org/10.1037/a0015474

Venables, W. N., & Ripley, B. D. (2002). *Modern applied statistics with S* (Fourth). Springer. http://www.stats.ox.ac.uk/pub/MASS4

Wei, T., & Simko, V. (2017). *R package "corrplot": Visualization of a correlation matrix*. https://github.com/taiyun/corrplot

Wickham, H. (2016). *ggplot2: Elegant graphics for data analysis*. Springer-Verlag New York. https://ggplot2.tidyverse.org

Wickham, H. (2019). *stringr: Simple, consistent wrappers for common string operations*. https://CRAN.R-project.org/package=stringr

Wickham, H., & Bryan, J. (2019). *readxl: Read Excel files*. https://CRAN.R-project.org/package=readxl

Wickham, H., François, R., Henry, L., & Müller, K. (2020). *dplyr: A grammar of data manipulation*. https://CRAN.R-project.org/package=dplyr

Wickham, H., & Henry, L. (2020). *tidyr: Tidy messy data*. https://CRAN.R-project.org/package=tidyr

Wilke, C. O. (2019). *cowplot: Streamlined plot theme and plot annotations for "ggplot2."* https://CRAN.R-project.org/package=cowplot

Wilson, G. D., & Patterson, J. R. (1968). A new measure of conservatism. *British Journal of Social and Clinical Psychology*, *7*(4), 264–269. https://doi.org/10.1111/j.2044-8260.1968.tb00568.x
