# Supplementary material for: Of pathogens and party lines: Social conservatism positively associates with COVID-19 precautions among U.S. Democrats but not Republicans
Source: PLoS One. 2021 Jun 29;16(6):e0253326. doi: 10.1371/journal.pone.0253326 (PMC8241032; doi:10.1371/journal.pone.0253326)
Supplement: S2 Appendix — (PDF) [file pone.0253326.s002.pdf]

S2 APPENDIX – PILOT STUDY TO: Social Conservatism Positively Associates with COVID-19 Precaution among Democrats but not Republicans

Table of Contents

Pilot Study materials and methods 3

Pilot Study results 9

Pilot Study discussion 16

References 20

Pilot Study materials and methods

Sample size: Previous studies concerning the relationship between pathogen threat sensitivity and measures of socially conservative political attitudes report correlations of r = .10-.49 (Md = .24) (Karinen et al., 2019; Terrizzi et al., 2013; Tybur et al., 2016). Corresponding power analyses for sample size estimation indicate that, for $\alpha = .05$ and a power = .80, the sample size needed is between approximately 30 and 782, with n = 134 corresponding to the median effect size. Because a) the current studies utilize novel measures of pathogen threat avoidance that specifically relate to the COVID-19 outbreak, rather than the disgust sensitivity measures that have primarily been used in previous research, and b) we are interested in differences between participants who identify with different political parties (which necessitates subdividing our sample), within pragmatic constraints of time and available funding, we aimed at the higher end of the broad range of projected sample sizes.

Participants: 522 adult U.S. participants were recruited on Amazon's Mechanical Turk platform in exchange for $ 3.00 (20-minute HIT, accepting workers with a 99% approval rating and at least 500 completed HITS). The payment included a $ 0.25 bonus (included in the $ 3.00 payment) in consideration of exposure to a series of mildly aversive disgust images. Data were prescreened for repeat participation using IP addresses, minimum completeness, minimum completion time, and answers to "catch questions". The final sample consisted of 433 adults (46% female; 76% white) ranging in age from 18-78 (M = 38.4, SD = 11.9). Participants were recruited on April 17th, 2020, when all but 5 US states had stay-at-home or safer-at-home orders ("U.S. state and local government responses to the COVID-19 pandemic", n.d.).

Measures:

Political orientation*:* Political orientation was measured identically to Studies 1 and 2, using the modified version of Dodd et al.'s (2013) version of Wilson and Patterson's (1968) issues index.

Right-wing authoritarianism and social dominance orientation*:* Right-wing authoritarianism and its three subscales–traditionalism, endorsement of authoritarian aggression, and submission to authority–and social dominance orientation were measured as in Studies 1 and 2. The combined socially conservative attitudes scale was again calculated as the z-scored composite between traditionalism and social conservatism. This composite was reliable, $\alpha = .89$.

Precautionary COVID-19 behaviors: Using 7-point Likert scales, the pilot precautionary COVID-19 behaviors scale measure consisted of 10 questions, including the frequency of mask wearing, hand washing, physically distancing, and disinfecting, and the importance to the participant of stocking up on supplies such as hand sanitizer and household disinfectants. Items were rated on 7-point scales, from either "never" to "as often as possible". Or from "not important at all", to "extremely important". Participants were also asked the extent to which they were following local lockdown restrictions. Averaging together participants' responses to these items, we created a composite Pilot Study measure for precautionary COVID-19 behaviors. This composite was reliable, $\alpha = .89$. Note that this composite, while largely similar to the precautionary behaviors composite used in Studies 1 and 2, has slight differences. In the Pilot Study, participants were not asked about the importance of stocking up on masks, the frequency with which supplements were taken with the intention of boosting the immune system, and the extent to which they were careful to maintain distance from people outside the household. Further, in the Pilot Study, participants were asked about how important it was that they stocked

up on hand sanitizer and hand soap separately, while in Studies 1 and 2, those two household goods were collapsed into a single item. The differences between the Pilot Study, and Studies 1 and 2, can be attributed to scale development refinements that were made after the Pilot Study. In particular, we wanted to broaden the scale to include additional precautions such as the use of supplements, as well as improve how we asked about social distancing, and the importance of mask wearing. Despite these small differences in the precautionary behaviors scale between the Pilot Study, and Studies 1 and 2, there is a substantial degree of actual and conceptual overlap, such that the results of the Pilot Study can be conceptually compared to those of the main text studies.

Other COVID-19-related items: In addition to the precautionary behaviors enumerated above, we asked participants a series of questions concerning other aspects of their beliefs and experiences regarding the COVID19 outbreak. These questions broadly fell into a series of different categories.

First, on a 7-point Likert scale from “not at all protective” to “extremely protective”, we asked participants to rate the *effectiveness in protecting against COVID-19* of a variety of different behaviors, such as the use of hydroxychloroquine, handwashing, and mask-wearing.

Second, participants were asked a series of questions that gauged the relative hazards posed by different threats caused by the COVID-19 outbreak. In particular, we measured how participants weighed the perceived threat of the direct health hazards posed by the pandemic relative to the downstream economic costs. Using 1 to 7 Likert type scales, health-domain items included concerns about contracting and transmitting COVID-19, the estimated risk posed by the disease to one’s own health as well as the health of those them, as well as questions regarding whether

participants thought the threat of the pandemic was overblown, or would quickly pass. Economic related items included self-reported anxiety over the financial repercussions of the pandemic, and beliefs that the economic costs of the outbreak outweighed the public health costs. We created a reliable ($\alpha = .82$) *COVID-19 domain-specific threat-assessments* based on these items. Health domain items were reverse scored, such that higher scores indicated finding the direct health consequences of the pandemic less serious, particularly in contrast to downstream threats to the economy. Note that the version of this scale used in the Pilot Study differed substantially from that used in Studies 1 and 2. In the Pilot Study, there were fewer items – and they were less granular – measuring various health concerns; additionally, concerns about personal liberty threats were not measured at all. These changes were made between the Pilot Study and Studies 1 and 2 as refinements in measuring the concepts of interest, and in recognition of the fact that many Americans were expressing grave concerns about personal liberty threats resulting from public health directives. However, despite these differences in the precise items and measures, conceptual comparisons can still be made between the Pilot Study and Studies 1 and 2, if not direct comparisons.

We also asked individual questions (not averaged into any composites) regarding the perceived likelihood of contracting COVID-19, and the severity of the economic consequences resulting from the pandemic.

Third, we asked participants whether they themselves had been infected with COVID-19, and, if so, whether they were still ill.

Fourth, we asked participants questions about *COVID-19 prevalence within their local communities*, including their perceptions of the current local prevalence of COVID-19, their neighborhood's density, and how many people they knew who had contracted COVID-19.

Fifth, participants provided *political leadership assessments related to the pandemic* using questions, rated on a 1 to 7 Likert scale from "worst possible response" to "best possible response", about the effectiveness of the President, Congress, and the participant's state and local governments in their responses to the outbreak.

Trust in sources of COVID-19 information: To examine whether partisan differences in the assessments of the accuracy of different information sources tracked participants' reactions to the COVID-19 outbreak, we asked participants how strongly they trusted politicians, scientists, religious leaders, journalists, alternative news sources, social media, and friends and family in regard to information about the outbreak, rated on a 1-7 Likert scale from, "strongly distrust", to "strongly trust". These trust items were substantially different than those used in Studies 1 and 2. In those studies, participants were provided with a much longer list of sources, and instead of only falling into maximally general categories such as, "scientists" or "politicians", items included specific individuals, organizations, and more narrowly defined groups of people. Additionally, many of the sources used in the main text studies had explicit partisan leanings. Further, instead of asking about "trust" directly, the phrasing in Studies 1 and 2 asked how much participants thought those sources of information provided advice based on accurate information about what to do during the COVID-19 outbreak. These changes were made after the Pilot Study in order to measure trust in a more granular manner, including in terms of partisanship. Therefore, although there is some conceptual comparability between the Pilot and main text

studies with these items (for example, the single item "trust in scientists" question in the Pilot Study is broadly comparable to the multi-item composite trust in scientists measure employed in Studies 1 and 2), the main text studies represented a substantial change and improvement over the simpler measures used in the Pilot Study, and comparisons are not always possible.

Disgust Sensitivity: Participants completed the 7-item pathogen subscale of the Three-Domain Disgust Scale (TDDS) (Tybur et al., 2009), and a modified version of Curtis et al.'s (2004) visual disgust sensitivity measure, in counterbalanced order of presentation. The pathogen subscale of the TTDS asks participants to rate how disgusting they find a series of hypothetical scenarios, such as stepping on dog feces or accidentally touching a person's bloody cut. The Curtis et al. image stimuli present participants with a series of 19 images varying in the presence of pathogen cues, such as an open wound or a sickly-looking person; participants rate how disgusting they find each image. Both instruments use a 7-point Likert scale, from "not disgusting at all", to "extremely disgusting". Two disgust sensitivity composites were created by averaging TDDS responses, and the responses to the disgust images, respectively. Because results were conceptually similar whether using the TDDS or disgust images scales, in Studies 1 and 2, only the TDDS was used, in the interest of minimizing survey length.

News consumption: To examine whether media habits were associated with partisan differences in responses to the COVID-19 outbreak, we queried participants about their news consumption. First, we asked participants how many hours they spent per week consuming news of any kind, and we then broke that down into more specific types of news media, asking how many hours per week the participant watched, listened to, or read local newspapers, national newspapers, public radio, local television news, etc. We did not ask questions about specific media outlets,

nor did we ask about the partisan leanings of participants' news consumption. This was an omission considering that this work is explicitly interested in whether consumption of different kinds of media sources–particularly in regard to their partisan leanings–may influence politicized differences in COVID-19 reactions. Therefore, in Studies 1 and 2, we added a large battery of more granular items addressing partisan news sources.

Demographics and study checks: Finally, participants answered demographic questions, including their gender identity, ethnicity, age, ZIP code, religious belief, income, education level, and preferred U.S. political party. Note that, out of error, "Independent" was not an option on the political party affiliation question. Check questions included simple attention checks, whether distractions occurred while the participant was taking the survey, a self-report question on whether the participant paid attention during the study, and an English comprehension check that asked participants to write a short sentence or two.

Pilot Study results

Does COVID-19 precautionary behavior differ by political party?

227 participants self-identified as Democrat, 118 self- identified as Republican, and 88 self- identified as either members of the Green party, Libertarian party, Constitutionalist party, or other. Because our recruitment method did not capture large samples of third-party supporters, and because partisan differences between Democrats and Republicans broadly capture wide-ranging variation along different liberalism-conservatism dimensions, we examine party-specific effects only for the two major parties. We find no significant baseline differences in mean precautionary behaviors between Republicans (M = 5.05, SD = 1.31) and Democrats (M = 5.20, SD = 1.17) in our sample (t[215.68] = -1.08, p = .282). These results diverge from those of

Studies 1 and 2, where Democrats–on average–engage in significantly more precautions than did Republicans. Note that because "Independent" was accidentally excluded as an option on the political party affiliation question in the Pilot Study, we can only compare between Democrats and Republicans–but not Independents–in this and further analyses.

Do socially conservative political attitudes predict precautionary behavior?

Our composite measure of precautionary behaviors in response to the COVID-19 outbreak was correlated with socially conservative political attitudes among Democrats, but not Republicans (Figure S1). Therefore, the prediction made by the traditional norms account—that social conservatism and traditionalism should correlate with pathogen avoidance—is observed, but only among the subset of Democrat participants. This is consistent with the results from Studies 1 and 2.

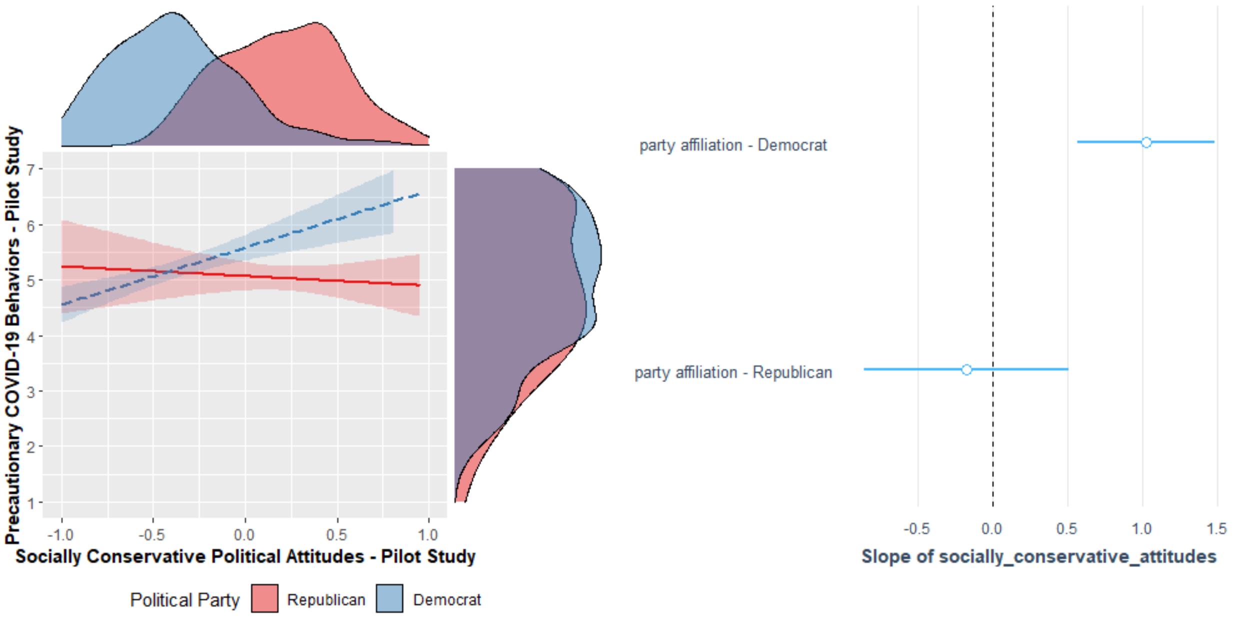

Figure S1. Pilot study conditional effects of a moderated linear regression in which COVID-19 precautions were regressed on the (centered) socially conservative attitudes composite, political party affiliation, and their two-way interaction.

The left pane shows the conditional relationships between socially conservative attitudes and COVID-19 precautions by political party affiliation. Bands around regression lines are 95% confidence intervals. The density plot along the x-axis represents the raw distributions of socially conservative attitudes by political affiliation. The density plot along the y-axss represents the raw distributions of precautionary behaviors by political party.

The right pane plots the coefficients obtained from a simple slopes analysis of the effect of socially conservative attitudes for each level of party affiliation. Coefficients are unstandardized. Lines indicate 95% confidence intervals.

What factors drive differences in the relationship between socially conservative political attitudes and precautions between Republicans and Democrats?

In Study 1, we identified four variables that were jointly suppressing the relationship between socially conservative attitudes and precautionary behaviors among Republicans: economic conservatism, the trust in scientists composite, the trust in liberals and moderates composite, and the liberal media consumption composite. In the Pilot Study, we measured economic conservatism, but not any of the trust or media consumption composites. However,

there was a single item measuring trust in scientists, which is conceptually related to the composite measure used in the main text analyses. Therefore, as a conceptual if not direct replication, we tested whether economic conservatism and the single trust in scientists item jointly suppressed the relationship between socially conservative attitudes and precautionary behaviors among Republicans.

First, the combined indirect effect of those two variables was negative and significant among Republicans (bootstrapped unstandardized indirect effect = -.50, 95% CI [-.96, -.13]), demonstrating suppression. Second, accounting for the suppressors curtailed the significant moderation of political party on the relationship between socially conservative attitudes and precautionary behaviors, such that there was no longer a significant difference in slopes between Republicans and Democrats (B = 0.59, p = .212). Third, a simple slopes analysis was performed to ascertain the conditional effects of socially conservative attitudes on precautionary behaviors, accounting for the effects of the suppressors. Socially conservative political attitudes remained a significant positive correlate of precautions among Democrats (B = 1.04, t(327) = 3.89, p = 1.23e-4). However, the conditional effect was not significant among Republicans (B = 0.45, t(327) = 1.17, p = .243).

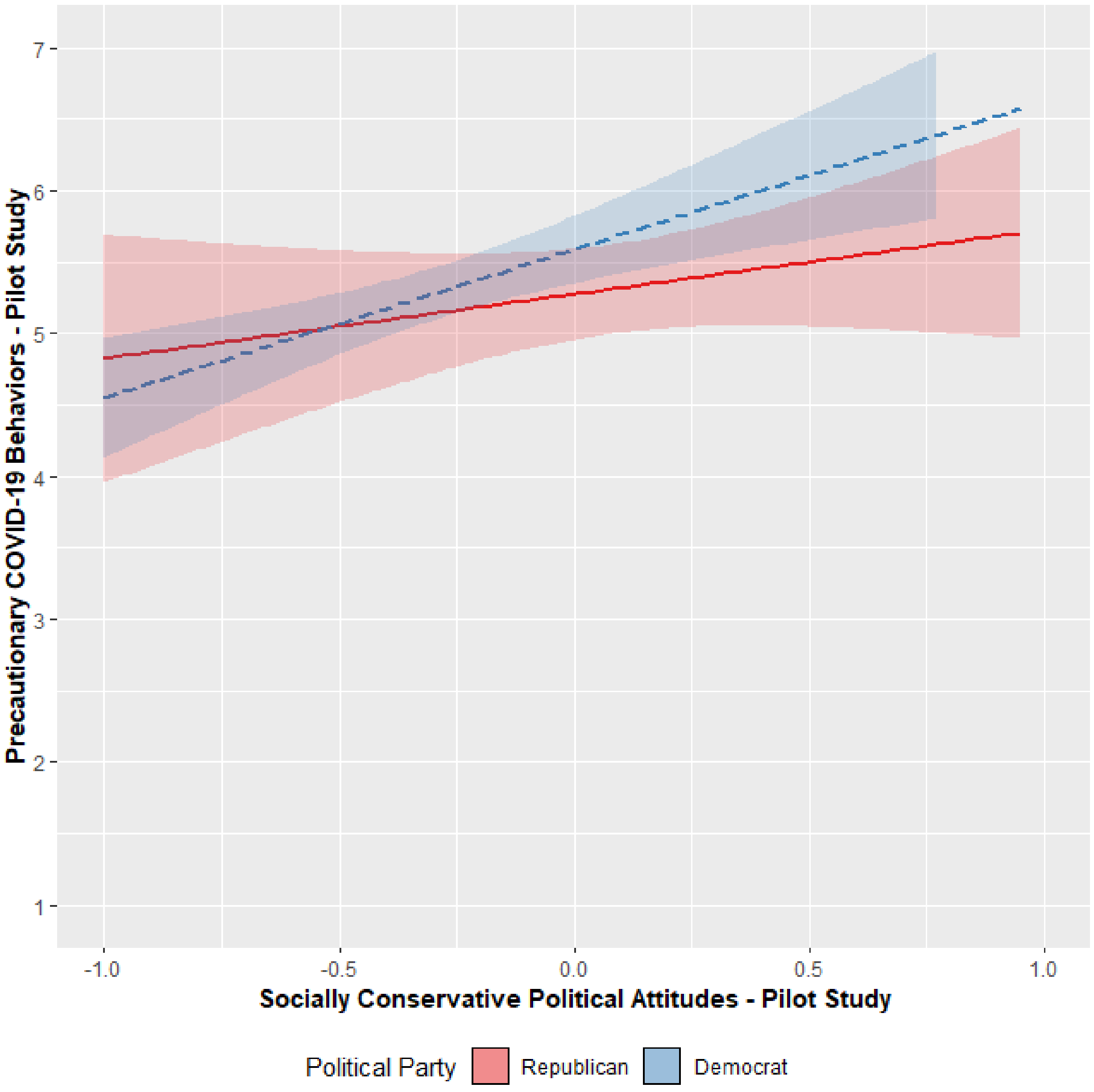

Figure S2. Pilot study conditional effects of a moderated linear regression in which the two Pilot Study suppressor variables were added to the model specified in Figure S1. Further, each of these suppressor variables interacted with political party affiliation in the model. Bands around regression lines are 95% confidence intervals.

In addition, the party-specific precautions-socially conservative attitudes relationships were conceptually unchanged by adding the full suite of demographic variables and other covariates described in the Main Text.

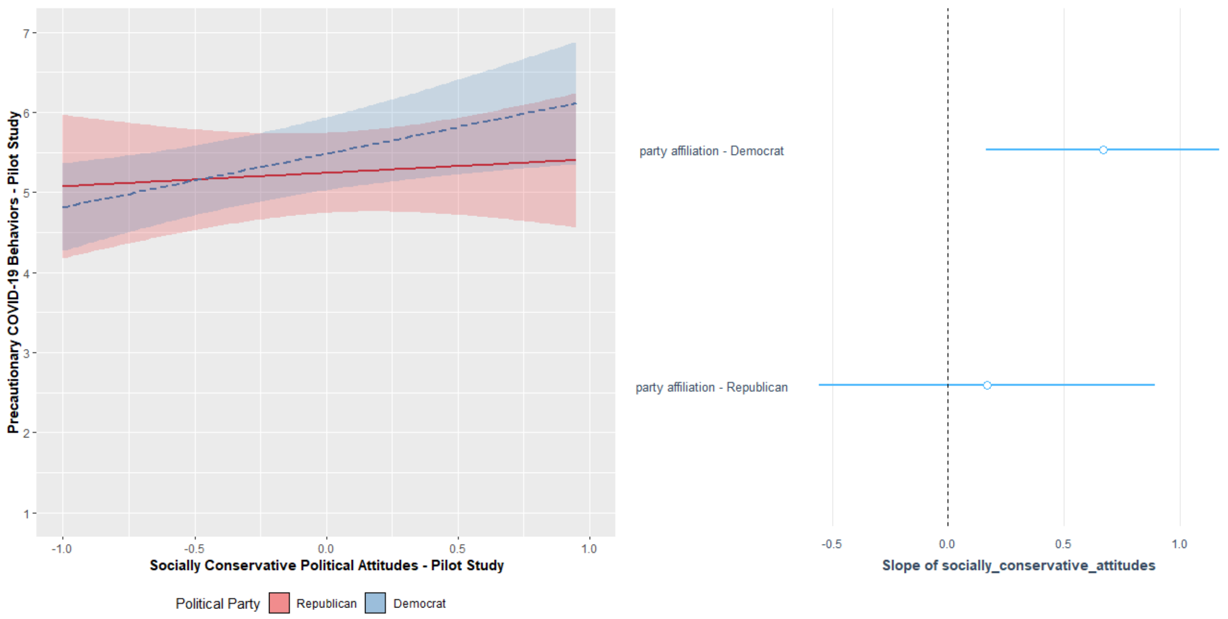

Figure S3. Pilot Study conditional effects of a moderated linear regression, in which a wide number of covariates were added to the suppression model specified in Figure S2. These additional covariates were as follows: age, gender, ethnicity, income, education, pre-existing health conditions, self-reported density of local neighborhood, self-reported estimates of local COVID-19 prevalence, the extent to which one's job required leaving the household, and the two measures of pathogen disgust sensitivity.

The left pane shows the conditional relationship between socially conservative attitudes and COVID-19 precautions by political party, after accounting for both the effects of the two Pilot Study suppressor variables, as well as the additional covariates. Bands around regression lines are 95% confidence intervals.

The right pane plots the coefficients obtained from a simple slopes analysis of the effect of socially conservative attitudes for each level of party affiliation. Coefficients are unstandardized. Lines indicate 95% confidence intervals.

These findings are largely conceptually consistent with the results from Studies 1 and 2, but with the important caveats that the measured suppressor variables were substantially different between the Pilot Study and the main text studies, and that the conditional effect of socially conservative attitudes on precautionary behaviors did not obtain among Republicans.

Disgust sensitivity, politics, and precautionary COVID-19 behaviors

Using linear regression with moderation, after accounting for potential interactions between political party affiliation and pathogen disgust sensitivity, both disgust sensitivity composites associated with precautionary behaviors among Democrats (TDDS: B = .27, p = 2.00e-5; Disgust images scale: B = .32, p = 1.69e-6), and Republicans (TDDS: B = .40, p = 5.48e-5; Disgust images scale: B = .42, p = 2.22e-4). The interactions between political party and pathogen disgust sensitivity were not significant (Bs = -.14 – -.10, ps = .242 – .456). These results are consistent with Studies 1 and 2.

Further, both measures of disgust sensitivity associated with socially conservative attitudes among Democrats (see Figure S4). However, whereas disgust sensitivity did not associate with socially conservative attitudes among Republicans in either Studies 1 or 2, this association did obtain in the Pilot Study, consistent with prior literature on political differences in pathogen avoidance.

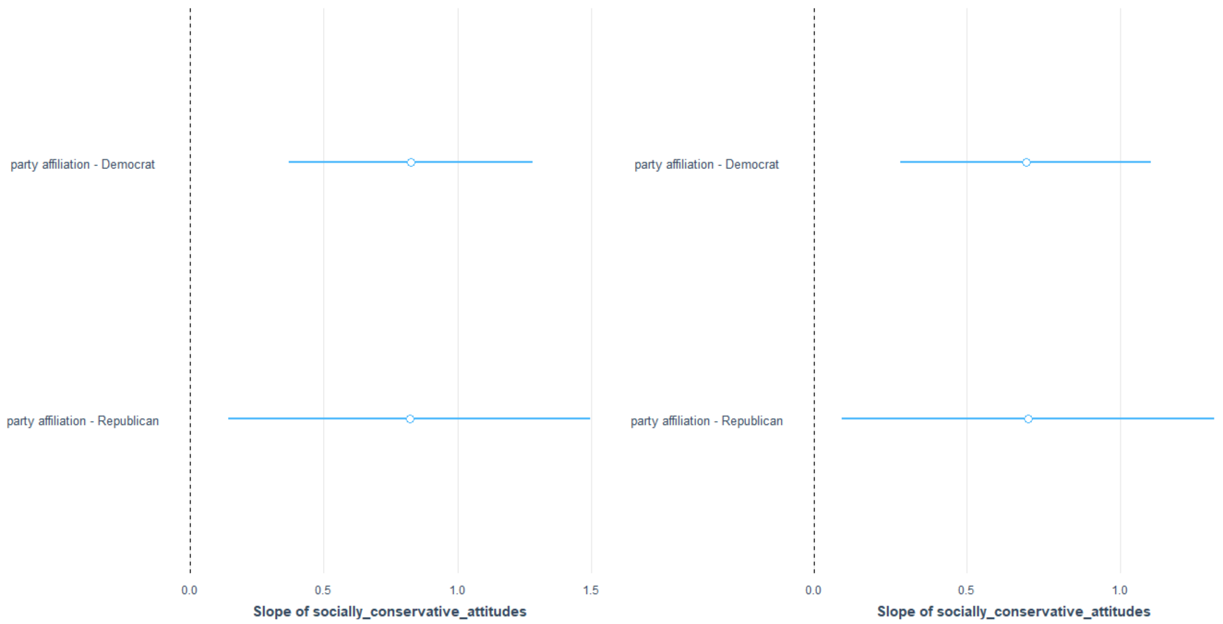

Figure S4. Pilot Study forest plots showing conditional effects (based on simple slopes analyses) of moderated linear regressions, in which COVID-19 precautionary behaviors was regressed separately on either pathogen disgust sensitivity measured using the three domain disgust scale (left pane) or pathogen disgust measured using the disgust images scale (right pane), political party affiliation, and the two-way interaction between party affiliation and the particular disgust sensitivity variable. Plotted coefficients are unstandardized. Lines indicate 95% confidence intervals.

Additionally, there was a significant difference in average disgust responses between Democrats (TDDS: M = 4.69, SD = 1.26; Disgust images scale: M = 4.31, SD = 1.17) and Republicans (TDDS: M = 4.99, SD = 1.11; Disgust images scale: M = 4.64, SD = .95) along both pathogen disgust sensitivity scales (TDDS: t[256.52] = 2.31, p = .021; Disgust images scale: t[281.55] = 2.78, p = .006).

Pilot Study discussion

The overall pattern of findings observed in the Pilot Study are consistent with those found in Studies 1 and 2. The predicted pattern between socially conservative attitudes and precautionary COVID-19 behaviors obtained among Democrats, but not Republicans. Further, among Democrats, socially conservative attitudes were consistently the strongest positive

correlate of precautions, compared to other dimensions of political ideology and attitudes. Further, although the Pilot Study did not include the trust and media composites found in Studies 1 and 2, thus precluding direct comparison, there was some evidence that economic conservatism and a single-item trust-in-scientists question suppressed the precautions-socially conservative attitudes relationship among Republicans, consistent with the main text studies.

However, it is of some interest that average precautionary behaviors did not significantly differ between Republicans and Democrats in the Pilot Study, contrary to the results from Studies 1 and 2. Although political dynamics have been at play since the start of the COVID-19 outbreak, political polarization concerning responses to the pandemic have increased over time (Pew Research Center, 2020), including in the intervening periods between the Pilot Study and Studies 1 and 2. We speculate that our results may reflect a canalization of a partisan difference in precautions over time, such that gaps in prophylaxis widened between supporters of different political affiliations. Alternatively, the null results in the Pilot Study may reflect a false negative.

Two critical limitations of the Pilot Study were the small number of Republicans in the sample, and the lack of an Independent option on the political affiliation question. If Independents, on average, occupy an ideological middle ground between Republicans and Democrats, this omission constituted a substantial missed opportunity to examine the relationship between precautionary behavior and political attitudes. In Studies 1 and 2, we addressed these limitations by increasing the overall recruitment target–hence increasing the number of Republicans in the samples– and adding “Independent” as a political party option. Additionally, although we were interested in how partisan differences in trust and news consumption choices might shape responses to the COVID-19 outbreak–particularly in regard to

their potential suppressive effects on the precautions-socially conservative attitudes relationship–in the Pilot Study we failed to measure participants' trust for information sources aligned or misaligned with their political ideologies. Instead, we asked questions about, for instance, whether they trusted journalists in general, rather than journalists with specific partisan perspectives. Likewise, we did not ask about consumption of any particular news outlets, or outlets that varied in their partisanship (e.g., Fox News, MSNBC). To address these issues in Studies 1 and 2, we used more granular trust and media consumption items that considered the partisanship of the various sources, and differences between individual actors and larger institutions, and also attended more to issues regarding trust in different kinds of scientific sources.

Further, in the Pilot Study we failed to adequately measure attitudes toward non-health threats posed by the COVID-19 outbreak, such as those posed by economic crisis or the perceived loss of personal liberties. Given our interest in examining trade-offs in concern and precautions for different domains of threat related to the pandemic, it was important to measure estimates of those non-health threats in more detail. Although the Pilot Study included several measures addressing perceived economic risks, we did not systematically gauge either risk assessments or precautionary behaviors in response to the non-health consequences of the COVID-19 outbreak. Therefore, in Studies 1 and 2, we posed a systematic set of questions along these lines, such as participants' degree of concern about losing individual liberties, and whether participants were preparing for an economic downturn.

Finally, in light of the preliminary results from the Pilot Study, we considered the possibility that dynamics concerning perceived government overreach vis-a-vis the coronavirus

outbreak may be associated with preferences for individual liberties and small government, as indexed by economic conservatism. These kinds of attitudes may shape politicized responses to the pandemic. To further probe this possibility in Studies 1 and 2, we included a short, face-valid scale that measured opinions about government public health policy in relation to smoking. This allowed a more direct measure of attitudes concerning another real-world public health intervention, affording a test of the possibility that skepticism of public health mandates in a general sense might play a role in the interaction between socially conservative attitudes, political party, and precautionary behaviors.

References

Curtis, V., Aunger, R., & Rabie, T. (2004). Evidence that disgust evolved to protect from risk of disease. *Proceedings. Biological Sciences / The Royal Society*, *271 Suppl 4*, S131-3. https://doi.org/10.1098/rsbl.2003.0144

Dodd, M. D., Balzer, A., Jacobs, C. M., Gruszczynski, M. W., Smith, K. B., & Hibbing, J. R. (2012). The political left rolls with the good and the political right confronts the bad: Connecting physiology and cognition to preferences. *Philosophical Transactions of the Royal Society B: Biological Sciences*, *367*(1589), 640–649. https://doi.org/10.1098/rstb.2011.0268

Karinen, A. K., Molho, C., Kupfer, T. R., & Tybur, J. M. (2019). Disgust sensitivity and opposition to immigration: Does contact avoidance or resistance to foreign norms explain the relationship? *Journal of Experimental Social Psychology*, *84*, 103817. https://doi.org/10.1016/j.jesp.2019.103817

Pew Research Center. (2020). *Republicans, Democrats move even further apart in coronavirus concerns*. https://www.pewresearch.org/politics/2020/06/25/republicans-democrats-move-even-further-apart-in-coronavirus-concerns/

Terrizzi, J. A., Shook, N. J., & McDaniel, M. A. (2013). The behavioral immune system and social conservatism: A meta-analysis. *Evolution and Human Behavior, 34*(2), 99–108. https://doi.org/10.1016/j.evolhumbehav.2012.10.003

Tybur, J., Lieberman, D., & Griskevicius, V. (2009). Microbes, mating, and morality: Individual differences in three functional domains of disgust. *Journal of Personality and Social Psychology*, *97*, 103–122. https://doi.org/10.1037/a0015474

Tybur, J. M., Inbar, Y., Aarøe, L., Barclay, P., Barlow, F. K., Barra, M. de, Becker, D. V., Borovoi, L., Choi, I., Choi, J. A., Consedine, N. S., Conway, A., Conway, J. R., Conway, P., Adoric, V. C., Demirci, D. E., Fernández, A. M., Ferreira, D. C. S., Ishii, K., … Žeželj, I. (2016). Parasite stress and pathogen avoidance relate to distinct dimensions of political ideology across 30 nations. *Proceedings of the National Academy of Sciences*, *113*(44), 12408–12413. https://doi.org/10.1073/pnas.1607398113

U.S. state and local government responses to the COVID-19 pandemic. (2020). In *Wikipedia*. https://en.wikipedia.org/w/index.php?title=U.S._state_and_local_government_responses_to_the_COVID-19_pandemic&oldid=970939067

Wilson, G. D., & Patterson, J. R. (1968). A new measure of conservatism. *British Journal of Social and Clinical Psychology*, *7*(4), 264–269. https://doi.org/10.1111/j.2044-8260.1968.tb00568.x
