# Supplementary material for: Of pathogens and party lines: Social conservatism positively associates with COVID-19 precautions among U.S. Democrats but not Republicans
Source: PLoS One. 2021 Jun 29;16(6):e0253326. doi: 10.1371/journal.pone.0253326 (PMC8241032; doi:10.1371/journal.pone.0253326)
Supplement: S3 Appendix — (PDF) [file pone.0253326.s003.pdf]

S3 APPENDIX – ANALYSES SUPPORTING MAIN TEXT TO: Social Conservatism Positively Associates with COVID-19 Precaution among Democrats but not Republicans

Contents

1. Exploratory factor analysis of trust variables 3

2. Party-specific relationships between COVID-19 precautions and ideological measures 5

4. Correlations between political measures 24

5. Robustness of results after including covariates.. 25

6. Are the relationships between socially conservative attitudes and COVID-19 precautions attributable to other dimensions of political ideology?.. 28

7. Relationships between socially conservative attitudes and pathogen disgust sensitivity...... 30

8. Determining whether liberal media consumption, trust in scientists, trust in liberals and moderates, and economic conservatism suppress the relationship between socially conservative attitudes and disgust sensitivity among Republicans and Independents...................................... 32

9. Relationship between economic conservatism and COVID-19 threat concerns among Republicans... 33

10. Political affiliation differences in economic precautions.. 35

11. Perceived health threat of COVID-19 and suppressor variables 36

References 40

1. Exploratory factor analysis of trust variables

In Study 1, we included items that measured participants' confidence in a wide range of different sources of information concerning COVID-19. We ran an exploratory factor analysis in order to determine the factor structure of these items, which included media figures, media organizations, health professionals, health organizations, politicians, and broad categories of people; these categories spanned the ideological spectrum. First, we used the Kaiser-Meyer-Olkin (KMO) and Bartlett's tests to determine whether these items were suitable for structure detection. The KMO test suggested that the strength of the relationships among the variables was high (KMO = .94), and Bartlett's test was significant ($\chi^2(276) = 20015.04$, $p < .001$), suggesting that the use of factor analysis was appropriate.

The R package parameters (Lüdecke, Ben-Shachar & Makowski, 2020). was then used to determine how many factors to extract. There was the most agreement between methods for a three factor solution.

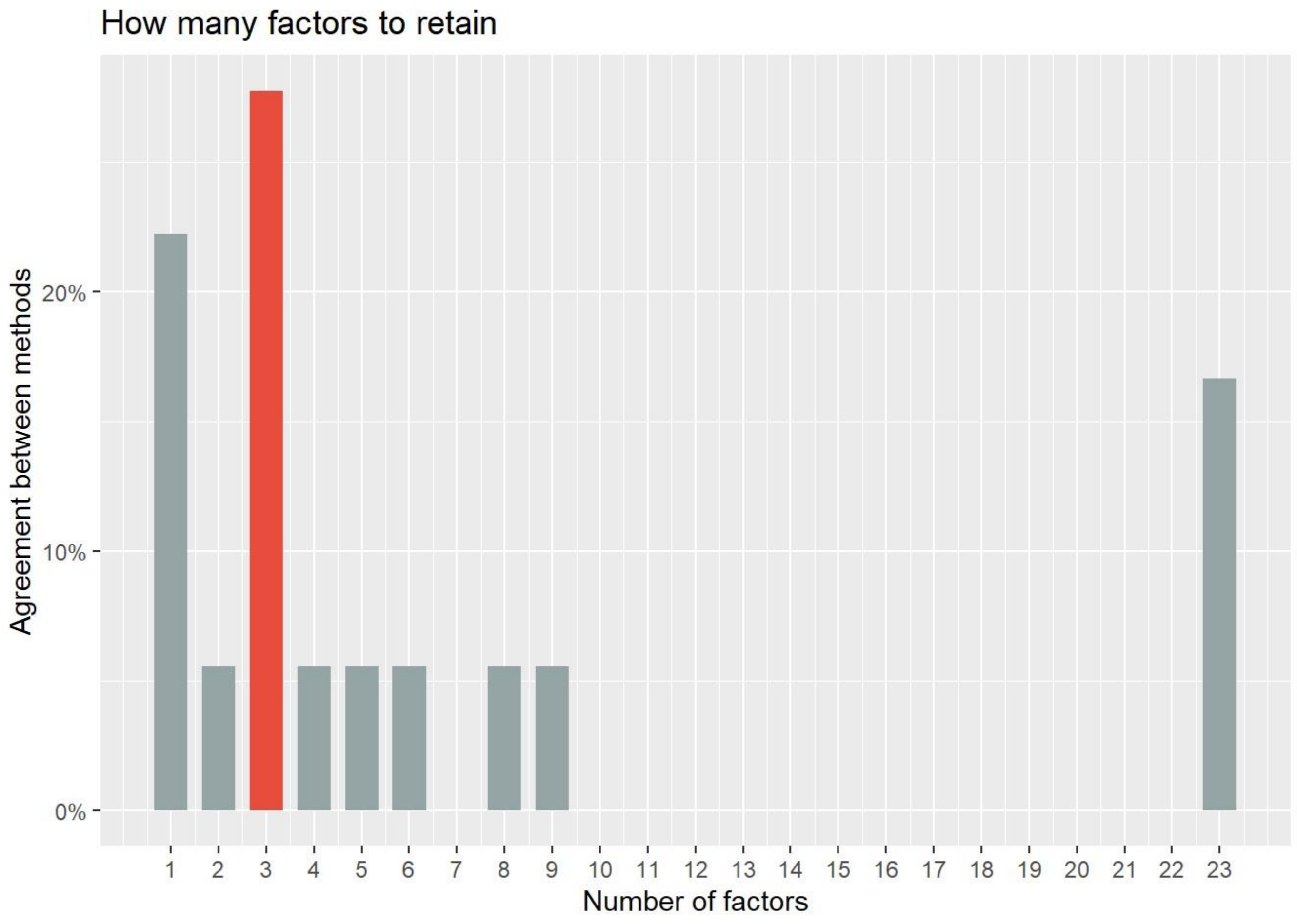

Figure S5. Graphical representation showing agreement between different methods for determining the number of factors to retain.

A factor analysis was conducted with minimum residual extraction, oblimin rotation, and a polychoric correlation matrix. Three factors were extracted. The three factor values had sums of squared loadings of 6.83, 5.98, and 3.68, and explained 30%, 26%, and 16% of the variance, respectively. When extracted, these three factors were conceptually coherent (see Table S4). For each factor, items with factor loadings greater than .60 were averaged together, producing the composite measures used in analyses. We labeled these factors, “trust in liberals and moderates”, “trust in conservatives”, and “trust in scientists”.

```
## Standardized loadings (pattern matrix) based upon correlation matrix
##                                         MR1   MR2   MR3   h2    u2 com
## covid_accuracy_liberaljournalists       0.78 -0.23  0.08 0.78 0.219 1.2
## covid_accuracy_fauci                    0.17 -0.15  0.71 0.74 0.259 1.2
## covid_accuracy_trump                   -0.19  0.85 -0.01 0.79 0.214 1.1
## covid_accuracy_cdc                     -0.05  0.07  0.84 0.64 0.363 1.0
## covid_accuracy_msnbc                    0.79 -0.12  0.15 0.80 0.196 1.1
## covid_accuracy_fox                      0.02  0.90  0.05 0.80 0.201 1.0
## covid_accuracy_breitbart                0.15  0.85 -0.15 0.77 0.229 1.1
## covid_accuracy_hannity                 -0.02  0.95  0.02 0.91 0.091 1.0
## covid_accuracy_carlson                 -0.04  0.91 -0.02 0.84 0.161 1.0
## covid_accuracy_limbaugh                -0.01  0.93 -0.02 0.88 0.121 1.0
## covid_accuracy_nytimes                  0.65 -0.16  0.29 0.77 0.233 1.5
## covid_accuracy_cnn                      0.71 -0.12  0.21 0.75 0.254 1.2
## covid_accuracy_healthproviders         -0.01  0.08  0.82 0.65 0.351 1.0
## covid_accuracy_stategov                 0.24  0.12  0.45 0.36 0.636 1.7
## covid_accuracy_wallstreetjournal        0.49  0.15  0.35 0.53 0.474 2.0
## covid_accuracy_usatoday                 0.63  0.17  0.30 0.67 0.331 1.6
## covid_accuracy_medicalscientists        0.04 -0.10  0.80 0.72 0.283 1.0
## covid_accuracy_conservativejournalists  0.08  0.89  0.11 0.76 0.235 1.0
## covid_accuracy_centristjournalists      0.61  0.19  0.13 0.48 0.524 1.3
## covid_accuracy_youngturks               0.73  0.13 -0.08 0.49 0.514 1.1
## covid_accuracy_hayes                    0.94  0.10 -0.14 0.76 0.241 1.1
## covid_accuracy_maddow                   0.87 -0.18  0.02 0.85 0.154 1.1
## covid_accuracy_odonnell                 0.93  0.14 -0.10 0.78 0.220 1.1
##
##                        MR1  MR2  MR3
## SS loadings           6.83 5.98 3.68
## Proportion Var        0.30 0.26 0.16
## Cumulative Var        0.30 0.56 0.72
## Proportion Explained  0.41 0.36 0.22
## Cumulative Proportion 0.41 0.78 1.00
##
##  With factor correlations of
##       MR1   MR2   MR3
## MR1  1.00 -0.08  0.54
## MR2 -0.08  1.00 -0.21
## MR3  0.54 -0.21  1.00
```

Table S1. Output of factor analysis on trust items.

2. Party-specific relationships between COVID-19 precautions and ideological measures

Given our primary hypotheses regarding putative relationships between socially conservative attitudes and pathogen avoidance, in the main text we focus on the relationships between COVID-19 precautions and socially conservative attitudes. However, we also measured additional dimensions of political ideology, including economic and militaristic conservatism, as well as social dominance orientation and the other two subscales of right-wing authoritarianism,

submission to authority, and endorsement of authoritarian aggression. Here, we visualize the zero-order correlations between those additional dimensions of political belief and precautionary COVID-19 behaviors in Studies 1 and 2. Of particular interest, economic conservatism negatively correlated with precautions among Republicans and Independents, yet positively correlated with precautions among Democrats. Further, social dominance orientation negatively associated with precaution among Republicans and Independents in Study 1, and among supporters of all three affiliations in Study 2.

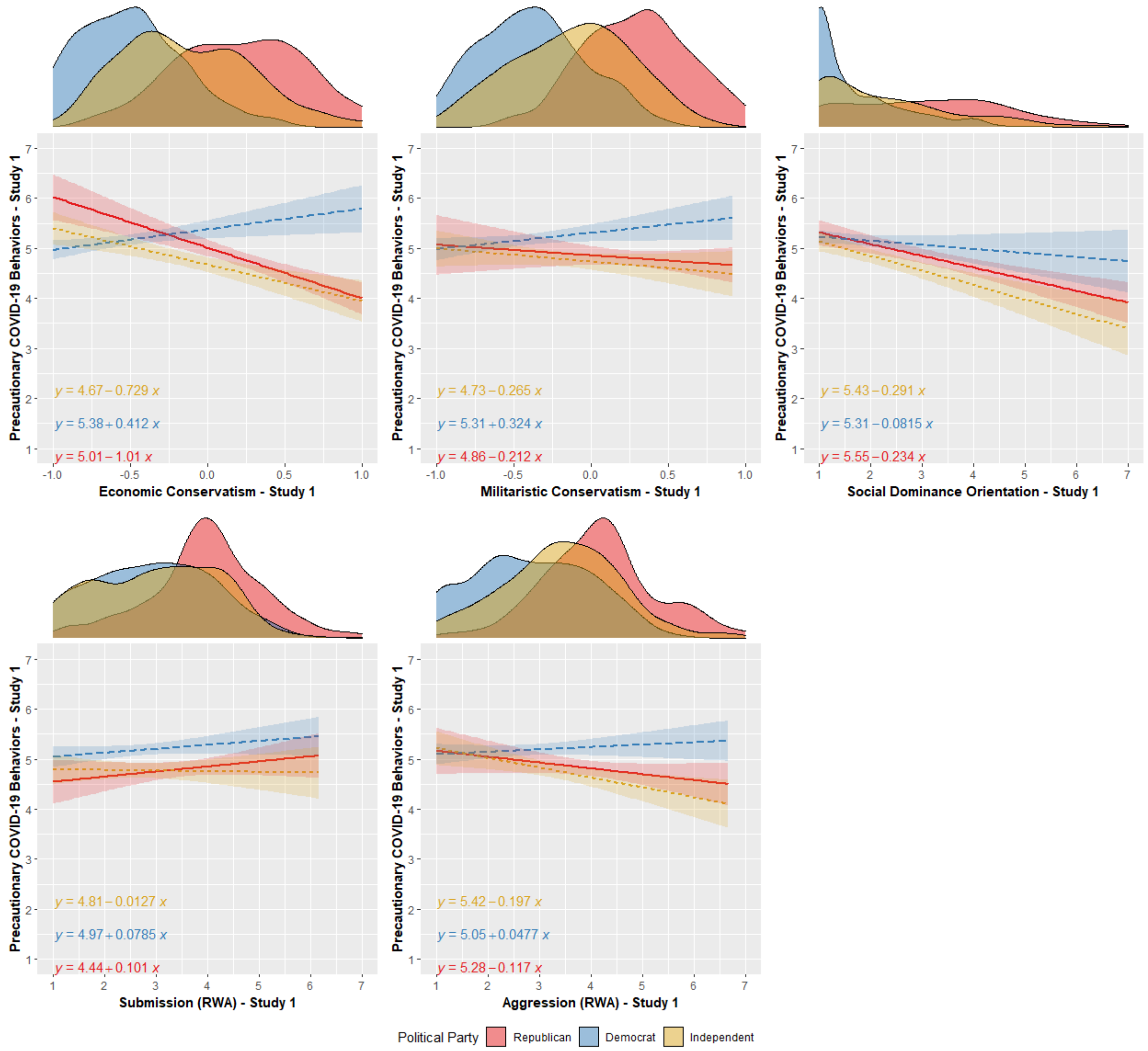

Figure S6. Study 1 conditional effects of moderated linear regressions in which COVID-19 precautions were separately regressed on each individual (centered) political ideology measure, political party affiliation, and the two-way interaction between party affiliation and the particular ideology measure. Bands around regression lines are 95% confidence intervals. The density plots along the x-axes represent the raw distributions of the ideology measures by political affiliation. On each plot, regression equations indicate the conditional simple slopes relationships between

ideological measures and COVID-19 precautions by political affiliation (slopes are unstandardized).

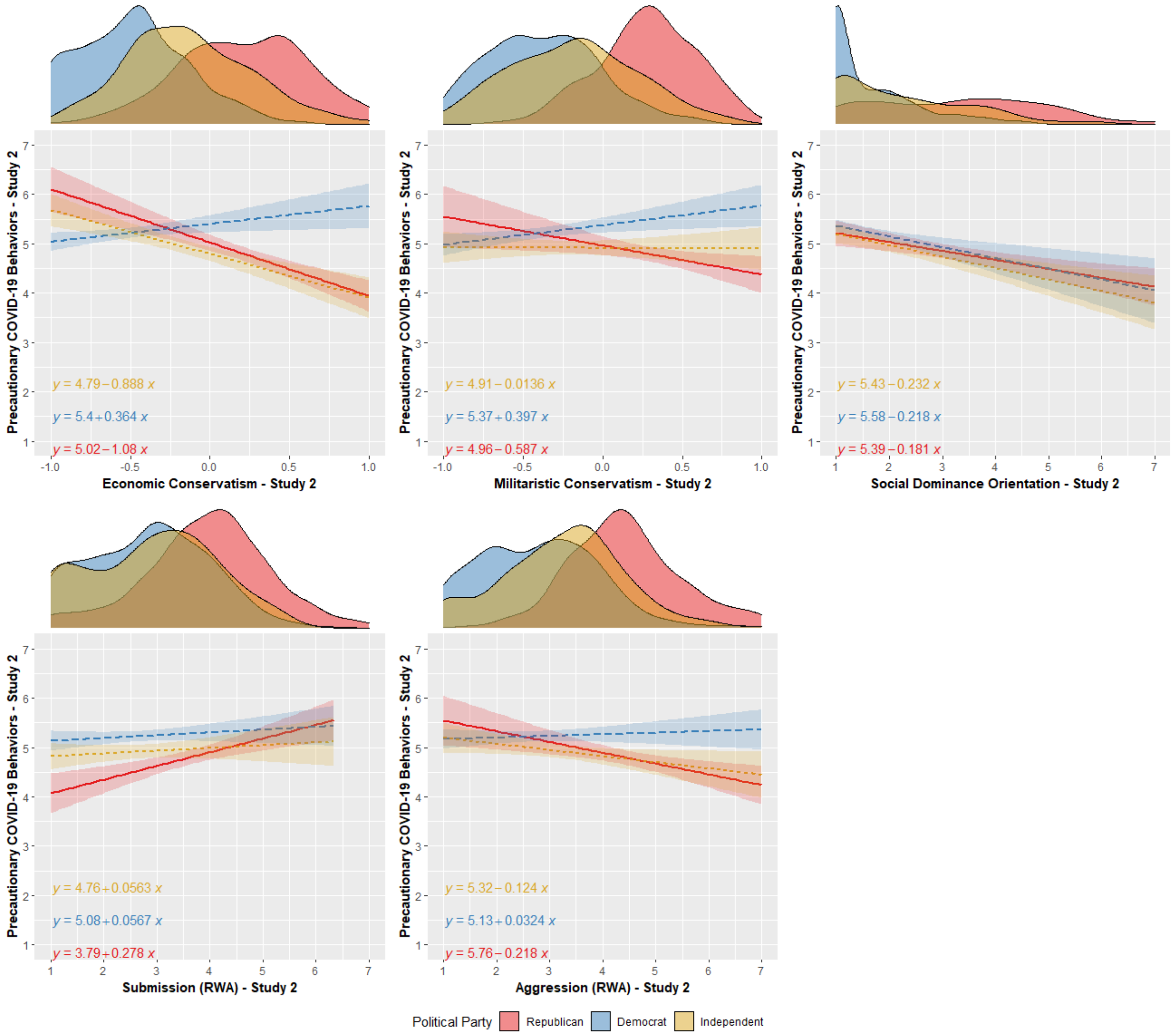

Figure S7. Study 2 conditional effects of moderated linear regressions in which COVID-19 precautions were separately regressed on each individual (centered) political ideology measure, political party affiliation, and the two-way interaction between party affiliation and the particular ideology measure. Bands around regression lines are 95% confidence intervals. The density plots along the x-axes represent the raw distributions of the ideology measures by political affiliation. On each plot, regression equations indicate the conditional relationships between ideological measures and COVID-19 precautions by political affiliation.

3. Testing for suppressors of the precautions-socially conservative attitudes relationship

In Study 1, we considered a large number of potential suppressor variables (see Table S2 for list of variables) that may shape partisan differences in precautionary COVID-19 behaviors. In the main text, we indicate that in Study 1 we then conducted an exploratory, bottom-up analysis to identify whether any of those theoretically-motivated variables were suppressing the relationship between socially conservative attitudes and precautionary behaviors among Republicans. To identify possible suppressors, we tested whether each target variable inconsistently mediated the relationship between socially conservative attitudes and precautionary behaviors among Republicans. That is, in order to be considered a suppressor, the target variable has to result in a significant and negative indirect effect between socially conservative attitudes and precautionary behaviors. The table below reports the results of exhaustive testing of the candidate Study 1 variables for possible suppression. Because Study 2 was a confirmatory replication, the exploratory suppressor-identification process was not used, and instead suppression was tested for using only the variables previously identified in Study 1 (see Main Text for results of suppressor models in Study 2).

**Suppressor**	**Bootstrapped unstandardized indirect effect**	**Lower unstandardized bootstrapped 95% CI**	**Upper unstandardized bootstrapped 95% CI**	**Bootstrapped standardized indirect effect**	**Lower standardized bootstrapped 95% CI**	**Upper standardized bootstrapped 95% CI**
Economic conservatism composite	-0.13	-0.20	-0.08	-0.15	-0.21	-0.09
Militaristic conservatism composite	-0.01	-0.05	0.02	-0.02	-0.06	0.02
Social dominance orientation composite	-0.04	-0.09	0.00	-0.04	-0.09	0.00
Submission to authority composite	0.01	-0.01	0.05	0.02	-0.01	0.05
Endorsement of authoritarian aggression composite	-0.01	-0.04	0.00	-0.02	-0.05	0.01
Perceived effectiveness of handwashing	0.04	-0.02	0.10	0.05	-0.02	0.11
Perceived effectiveness of mask wearing	-0.05	-0.13	0.04	-0.06	-0.15	0.03
Perceived effectiveness of hydroxychloroquine	0.02	0.00	0.05	0.02	-0.01	0.05
Perceived effectiveness of social distancing	-0.02	-0.10	0.06	-0.02	-0.10	0.06

Perceived effectiveness of disinfecting surfaces	0.00	-0.08	0.09	0.00	-0.09	0.09
Perceived effectiveness of immune system supplements	0.02	-0.03	0.07	0.02	-0.03	0.08
Perceived effectiveness of silver solution	0.01	-0.02	0.04	0.01	-0.02	0.04
Perceived prevalence of COVID-19 in local community	-0.03	-0.09	0.03	-0.03	-0.09	0.03
Perceived population density of local community	-0.04	-0.09	0.00	-0.04	-0.09	0.00
Age	-0.02	-0.04	0.00	-0.02	-0.05	0.00
Trust in liberals and moderates composite	-0.06	-0.12	-0.02	-0.07	-0.12	-0.03
Trust in scientists composite	-0.06	-0.11	-0.01	-0.06	-0.12	-0.01
Trust in conservatives composite	0.03	-0.01	0.07	0.03	-0.01	0.08
Conservative media consumption	0.02	0.00	0.04	0.02	0.00	0.05
Liberal media consumption	-0.06	-0.10	-0.03	-0.06	-0.11	-0.03

Total news consumption across any source	0.00	-0.03	0.02	0.00	-0.03	0.02
Precautionary economic behaviors	0.03	-0.01	0.09	0.03	-0.02	0.09
Concern over medical care access because of COVID-19	0.00	-0.04	0.04	0.00	-0.04	0.04
COVID-19 domain-specific threat assessment composite	-0.04	-0.11	0.02	-0.05	-0.12	0.02
Engage in COVID19 precautions out of concern for own health	-0.04	-0.10	0.00	-0.05	-0.10	0.00
Engage in COVID19 precautions out of concern for others' health	0.04	-0.01	0.10	0.05	-0.01	0.11
Perceived likelihood of contracting COVID-19 oneself	0.01	-0.04	0.06	0.01	-0.04	0.06
Perceived likelihood of family member contracting COVID-19	0.00	-0.04	0.03	0.00	-0.04	0.03

Perceived likelihood of someone in one's community contracting COVID-19	-0.02	-0.07	0.03	-0.02	-0.08	0.02
Perceived severity of economic consequences one faces	-0.01	-0.04	0.01	-0.02	-0.05	0.01
Perceived effectiveness of president's response to COVID-19	0.01	-0.02	0.05	0.01	-0.02	0.05
Perceived effectiveness of congress' response to COVID-19	0.00	-0.03	0.03	0.00	-0.04	0.03
Perceived effectiveness of one's state and local governments' response to COVID-19	0.00	-0.03	0.04	0.00	-0.03	0.04
Income	0.00	-0.01	0.02	0.00	-0.01	0.03
Government public health intervention opinions composite	0.00	-0.04	0.03	0.00	-0.04	0.04

Table S2. Summary of suppression identification process. All candidate variables were tested for a negative and significant indirect effect between socially conservative attitudes and precautionary COVID-19 behaviors, which would indicate suppression. Four variables had a significant negative indirect effect: economic conservatism, trust in scientists, trust in liberals and moderates, and liberal news media consumption. Estimates of the indirect effects and 95% confidence intervals are taken from averages of 5,000 bootstrapped iterations.

Based on the table above, four variables had significant suppressive effects: economic conservatism, the trust in scientists composite, the trust in liberals and moderates composite, and the liberal media composite. Economic conservatism was the strongest suppressor.

In Figure 2 in the Main Text, we then assessed the party-specific relationships between the four suppressor variables and COVID-19 precautions, in order to understand how these suppressor variables resulted in significant indirect effects between socially conservative attitudes and precautions. Additionally, we aimed to test the possibility that these identified suppressors may interact with political party affiliation in correlating with COVID-19 precautions. For example, economic conservatism positively associated with precautions among Democrats, but was negatively correlated with precautions among Republicans and Independents (see Figure S6). If the effects of the suppressors on precautionary behaviors was dependent on party affiliation, then it would be important to include those interactions in the joint suppression model.

In the Main Text, we report that political party was a significant moderator of all four suppressor variables in their relationships with COVID-19 precautions. The statistics for these interactions are reported in Table S3. Further, in the Main Text, we used the results of simple slopes analyses individually regressing COVID-19 precautions on the four suppressor variables and their interactions with political party to claim that greater trust in scientists, trust in liberals and moderates, and liberal media consumption were all positively correlated with COVID-19 precautions among Republicans and Independents in both studies. Further, economic conservatism was negatively associated with COVID-19 precautions among Republicans and Independents in both studies. Among Democrats, there was a positive correlation in Study 2 but not Study 1 between trust in scientists and precautions, no correlation between precautions and

trust in liberals and moderates in either study, and a positive correlation between precautions and both liberal media consumption and economic conservatism in both studies. The statistics for these simple slopes are reported in Table S4.

	**PRECAUTIONARY COVID-19 BEHAVIORS, STUDY 1**				**PRECAUTIONARY COVID-19 BEHAVIORS STUDY 2**			
Interaction effects	*B*	*SE*	*t value*	*p*	*B*	*SE*	*t value*	*p*
Trust in scientists, Democrats-Republicans	.28	.08	3.62	<.001	.25	.08	3.24	.001
Trust in scientists, Democrats-Independents	.14	.07	1.85	.065	.15	.08	1.99	.047
Trust in scientists, Republicans-Independents	-.14	.08	-1.81	.071	-.10	.07	-1.36	.175
Trust in liberal and moderate sources, Democrats-Republicans	.23	.07	3.53	<.001	.33	.06	5.19	< .001
Trust in liberal and moderate sources, Democrats-Independents	.12	.07	1.77	.077	.15	.06	2.58	.010
Trust in liberal and moderate sources, Republicans-Independents	-.12	.07	-1.67	.095	-.18	.07	-2.68	.008
Liberal media consumption, Democrats-Republicans	.17	.09	1.92	.056	.25	.08	3.25	.001
Liberal media consumption,	.18	.09	2.04	.042	.23	.09	2.63	.009

Democrats-Independents								
Liberal media consumption, Republicans-Independents	.01	.11	.10	.924	-.02	.10	-.23	.820
Economic conservatism, Democrats-Republicans	-1.42	.24	-5.89	<.001	-1.44	.24	-6.07	<.001
Economic conservatism, Democrats-Independents	-1.14	.23	-4.87	<.001	-1.25	.23	-5.39	<.001
Economic conservatism, Republicans-Independents	.28	.25	1.13	.261	.19	.25	.76	.448

Table S3. COVID-19 precautions were individually regressed on the interaction between each of the four suppressor variables and political party affiliation. This table summarizes the statistics of the interaction between each political party affiliation comparison, and each of the four suppressor variables. Political party affiliation significantly moderated the effect of each of the suppressor variables on COVID-19 precautions in both studies.

	**PRECAUTIONARY COVID-19 BEHAVIORS, STUDY 1**				**PRECAUTIONARY COVID-19 BEHAVIORS STUDY 2**			
Simple slopes	*B*	*SE*	*t value*	*p*	*B*	*SE*	*t value*	*p*
Trust in scientists—Democrats	.04	.05	.78	.437	.17	.06	2.86	.004
Trust in scientists—Republicans	.32	.05	5.79	<.001	.42	.05	8.21	<.001
Trust in scientists—Independents	.18	.05	3.39	.001	.32	.05	6.34	<.001
Trust in liberals and moderates—Democrats	.02	.04	.47	.635	.01	.04	.30	.767
Trust in liberals and moderates —Republicans	.26	.05	5.09	<.001	.34	.05	6.87	<.001
Trust in liberals and moderates —Independents	.14	.05	2.77	.006	.17	.04	.3.74	<.001
Liberal media consumption—Democrats	.21	.05	4.38	<.001	.19	.05	4.10	<.001
Liberal media consumption —Republicans	.38	.08	5.04	<.001	.44	.06	7.04	<.001
Liberal media consumption —Independents	.39	.07	5.21	<.001	.42	.08	5.62	<.001

Economic conservatism— Democrats	.41	.16	2.61	.009	.36	.15	2.39	.017
Economic conservatism — Republicans	-1.01	.18	-5.53	<.001	-1.08	.18	-5.91	<.001
Economic conservatism — Independents	-1.01	.18	-4.22	<.001	-.89	.18	-5.05	<.001

Table S4. COVID-19 precautions were individually regressed on the interaction between each of the four suppressor variables and political party affiliation. This table reports the simple slopes relationships between each of the suppressor variables and COVID-19 precautions for each level of political party affiliation.

Finally, in order to understand how each suppressor variable resulted in a negative indirect effect between socially conservative attitudes and COVID-19 precautions, we individually regressed each suppressor variable on socially conservative attitudes and its interaction with political party. In the Main Text, of the four suppressor variables, we claimed that socially conservative attitudes negatively correlated with trust in scientists, and trust in liberals and moderates among Republicans and Independents in both studies, but positively correlated with economic conservatism. Further, socially conservative attitudes negatively correlated with liberal media consumption among Republicans in both studies, but among Independents, this significant correlation only obtained in Study 2. We plot the conditional effects of socially conservative attitudes by political party on each suppressor variable in Figure S8, and report the results of simple slopes analyses in Table S5.

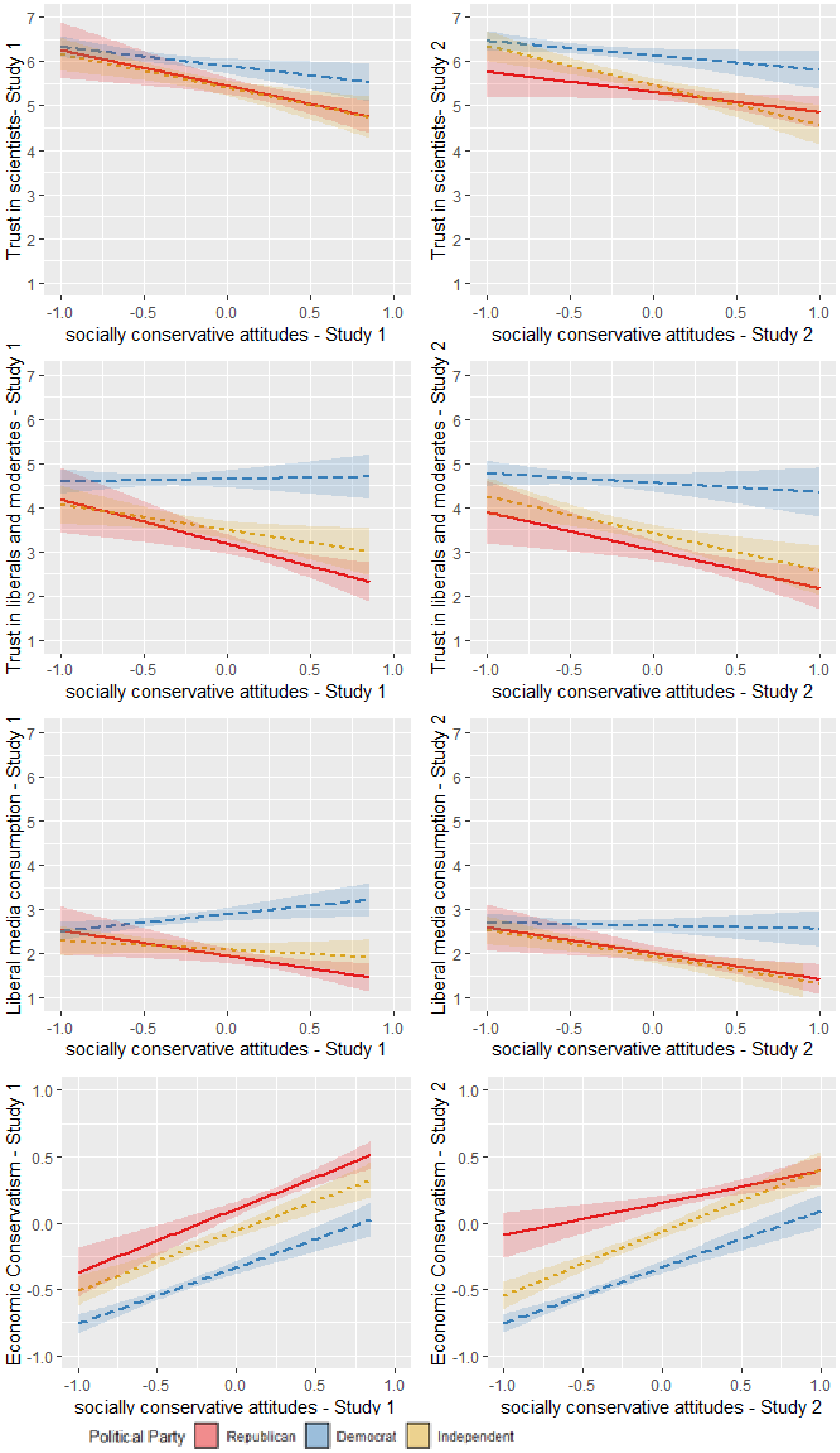
Trust in scientists- Study 1
socially conservative attitudes - Study 1
Trust in scientists- Study 2
socially conservative attitudes - Study 2
Trust in liberals and moderates - Study 1
socially conservative attitudes - Study 1
Trust in liberals and moderates - Study 2
socially conservative attitudes - Study 2
Liberal media consumption - Study 1
socially conservative attitudes - Study 1
Liberal media consumption - Study 2
socially conservative attitudes - Study 2
Economic Conservatism - Study 1
socially conservative attitudes - Study 1
Economic Conservatism - Study 2
socially conservative attitudes - Study 2
Political Party
Republican
Democrat
Independent

Figure S8. Studies 1 and 2 condition effects of moderated linear regressions in which each of the four suppressor variables were separately regressed on socially conservative attitudes, political party affiliation, and their two-way interaction. Bands around regression lines are 95% confidence intervals.

	**TRUST IN SCIENTISTS, STUDY 1**				**TRUST IN SCIENTISTS, STUDY 2**			
Simple slopes	*B*	*SE*	*t value*	*p*	*B*	*SE*	*t value*	*p*
Socially conservative attitudes— Democrats	-.43	.17	-2.51	.012	-.33	.16	-2.12	.035
Socially conservative attitudes — Republicans	-.81	.26	-3.11	.002	-.45	.22	-2.03	.042
Socially conservative attitudes — Independents	-.77	.21	-3.61	<.001	-.90	.19	-4.80	<.001
	**TRUST IN LIBERAL AND MODERATE SOURCES, STUDY 1**				**TRUST IN LIBERAL AND MODERATE SOURCES , STUDY 2**			
Socially conservative attitudes— Democrats	.06	.20	.29	.770	-.22	.20	-1.07	.284
Socially conservative attitudes — Republicans	-1.00	.31	-3.28	.001	-.86	.28	-3.05	.002
Socially conservative attitudes — Independents	.-57	.25	-2.32	.021	-.84	.23	-3.59	<.001
	**LIBERAL MEDIA CONSUMPTION, STUDY 1**				**LIBERAL MEDIA CONSUMPTION , STUDY 2**			

Socially conservative attitudes—Democrats	.39	.15	2.55	.011	-.07	.15	-.48	.631
Socially conservative attitudes —Republicans	-.57	.23	-2.54	.011	-.59	.21	-2.80	.005
Socially conservative attitudes —Independents	-.20	.19	-1.08	.282	-.60	.17	-3.48	.001
	**ECONOMIC CONSERVATISM, STUDY 1**				**ECONOMIC CONSERVATISM, STUDY 2**			
Socially conservative attitudes—Democrats	.42	.05	8.38	<.001	.42	.05	9.02	<.001
Socially conservative attitudes —Republicans	.48	.08	6.37	<.001	.25	.07	3.65	<.001
Socially conservative attitudes —Independents	.45	.06	7.36	<.001	.48	.06	8.62	<.001

Table S5. Each suppressor variable was individually regressed on the interaction between socially conservative attitudes and political party affiliation. This table reports the simple slopes relationships between socially conservative attitudes and each of the suppressor variables for each level of political party affiliation.

4. Correlations between political measures

In the main text, we note that the political ideology and attitude measures were all highly correlated with each other. The following figures display those relationships for Studies 1 and 2.

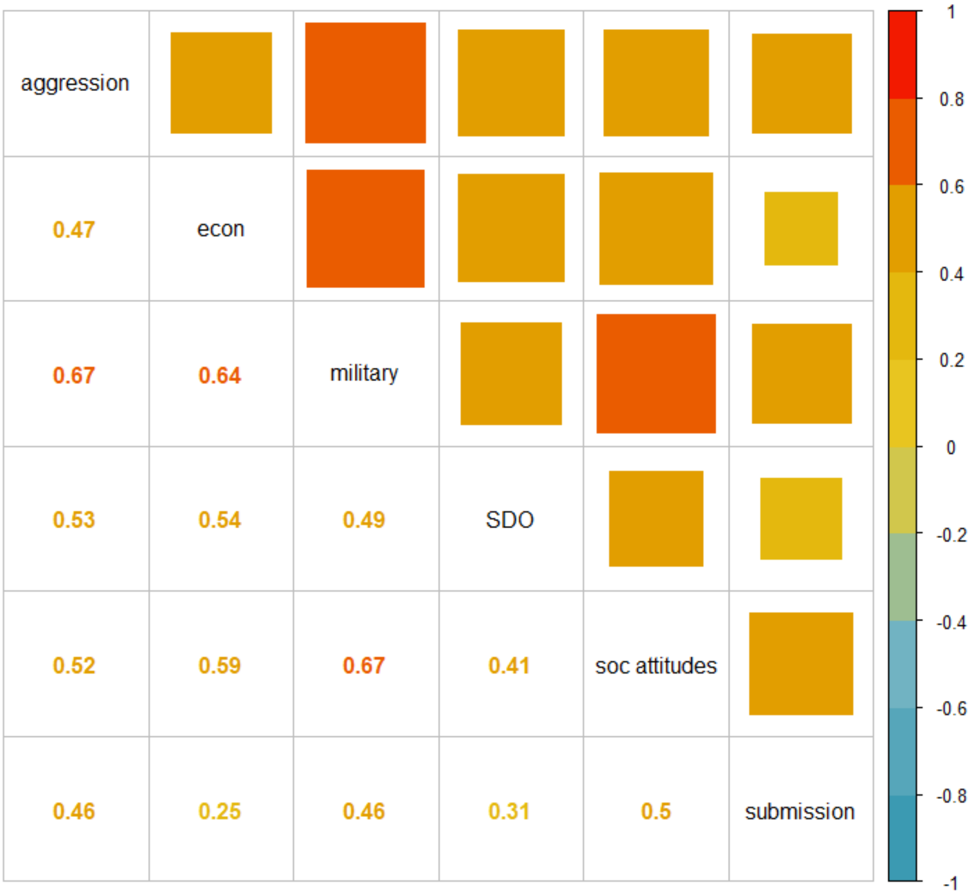

Figure S9. Correlations between political measures in Study 1. In this correlation matrix, square size and square color in the upper right triangle corresponds with the size of the correlation, displayed numerically in the lower left triangle.

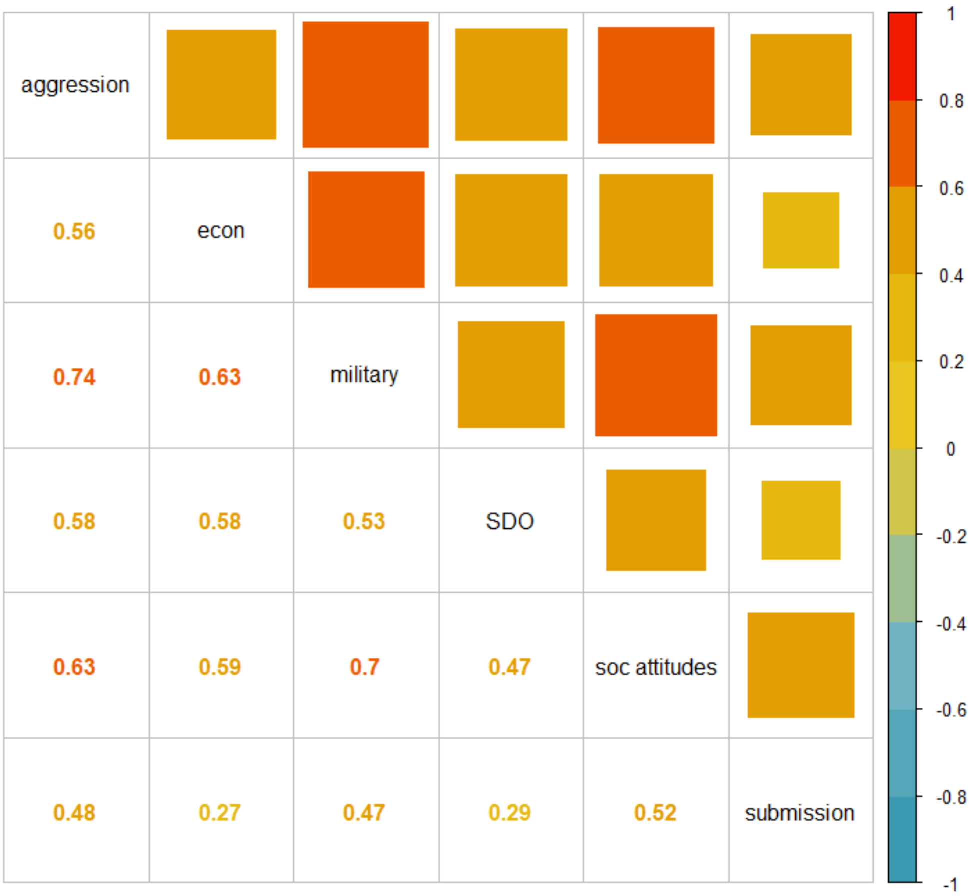

Figure S10. Correlations between political measures in Study 2. In this correlation matrix, square size and square color in the upper right triangle corresponds with the size of the correlation, displayed numerically in the lower left triangle.

5. Robustness of results after including covariates

In the main text, we claimed that the party-specific relationships--after accounting for the effects of the suppressor variables--between COVID-19 precautions and socially conservative

attitudes were largely robust to the inclusion of basic demographic variables, COVID-19-related covariates (see Figure S9 for list of covariates), and pathogen disgust sensitivity. Here, we plot the party-specific relationships between COVID-19 precautions and socially conservative attitudes, and report results of a simple slopes analysis by party affiliation.

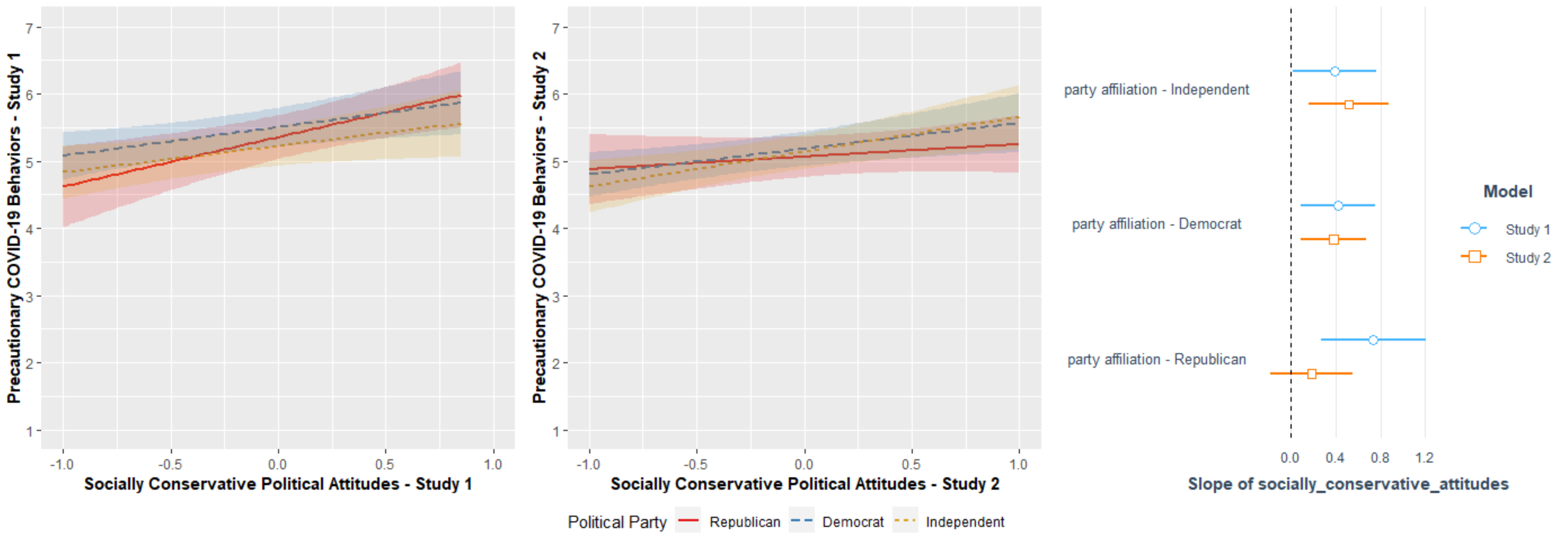

Figure S11. Studies 1 and 2 conditional effects of moderated linear regressions, in which a wide number of covariates were added to the suppression models specified in Figure 2 (see Main Text). These additional covariates were as follows: age, gender, ethnicity, income, education, pre-existing health conditions, self-reported density of local neighborhood, self-reported estimates of local COVID-19 prevalence, the extent to which one's job required leaving the household, and pathogen disgust sensitivity.

The left-two panes show the conditional relationships between socially conservative attitudes and COVID-19 precautions by political party, after accounting for both the effects of the previously-identified suppressor variables, as well as the additional covariates. Bands around regression lines are 95% confidence intervals.

The right-most pane plots the coefficients obtained from simple slopes analyses of the effect of socially conservative attitudes for each level of party affiliation across both studies. Coefficients are unstandardized. Lines indicate 95% confidence intervals. After accounting for the additional covariates, the positive correlations between socially conservative attitudes and COVID-19 precautions remained significant among Democrats and Independents in both studies. Among Republicans, the relationship only obtained in Study 1, and not Study 2.

6. Are the relationships between socially conservative attitudes and COVID-19 precautions attributable to other dimensions of political ideology?

In the Main Text, we claimed that including additional measures of political ideology—such as authoritarian aggression, submission to authority, social dominance orientation, economic conservatism, and militaristic conservatism—largely did not account for the party-specific correlations between socially conservative attitudes and precautionary COVID-19 behaviors. Further, we also claimed that socially conservative attitudes were the strongest positive ideological correlates of COVID-19 precautions among supporters of all three major party affiliations in Study 1, and among Democrats and Independents in Study 2. Below, we describe and present the results of the moderated linear regressions that were used to make these claims.

	**precautionary COVID-19 behaviors, Study 1**				**precautionary COVID-19 behaviors Study 2**			
Conditional effects	*B*	*SE*	*t value*	*p*	*B*	*SE*	*t value*	*p*
Socially conservative attitudes - Democrats	0.96	0.21	4.66	**<0.001**	0.81	0.19	4.28	**<0.001**
Socially conservative attitudes - Republicans	0.66	0.25	2.61	**0.009**	0.24	0.21	1.16	.246
Socially conservative attitudes - Independents	0.73	0.23	3.21	**0.001**	0.71	0.23	3.14	**0.002**

SDO - Democrats	-0.15	0.07	-2.27	**0.024**	-.30	0.06	-4.68	**<0.001**
SDO - Republicans	-0.16	0.06	-2.94	**0.003**	-.01	0.5	-.20	.841
SDO - Independents	-0.17	0.07	-2.59	**0.010**	-.03	.07	-.37	.709
Authoritarian Aggression - Democrats	-0.04	0.07	-.68	0.495	-.07	0.06	-1.12	.261
Authoritarian Aggression - Republicans	-.01	0.08	-.08	0.938	-.09	0.08	-1.14	.256
Authoritarian Aggression - Independents	-.16	0.09	-1.74	0.083	-.14	0.08	-1.82	.070
Submission to authority - Democrats	-.05	0.06	-.74	.461	0.04	0.06	.634	.526
Submission to authority - Republicans	-.04	0.09	-.49	.628	0.16	0.07	2.30	**.022**
Submission to authority - Independents	-.04	0.08	-.46	.649	-.08	0.08	-1.09	.277
Economic conservatism - Democrats	0.34	0.18	1.88	.061	0.32	0.17	1.93	.054
Economic conservatism - Republicans	-.52	0.25	-2.11	**.035**	-.25	0.22	-1.16	.247

Economic conservatism - Independents	-.58	0.23	-2.59	**.010**	-.89	0.23	-3.85	**<0.001**
Militaristic conservatism - Democrats	-.14	0.22	-.66	.513	0.09	0.20	.45	.656
Militaristic conservatism - Republicans	.34	0.27	1.27	.205	0.11	0.27	.39	.694
Militaristic conservatism - Independents	0.35	0.28	4.28	.202	0.57	0.23	2.47	**.014**
Observations	806				800			
R^2 / R^2 adjusted	0.23 / 0.20				0.30 / 0.28			

Table S6. Studies 1 and 2 regression table for moderated linear regressions examing the relationship between COVID-19 precautions and various ideological measures, after accounting for the effects of the suppressor variables identified in the Main Text. In addition to socially conservative attitudes, the following ideological measures were added to the model: social dominance orientation, submission to authority, authoritarian aggression, economic conservatism, and militaristic conservatism. Further, because we found that these ideological measures interacted with political party at baseline (see figures S6 and S7), they interacted with political party affiliation in these models as well.

Note that this table displays the conditional effects (based on simple slopes anlayses) of the ideological variables at each level of the moderator variable (political party affiliation). The intercept, interaction terms, and suppressor variables are not included in this table.

7. Relationships between socially conservative attitudes and pathogen disgust sensitivity

In the Main Text, we claimed that pathogen disgust sensitivity positively correlated with socially conservative attitudes among Democrats in both studies, among Independents in Study 2 only, and not among Republicans in either study. Below, we present the results that lead to that claim.

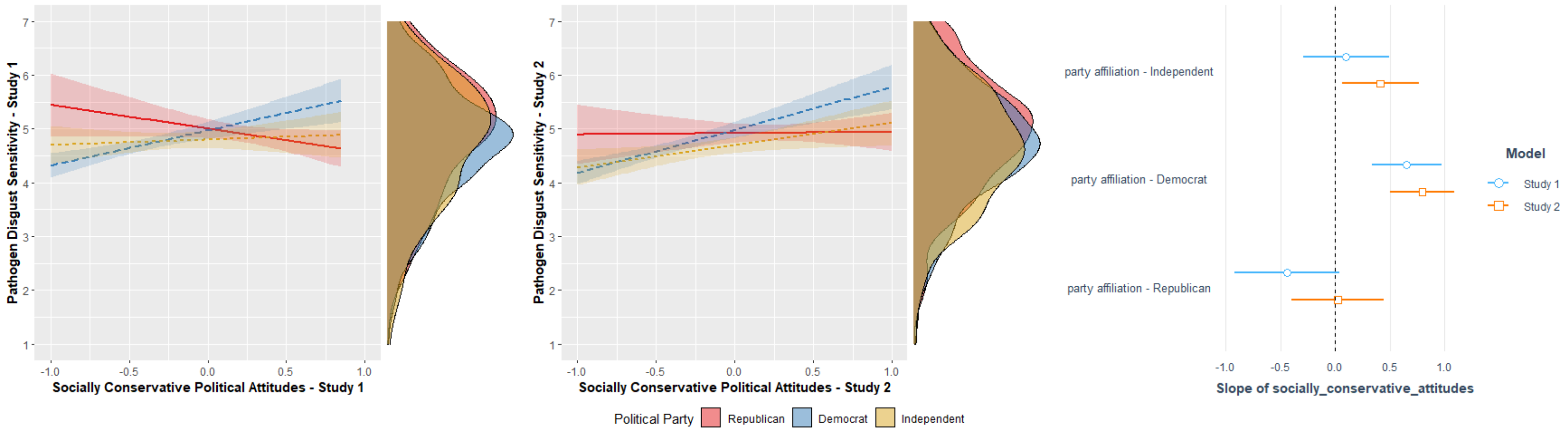

Figure S12. Studies 1 and 2 conditional effects of moderated linear regressions, in which the (centered) pathogen disgust sensitivity composite was regressed on socially conservative attitudes, political party affiliation, and their two-way interaction.

The left-two panes show the conditional relationships between socially conservative attitudes and disgust sensitivity by political party. Bands around regression lines are 95% confidence intervals. The density plots along the y-axes represent the raw distributions of pathogen disgust sensitivity by political party.

The right-most pane plots the coefficients obtained from simple slopes analyses for each level of party affiliation across both studies. Coefficients are unstandardized. Lines indicate 95% confidence intervals.

8. Determining whether liberal media consumption, trust in scientists, trust in liberals and moderates, and economic conservatism suppress the relationship between socially conservative attitudes and disgust sensitivity among Republicans and Independents

In the main text we report that although we found a positive correlation between socially conservative attitudes and either COVID-19 precautions or pathogen disgust sensitivity among Democrats, those relationships were not consistently found among Republicans or Independents. There are conceptual similarities in these results; a lack of a relationship between socially conservative attitudes and two different measures of pathogen avoidance among supporters of certain political affiliations, but not others. Therefore, we tested whether those variables that suppressed the precautions-socially conservative attitudes among Republicans and Independents–economic conservatism, the trust in scientists composite, and the trust in liberals and moderates composite–also suppressed the relationship between socially conservative attitudes and self-reported pathogen disgust sensitivity.

We did not find evidence for that possibility. In both studies, the joint effects of those three variables did not result in a significant and negative indirect pathway between socially conservative attitudes and precautionary behaviors among Republicans (bootstrapped unstandardized indirect effect = Study 1: -.07, 95% CI [-.28, .12]; Study 2: -.08, 95% CI [-.24, .05]). Among Independents, there was evidence for weak suppression in Study 1 (bootstrapped unstandardized indirect effect = -.24, 95% CI [-.45, -.04), but no significant suppression in Study 2 (bootstrapped unstandardized indirect effect = -.14, 95% CI [-.37, .07]). Although there was a significant suppressive effect among Independents in Study 1, this result did not replicate. Thus, we found minimal evidence for the possibility that the same set of variables are suppressing both

the precautions-disgust relationship and the precautions-socially conservative attitudes relationship.

9. Relationship between economic conservatism and COVID-19 threat concerns among Republicans

In the main text, we note that among Republicans, economic conservatism positively correlated with weighing the direct health threats of the pandemic as less serious relative to downstream economic and personal liberty threats. We tested whether economic conservatism positively associated with the COVID-19 threats assessment composite scale, where higher scores indicate weighting direct health threats as less serious. Indeed, in both studies, among supporters of all three parties, economic conservatism positively associated with agreement that the direct health threats posed by COVID-19 were less severe, particularly in contrast to the downstream economic and personal liberty threats.

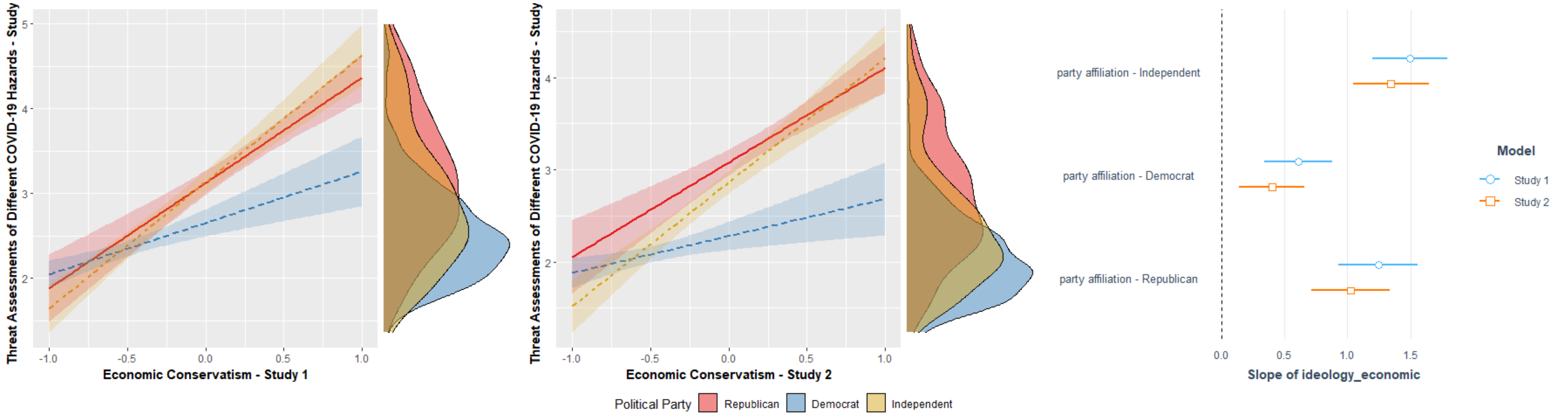

Figure S13. Studies 1 and 2 conditional effects of moderated linear regressions, in which threat assessments of different COVID-19 hazards was regressed on the (centered) economic conservatism composite, political party affiliation, and their two-way interaction.

The left-two panes show the conditional relationships between the threat assessments composite and economic conservatism by political party. Bands around regression lines are 95% confidence intervals. The density plots along the y-axes represent the raw distributions of the threat assessments composite by political party. Higher scores along the threat assessments composite on the y-axis indicate finding the direct health threats of the pandemic as less serious, especially compared to economic or personal liberty threats.

The right-most pane plots the coefficients obtained from simple slopes analyses for each level of party affiliation across both studies. Coefficients are unstandardized. Lines indicate 95% confidence intervals.

10. Political affiliation differences in economic precautions

In the main text, we note that Republicans were no more likely to report taking personal steps to buffer themselves against the potential economic consequences of the pandemic than were Democrats or Independents. Here, we show that there are no significant differences among the three partisan categories in the economic precautions composite, which included items such as asking how focused participants were on delaying major financial decisions, or preparing for a major economic downturn.

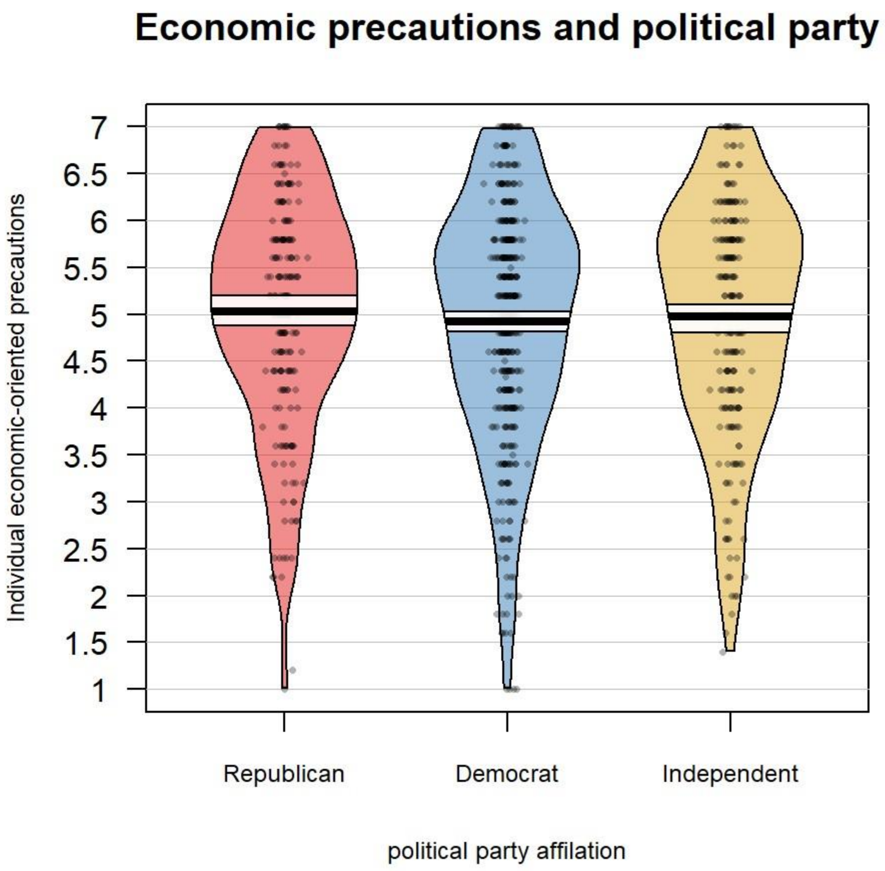

Figure S14. Pirate plot comparing differences in self-reported economic precautions by political party, Study 1. Scatterplot points are raw data, jittered to reduce overlap. Beans show smoothed density of data points. Bars and boxes represent means and Bayesian 95% highest density intervals, respectively.

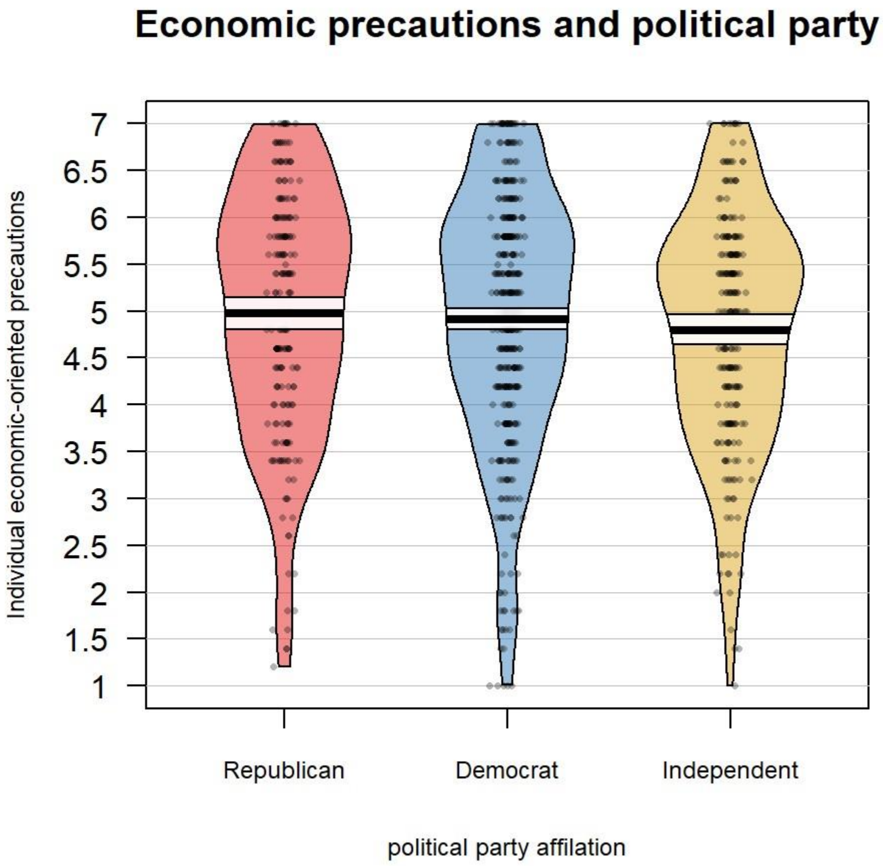

Figure S15. Pirate plot comparing differences in self-reported economic precautions by political party, Study 2. Scatterplot points are raw data, jittered to reduce overlap. Beans show smoothed density of data points. Bars and boxes represent means and Bayesian 95% highest density intervals, respectively.

11. Perceived health threat of COVID-19 and suppressor variables

In the Discussion section of the Main Text, we claim that the four suppressor variables clashed with the perception that COVID-19 poses a substantial health threat, particularly relative to threats posed to the economy and personal liberties. Here, we demonstrate those relationships. We individually regressed the COVID-19 threat-assessments composite (where

higher scores indicate perceiving the direct health threat posed by the pandemic to be less concerning and threatening, but the economic and personal liberty threats as more worrisome, and lower scores the reverse) on each of the four suppressor variables and their interactions with political party affiliation. We find that in among Republicans and Independents, in both studies, lower concern about the direct health threat of the pandemic is associated with lower trust in scientists, lower consumption of liberal news media, lower trust in liberal and moderate information sources, and higher economic conservatism, suggesting that the suppressor variables indeed clash with the perception that COVID-19 poses a serious health threat. Among Democrats, these effects obtain in both studies for trust in scientists and economic conservatism; trust in liberals and moderates in Study 1 but not Study 2; and in neither study for liberal media consumption.

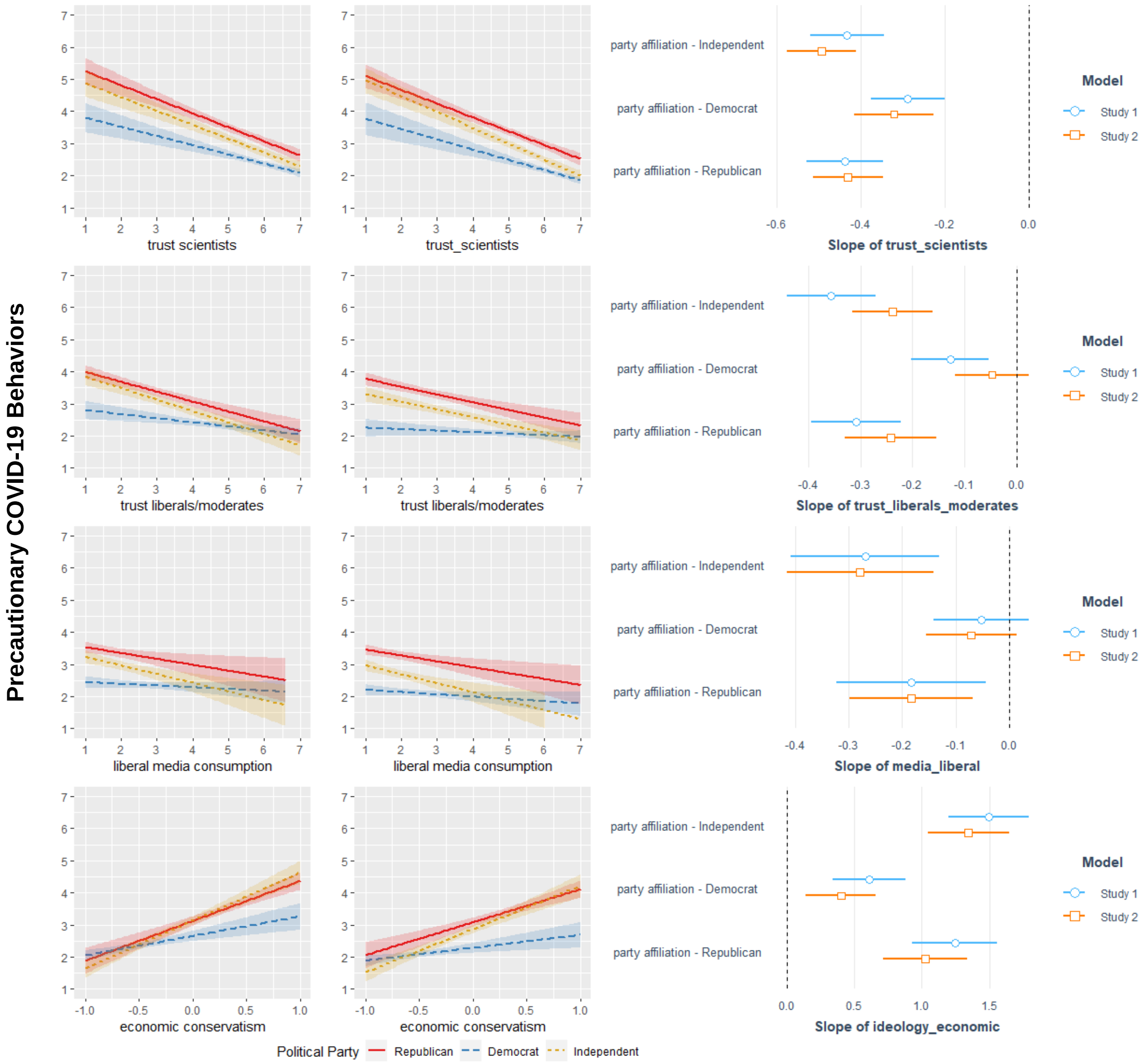

Figure S16. Studies 1 and 2 conditional effects of moderated linear regressions, in which threat assessments of COVID-19 hazards was individually regressed on the four (centered) suppressor variables, political party affiliation, and their two-way interactions.

The first two columns show the conditional relationships between the threat assessments composite and the suppressor variables by political party. Bands around regression lines are 95% confidence intervals. Higher scores along the threat assessments composite on the y-axis indicate finding the direct health threats of the pandemic as less serious, especially compared to economic or personal liberty threats.

The right-most column plots the coefficients obtained from simple slopes analyses for each level of party affiliation across both studies. Coefficients are unstandardized. Lines indicate 95% confidence intervals

References

Lüdecke, D., Ben-Shachar, M. S., & Makowski, D. (2020). Describe and understand your model's parameters. *CRAN*. https://doi.org/10.5281/zenodo.3731932
