# Supplementary material for: Of pathogens and party lines: Social conservatism positively associates with COVID-19 precautions among U.S. Democrats but not Republicans
Source: PLoS One. 2021 Jun 29;16(6):e0253326. doi: 10.1371/journal.pone.0253326 (PMC8241032; doi:10.1371/journal.pone.0253326)
Supplement: S4 Appendix — (PDF) [file pone.0253326.s004.pdf]

S4 APPENDIX – ADDITIONAL ANALYSES TO: Social Conservatism Positively Associates with COVID-19 Precaution among Democrats but not Republicans

Contents

1. Relationships between COVID-19 health precautions and trust composites 3

2. Relationships between COVID-19 health precautions and opinions regarding government public health interventions 11

3. Relationships between COVID-19 health precautions and demographic variables 13

4. Item-by-item analyses of political party differences in precautionary COVID-19 health behaviors 27

5. Relationship between economic conservatism and pathogen disgust sensitivity 32

6. Results without lockdown order item in COVID-19 health precautions composite 33

7. Results separating social conservatism and traditionalism measures 40

8. Effects of perceived closeness to preferred political party affiliation 49

9. Effects of time on the relationship between political party affiliation and COVID-19 precautions 50

References 53

1. Relationships between COVID-19 health precautions and trust composites

In the main text, we hypothesize that differences in trust for various sources of information may shape partisan differences in COVID-19 health precautions. We showed that two composite measures–trust in scientists and trust in liberal and moderate information sources–in part suppressed the relationship between socially conservative attitudes and health precautions among Republicans and Independents, that is, the otherwise positive relationship between social conservatism and prophylaxis is masked by low trust in scientists and low trust in liberal and moderate information sources. However, it is also worth investigating the zero-order correlations between the three trust composites and health precautions by political party, in order to explore the partisan relationship between COVID-19 prophylaxis and trust more broadly. As illustrated in Figure S16, in both Study 1 and Study 2, trust in liberal and moderate information sources correlated with precautionary behaviors among Republicans and Independents, but not Democrats. The other trust-precautions relationships were not consistent across Studies 1 and 2. In Study 1, trust in scientists correlated with precautions among Republicans and Independents, but not Democrats; however, in Study 2, that correlation was significant in supporters of all three major party affiliations (although the effect was substantially smaller in Democrats relative to Republicans and Independents). Trust in conservative information sources positively correlated with precautions among Democrats in Study 1, but not Study 2, whereas the positive association obtained among Republicans in Study 2, but not Study 1. Because of the inconsistency of these latter effects, it would be erroneous to draw any specific and conclusive interpretations; nevertheless, in general, these results suggest that trust in scientists and trust in liberals and moderates positively associate with precautions in a party-specific manner.

Lastly, the density plots indicate that Democrats were more trusting of scientific and liberal and moderate sources, and less trusting of conservative sources, relative to Republicans. Independents were intermediate along these dimensions.

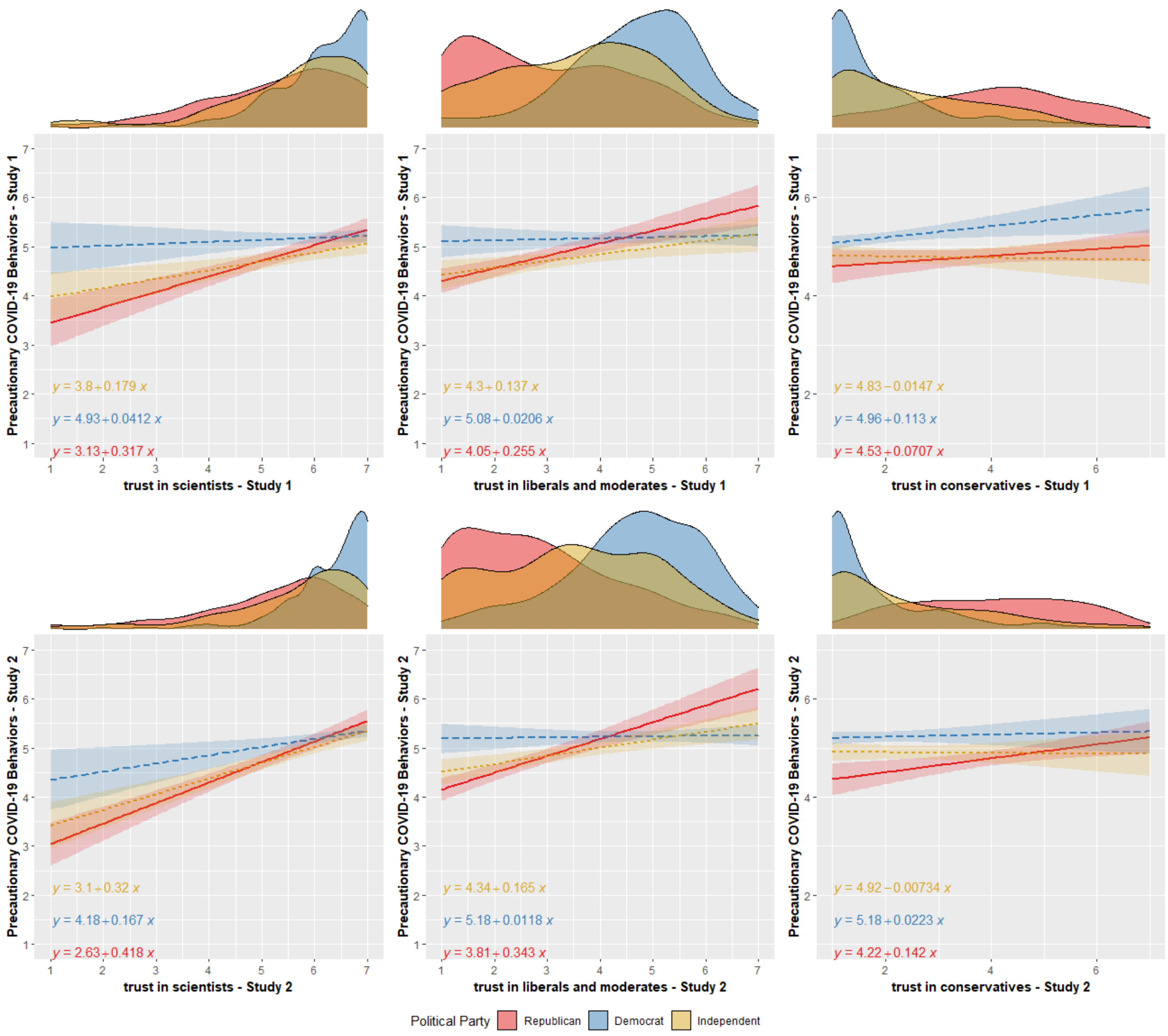

Figure S17. Studies 1 and 2 conditional effects of moderated linear regressions in which COVID-19 precautions were separately regressed on each individual (centered) composite trust measure, political party affiliation, and the two-way interaction between party affiliation and the particular trust composite. Bands around regression lines are 95% confidence intervals. The density plots along the x-axes represent the raw distributions of the trust composites by political affiliation. On each plot, regression equations indicate the conditional simple slopes relationships between trust composites and COVID-19 precautions by political affiliation (slopes are unstandardized).

Relationships between COVID-19 health precautions and media consumption composites

In the main text, we hypothesize that differences in media consumption for various sources of partisan information may shape political differences in COVID-19 health precautions. We found evidence that one of the partisan media consumption composites–consumption of liberal news media–in part suppressed the relationship between socially conservative attitudes and health precautions among Republicans and Independents. However, following the same logic as in the previous section, it is also worth investigating the zero-order correlations between the two composites (liberal media consumption, and conservative media consumption), and precautions by political party, in order to explore the partisan relationship between COVID-19 prophylaxis and media consumption more broadly.

Self-reported consumption of liberal-leaning media sources positively correlated with COVID-19 precautionary behaviors among Republicans, Democrats, and Independents, while self-reported consumption of conservative-leaning media sources positively correlated with precautions only among Democrats and Republicans, not Independents, see plot below. Unsurprisingly, the density plots indicate that Democrats consume more liberal news relative to non-Democrats, and that Republicans consume more conservative news relative to non-Republicans. Note, however, that even among Republicans, there were very low levels of self-reported conservative media consumption. This result could be due to the specific conservative media outlets that we chose to include in the survey, which may not be highly consumed by the conservatives we happened to recruit in our sample. For example, media figures that primarily operate on platforms such as YouTube and Twitter were not included in the survey, which may be more popular with participants recruited from MTurk. Conversely, the kinds of media outlets that conservatives in our sample may consume more frequently may not have been included in

our survey, since the list of media outlets was not exhaustive. Further, because participants were asked to rate their consumption of many different outlets, which were then averaged into a composite, the inclusion of possibly rarely consumed conservative media outlets may be disproportionally depressing that average relative to the outlets included in the liberal media composite. We further note that this extreme skew likely affects and limits the interpretability of any inferential analyses that include the conservative media consumption composite. Most notably, the fact that conservative media consumption did not suppress the relationship between socially conservative attitudes and precautionary COVID-19 behaviors in our suppression analyses, while liberal media consumption did, may be attributable to the lack of variation in the conservative media consumption composite, rather than a true null effect of conservative media consumption on that relationship.

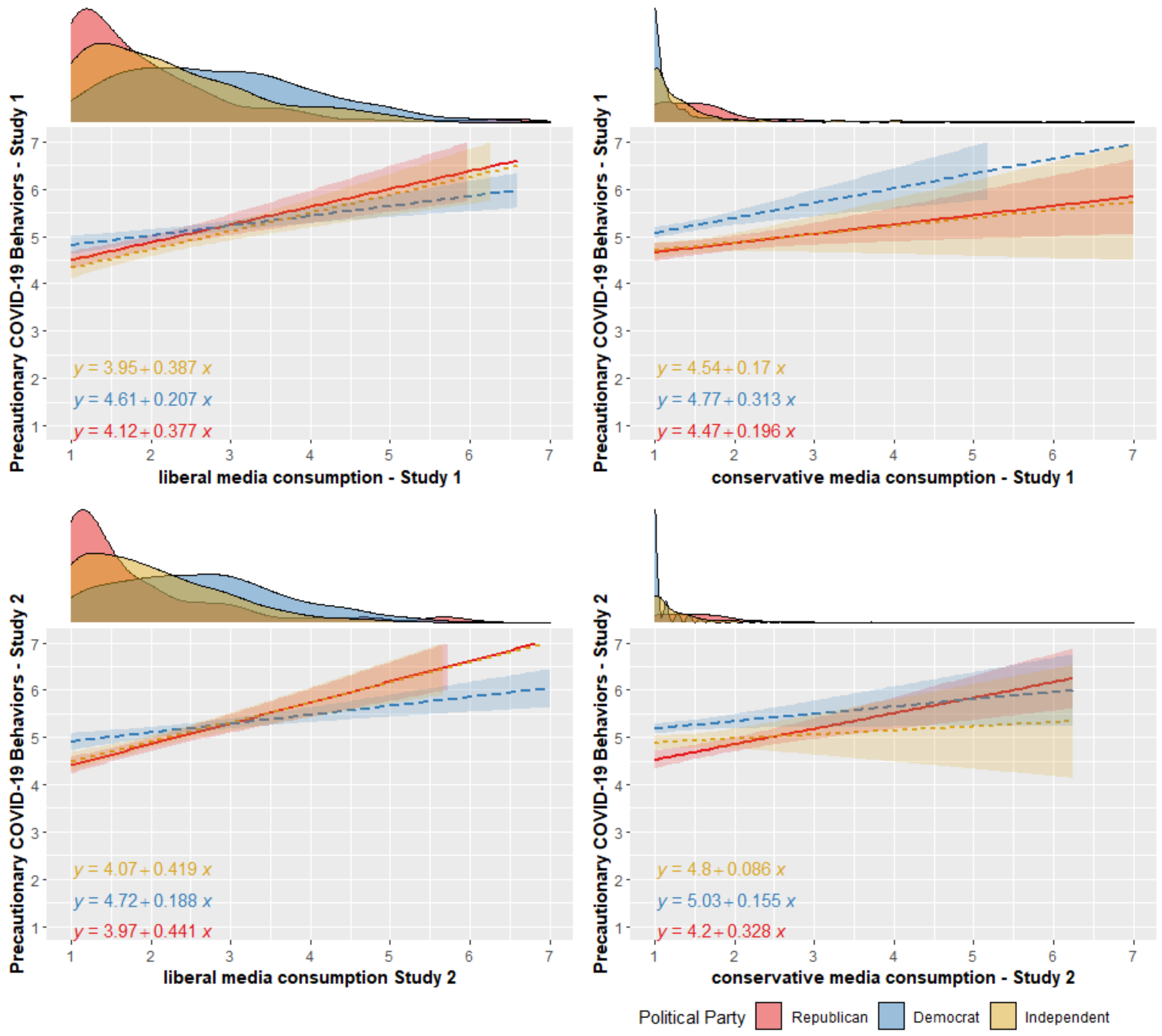

Figure S18. Studies 1 and 2 conditional effects of moderated linear regressions in which COVID-19 precautions were separately regressed on each individual (centered) composite media consumption measure, political party affiliation, and the two-way interaction between party affiliation and the particular media consumption composite. Bands around regression lines are 95% confidence intervals. The density plots along the x-axes represent the raw distributions of the media consumption composites by political affiliation. On each plot, regression equations indicate the conditional simple slopes relationships between the media consumption composites and COVID-19 precautions by political affiliation (slopes are unstandardized).

Additionally, total news consumption from any source (including sources that may not have been listed among the items that constituted the partisan news consumption composites)

positively correlated with precautions among Democrats and Republicans, but not Independents, in Study 1. In Study 2, those relationships only approached significance.

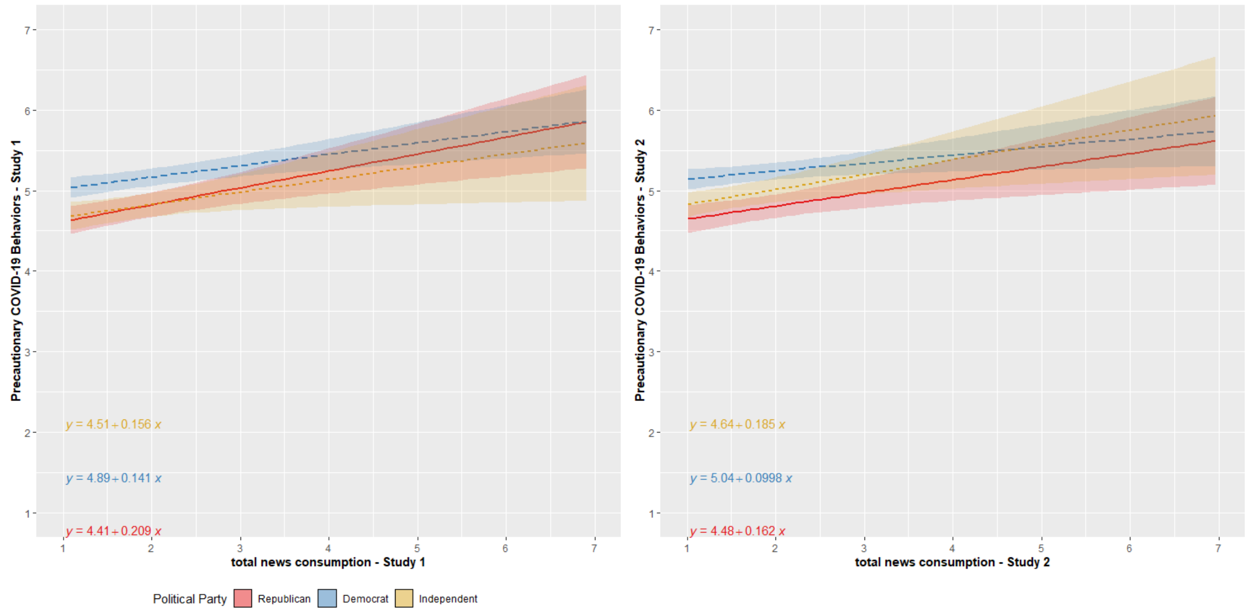

Figure S19. Studies 1 and 2 conditional effects of moderated linear regressions in which COVID-19 precautions were regressed on total news consumption (centered), political party affiliation, and the two-way interaction between party affiliation and total news consumption. Bands around regression lines are 95% confidence intervals. On each plot, regression equations indicate the conditional simple slopes relationships between total news consumption and COVID-19 precautions by political affiliation (slopes are unstandardized).

Relationships between COVID-19 health precautions, political party, and cost-benefit assessments of COVID-19 threats composite

In the main text, we hypothesized that partisan differences in responses to the COVID-19 outbreak may be shaped by differential cost-benefit analyses of the various threats posed by the pandemic, including the direct health threats, as well as the downstream economic and perceived personal liberty threats.

Examining Democrats, Republicans, and political Independents, there was a significant effect linking party affiliation to greater beliefs that the direct health threats of the pandemic

were less serious, especially relative to the economic and personal liberty threats (Study 1: $F[2, 861] = 73.23$, $p < 2e-16$; Study 2: $F[2, 857] = 109.90$, $p < 2e-16$). Post hoc comparisons using the Tukey HSD test indicate that Republicans (Study 1: $M = 3.38$, $SD = 1.20$; Study 2: $M = 3.30$, $SD = 1.19$) were significantly more likely than Democrats (Study 1: $M = 2.35$, $SD = .84$, $p < 2e16$; Study 2: $M = 2.09$, $SD = .735$, $p < 2e-16$) or Independents (Study 1: $M = 2.93$, $SD = 1.20$, $p = 2.23-5$; Study 2: $M = 2.09$, $SD = 1.13$, $p = 2.10e-10$) to weight health threats less seriously relative to economic and personal liberty threats, and that Independents were in turn significantly higher along that scale compared to Democrats (Study 1: $p = 6.47e-11$; Study 2: $p = 1.07-e13$). In sum, compared to Democrats, Republicans and Independents weighted the health threats posed by the pandemic as less severe, particularly in relation to economic and personal liberty threats.

Further, we examined the party-specific relationships between the relative cost assessments of different COVID-19 threats and precautionary health behaviors. We found that weighing the direct health threats of the pandemic as less serious relative to the economic and personal liberty threats negatively correlated with precautionary health behaviors among Republicans and Independents, but not Democrats. These results suggest that domain-specific cost-benefit threat weightings may indeed influence partisan differences in precautionary health behaviors in response to the pandemic.

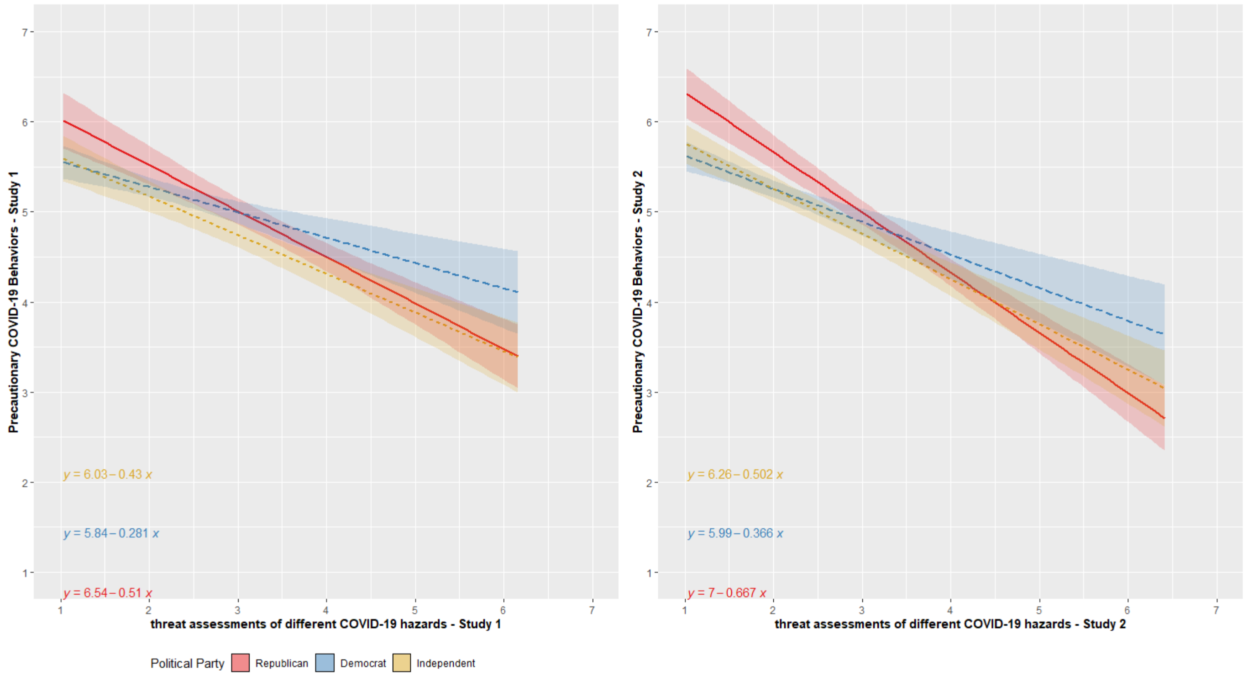

Figure S20. Studies 1 and 2 conditional effects of moderated linear regressions in which COVID-19 precautions were regressed on the (centered) threat assessments of different COVID-19 hazards composite, political party affiliation, and the two-way interaction between party affiliation and the threat assessments composite. Bands around regression lines are 95% confidence intervals. On each plot, regression equations indicate the conditional simple slopes relationships between the threat assessments composite and COVID-19 precautions by political affiliation (slopes are unstandardized). Higher scores along the threat assessments composite indicate finding the direct health threats of the pandemic as less serious, especially compared to economic or personal liberty threats.

Finally, the party-specific relationships between the domain-specific threat assessments composite and socially conservative attitudes were examined. Among Democrats and Independents, but not Republicans, socially conservative attitudes were associated with weighing health threats as less serious relative to economic and personal liberty hazards. We speculate that the lack of an association among Republicans may be attributable to a ceiling effect. Further, it is noteworthy that while socially conservative attitudes positively correlated with health precautions among Democrats, they also positively associated with weighing the health hazards posed by the pandemic as less serious relative to the economic and personal liberty threats.

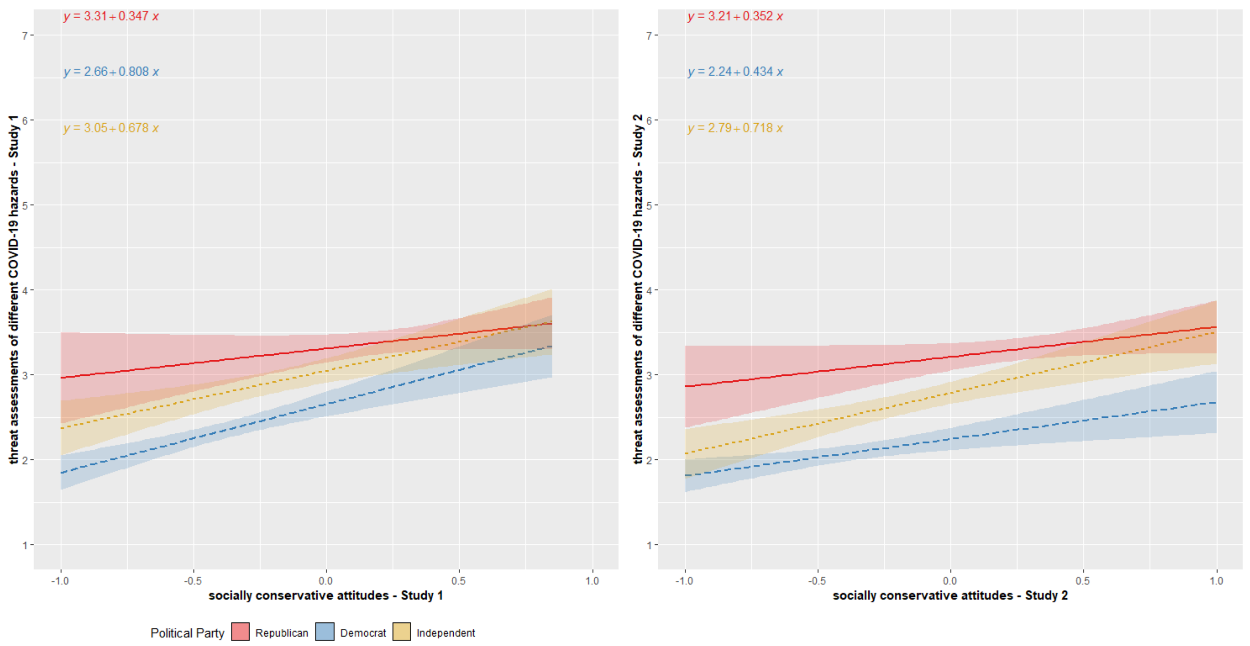

Figure S21. Studies 1 and 2 conditional effects of moderated linear regressions in which the threat assessments of different COVID-19 hazards composite was regressed on the (centered) socially conservative attitudes composite, political party affiliation, and the two-way interaction between party affiliation and socially conservative attitudes. Bands around regression lines are 95% confidence intervals. On each plot, regression equations indicate the conditional simple slopes relationships between the threat assessments composite and socially conservative attitudes by political affiliation (slopes are unstandardized). Higher scores along the threat assessments composite indicate finding the direct health threats of the pandemic as less serious, especially compared to economic or personal liberty threats.

2. Relationships between COVID-19 health precautions and opinions regarding government public health interventions

We speculated that general opinions about government public health interventions may contribute to partisan differences in responses to the COVID-19 outbreak. In Studies 1 and 2, we measured opinions about public health interventions in non-COVID-19 related areas by gauging participants' agreement with government interventions regarding tobacco use. Although these opinions did not suppress the relationship between socially conservative attitudes and COVID-19 precautionary behaviors among Republicans, we examined the simple effects of public health intervention endorsement on precautionary COVID-19 health behaviors by political party.

Supportive attitudes toward government intervention in tobacco use–which proxies attitudes toward public health policies in non-COVID-19 areas–positively correlated with precautionary behaviors among Republicans in both studies. In Study 1, that positive association obtained among Democrats but only approached significance among Independents, while in Study 2, the relationship was significant among Independents, but approached significance among Democrats. Based on the density plot distributions, Democrats were more supportive of public health interventions regarding tobacco use relative to Republicans and Independents.

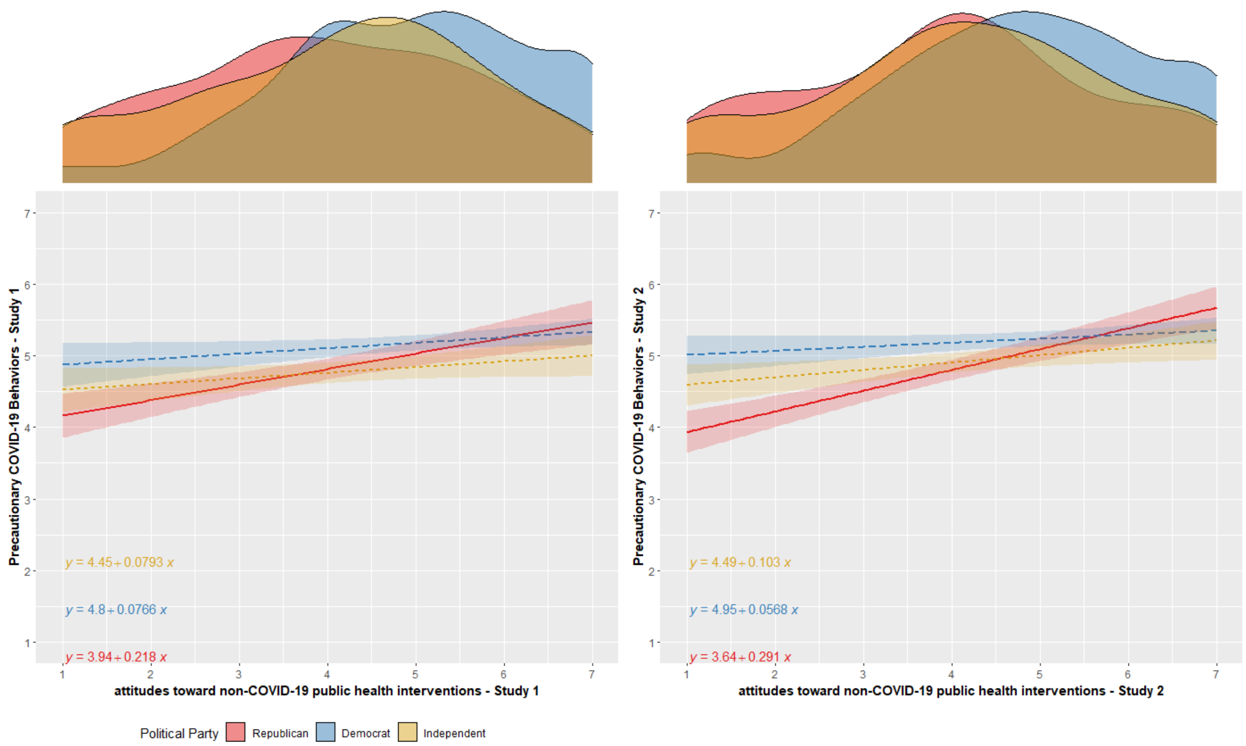

Figure S22. Studies 1 and 2 conditional effects of moderated linear regressions in which the (centered) precautionary COVID-19 behaviors composite was regressed on attitudes toward non-COVID-19 public-health interventions, political party affiliation, and the two-way interaction between party affiliation and attitudes toward public health interventions. Bands around regression lines are 95% confidence intervals. On each plot, regression equations indicate the conditional simple slopes relationships between the COVID-19 precautions and attitudes toward public health interventions by political affiliation (slopes are unstandardized). Higher scores along the x-axes indicate stronger agreement with government interventions in non-COVID-19 public health domains.

3. Relationships between COVID-19 health precautions and demographic variables

We collected basic demographic information about the participants, which we can use to examine the effects of different demographic variables on COVID-19 health precautions. As we highlighted in the main text, Republicans and Democrats differ, on average, along a number of different demographic dimensions. Especially relevant for COVID-19, Democrats are more likely to live in high-density areas, and at the time data were collected, the estimated distribution of coronavirus outbreaks across the United States was highly skewed along geographic and urban/rural lines. Although none of the demographic variables were responsible for suppressing the socially conservative attitudes-precautions relationship among Republicans and Independents, and demographics did not account for the positive correlation between socially conservative attitudes and precautions among Democrats, it is still of interest to examine the relationships between various demographic factors, and health prophylaxis. Here, we systematically assess those relationships.

Age

There was no zero order relationship between age and precautionary COVID-19 behaviors in either study.

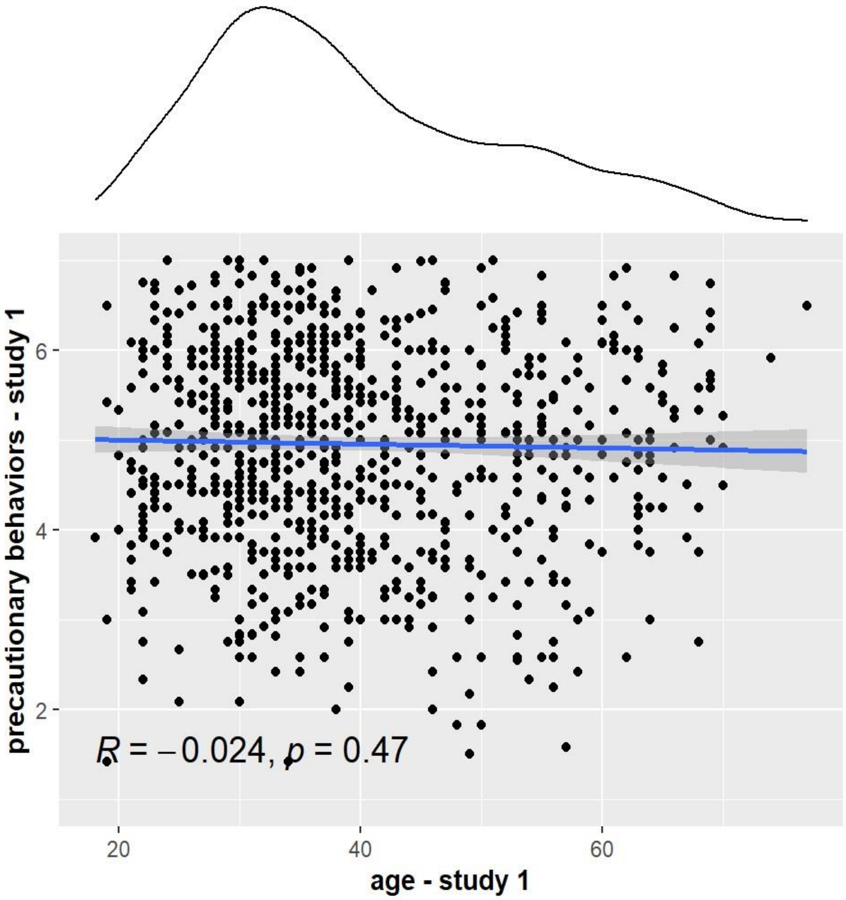
precautionary behaviors - study 1
R = –0.024, p = 0.47
age - study 1
20
40
60
2
4
6

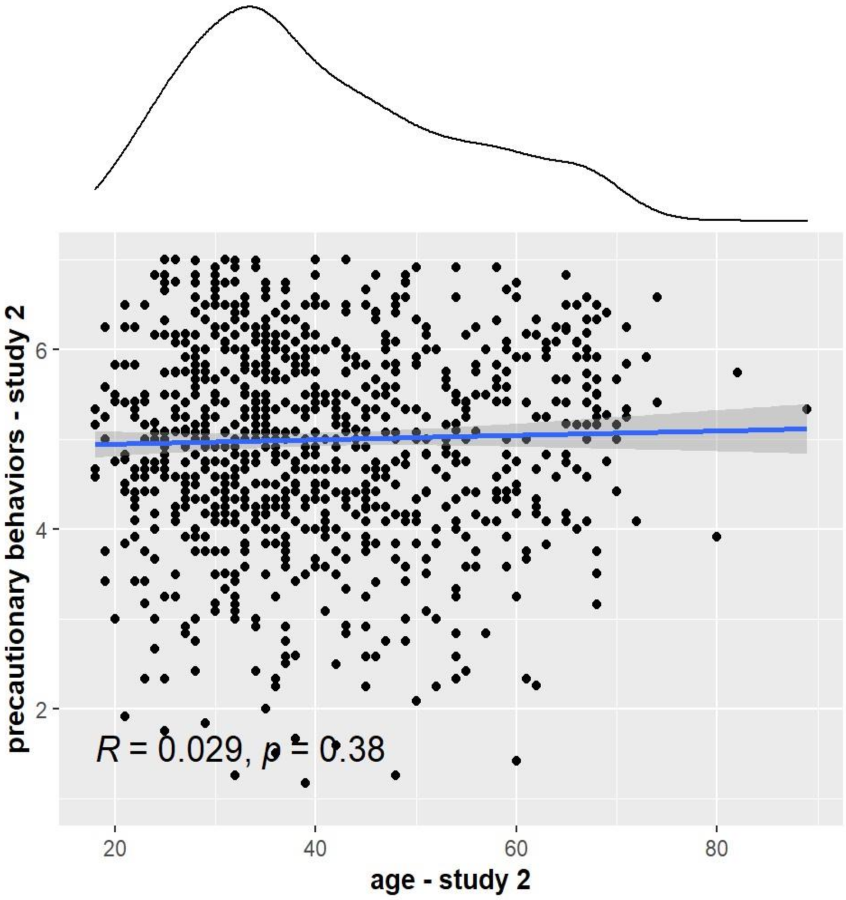
precautionary behaviors - study 2
R = 0.029, p = 0.38
age - study 2
20
40
60
80
2
4
6

Figure S23. Relationships between age and precautionary COVID-19 behaviors. The density plots along the x-axes represent the age distribution of the sample. Further, scatterplot points have been randomly jittered along the y-axes to aid interpretability.

Gender

On average, women engaged in significantly more health precautions than men, consistent with the broader literature on sex differences in risk-taking (Sparks et al., 2018).

.

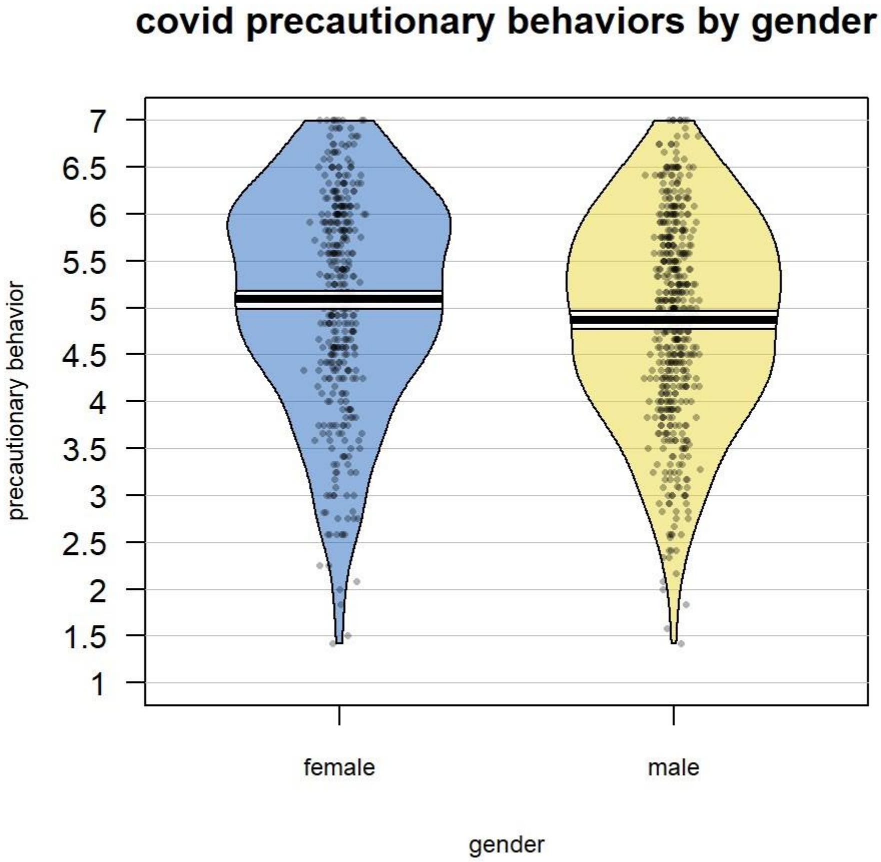

Figure S24. Pirate plot comparing differences in COVID-19 health precautions by gender, Study 1. Scatterplot points are raw data, jittered to reduce overlap. Beans show smoothed density of scatterplot points. Bars and boxes represent means and Bayesian 95% highest density intervals, respectively.

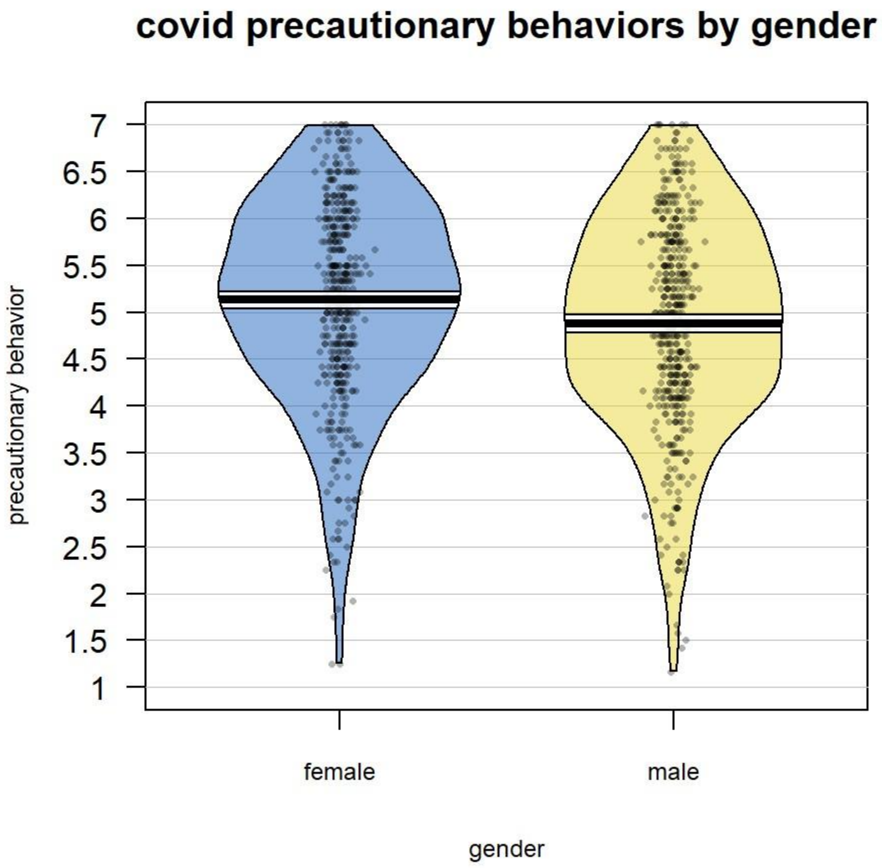

Figure S25. Pirate plot comparing differences in COVID-19 health precautions by gender, Study 2. Scatterplot points are raw data, jittered to reduce overlap. Beans show smoothed density of scatterplot points. Bars and boxes represent means and Bayesian 95% highest density intervals, respectively.

Ethnicity

Because participants in both studies overwhelmingly self-identified as white (Study 1: 69%, Study 2: 76%), we did not have large enough samples to adequately compare among individuals who self-identified with particular non-white identities. Therefore, in this analysis, we used a simple binary ethnicity variable, with participants who self-identified as non-white on the one hand, and participants who self-identified as white on the other. On average, people who self-identified as non-white engaged in significantly more health precautions than people who identified as white.

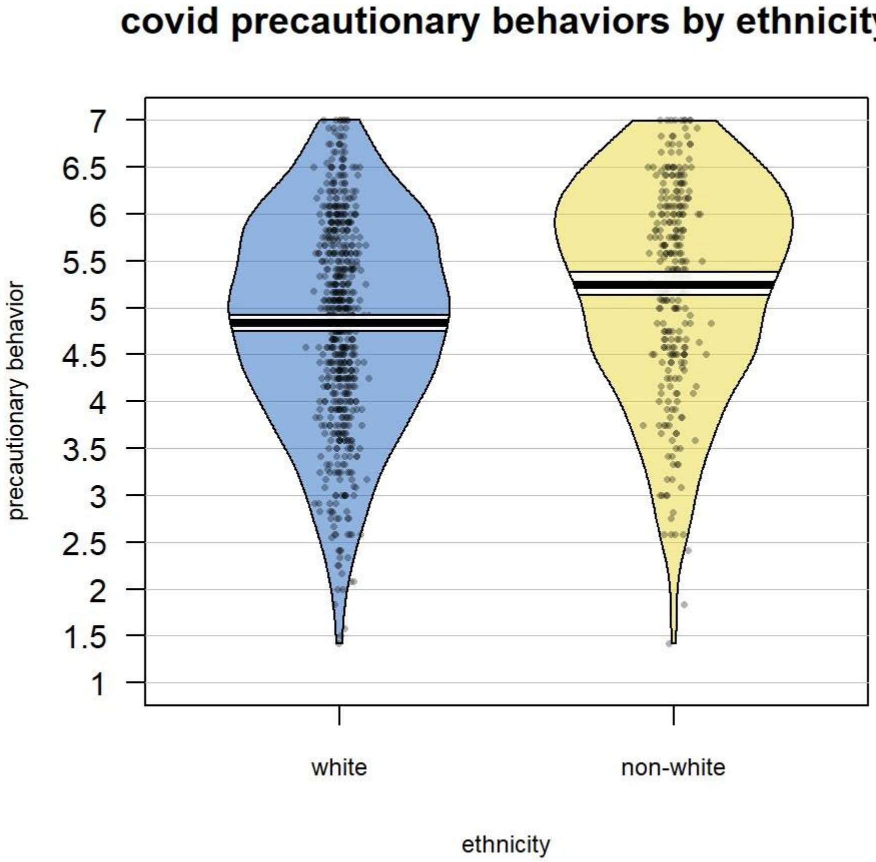

Figure S26. Pirate plot comparing differences in COVID-19 health precautions by self-identified ethnicity, Study 1. Scatterplot points are raw data, jittered to reduce overlap. Beans show smoothed density of scatterplot points. Bars and boxes represent means and Bayesian 95% highest density intervals, respectively.

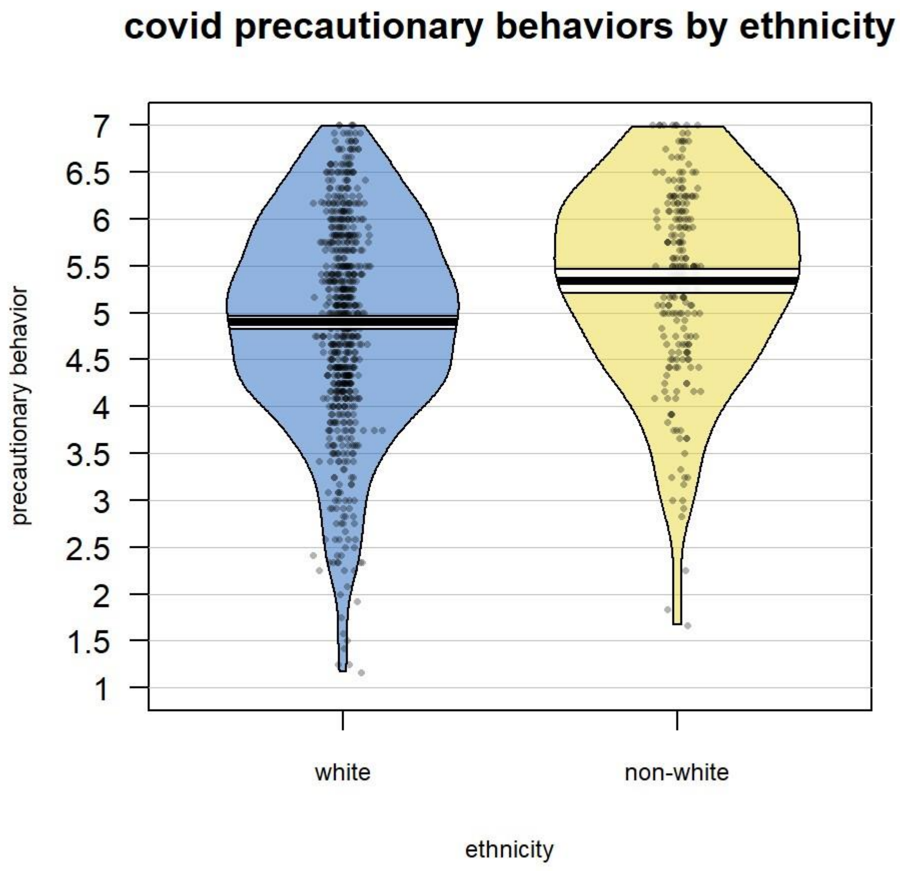

Figure S27. Pirate plot comparing differences in COVID-19 health precautions by self-identified ethnicity, Study 2. Scatterplot points are raw data, jittered to reduce overlap. Beans show smoothed density of scatterplot points. Bars and boxes represent means and Bayesian 95% highest density intervals, respectively.

Income

Income did not correlate with COVID-19 health precautions in Study 1, and weakly correlated with precautions in Study 2.

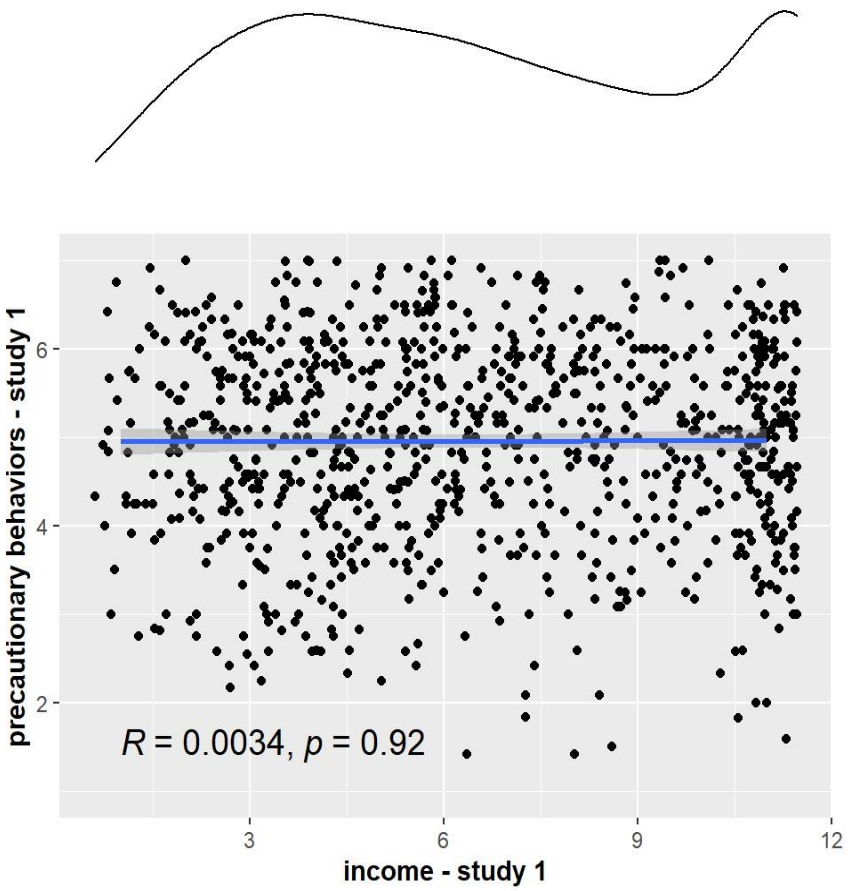
precautionary behaviors - study 1
income - study 1
R = 0.0034, p = 0.92
2
4
6
3
6
9
12

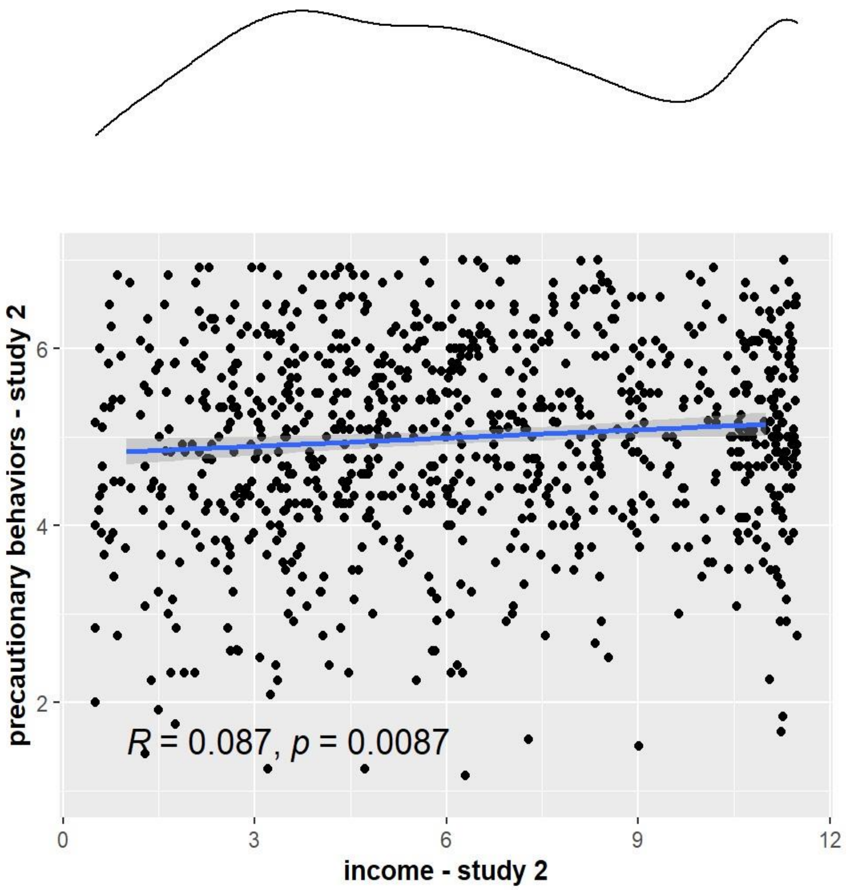
precautionary behaviors - study 2
income - study 2
R = 0.087, p = 0.0087
2
4
6
0
3
6
9
12

Figure S28. Relationships between income and precautionary COVID-19 behaviors. The density plots along the x-axes represent the income distribution of the sample. Further, scatterplot points have been randomly jittered along the y-axes to aid interpretability.

Education

Overall, education did not correlate with COVID-19 health precautions in either study.

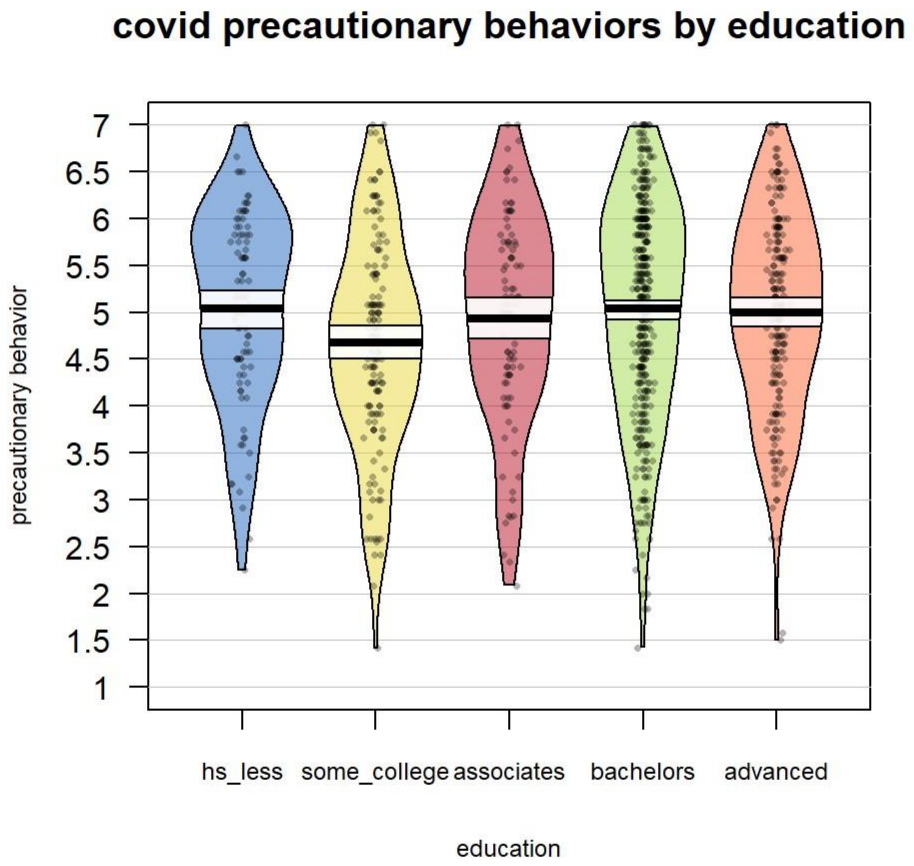

Figure S29. Pirate plot comparing differences in COVID-19 health precautions by educational attainment, Study 1. Scatterplot points are raw data, jittered to reduce overlap. Beans show smoothed density of scatterplot points. Bars and boxes represent means and Bayesian 95% highest density intervals, respectively.

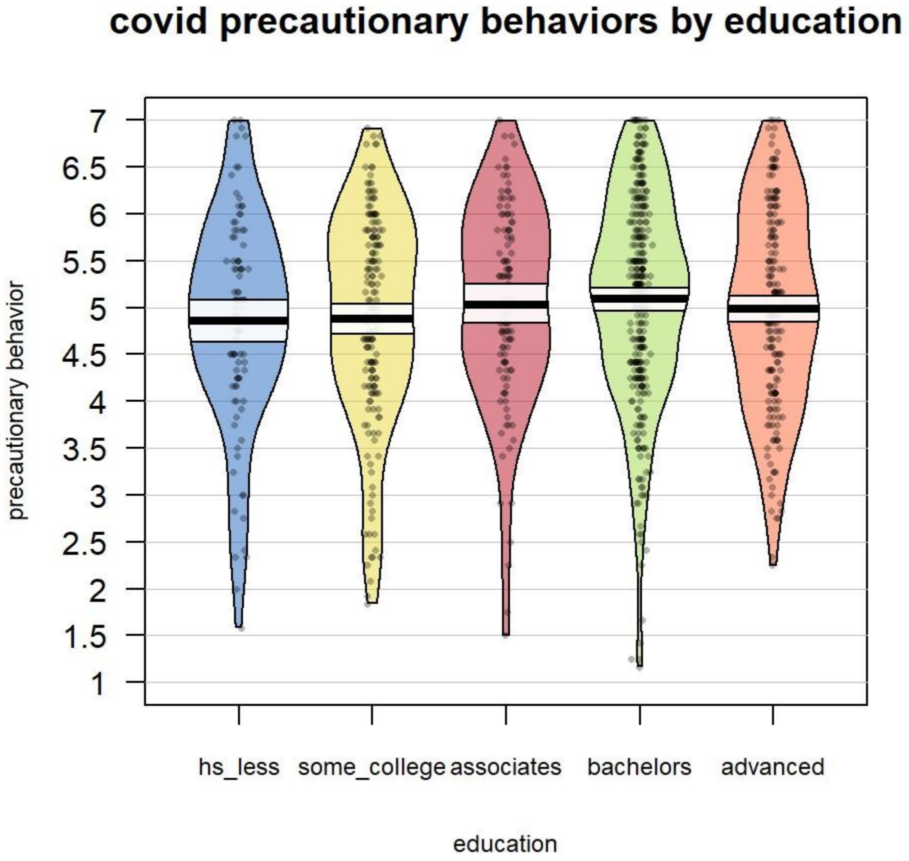

Figure S30. Pirate plot comparing differences in COVID-19 health precautions by educational attainment, Study 2. Scatterplot points are raw data, jittered to reduce overlap. Beans show smoothed density of scatterplot points. Bars and boxes represent means and Bayesian 95% highest density intervals, respectively.

Job Requirements

Having jobs that required participants to leave the home for work did not associate with COVID-19 precautions in either study.

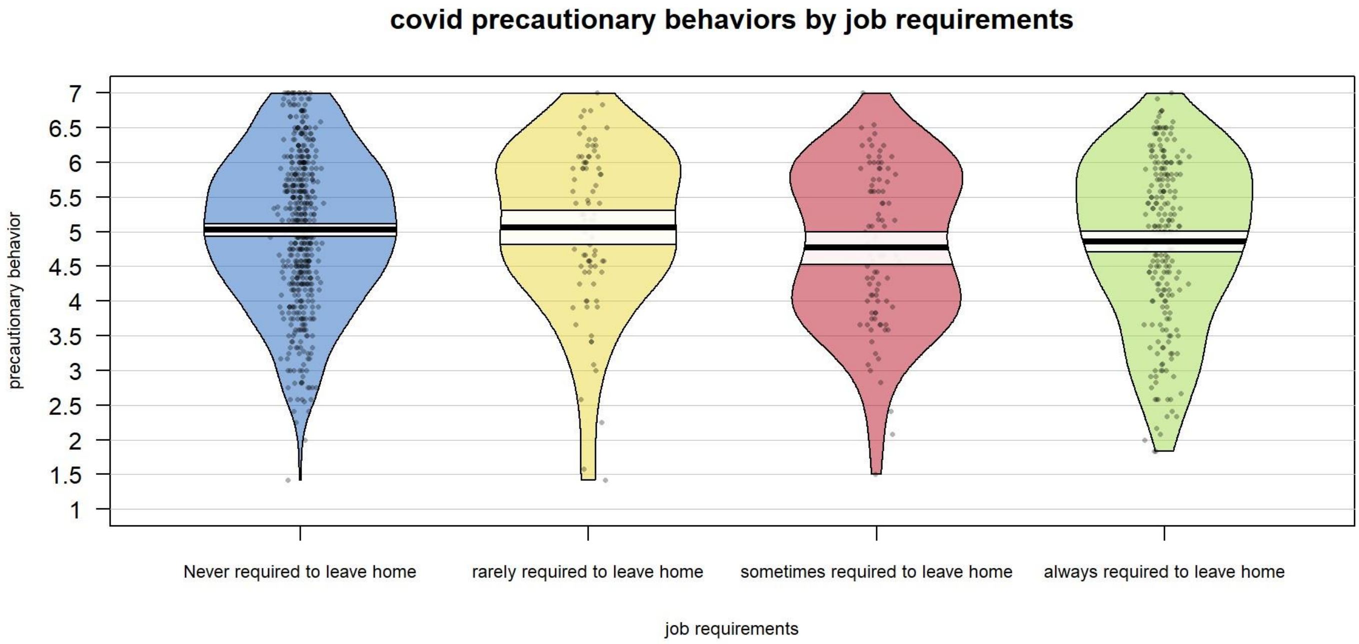

Figure S31. Pirate plot comparing differences in COVID-19 health precautions by job requirements, Study 1. Scatterplot points are raw data, jittered to reduce overlap. Beans show smoothed density of scatterplot points. Bars and boxes represent means and Bayesian 95% highest density intervals, respectively.

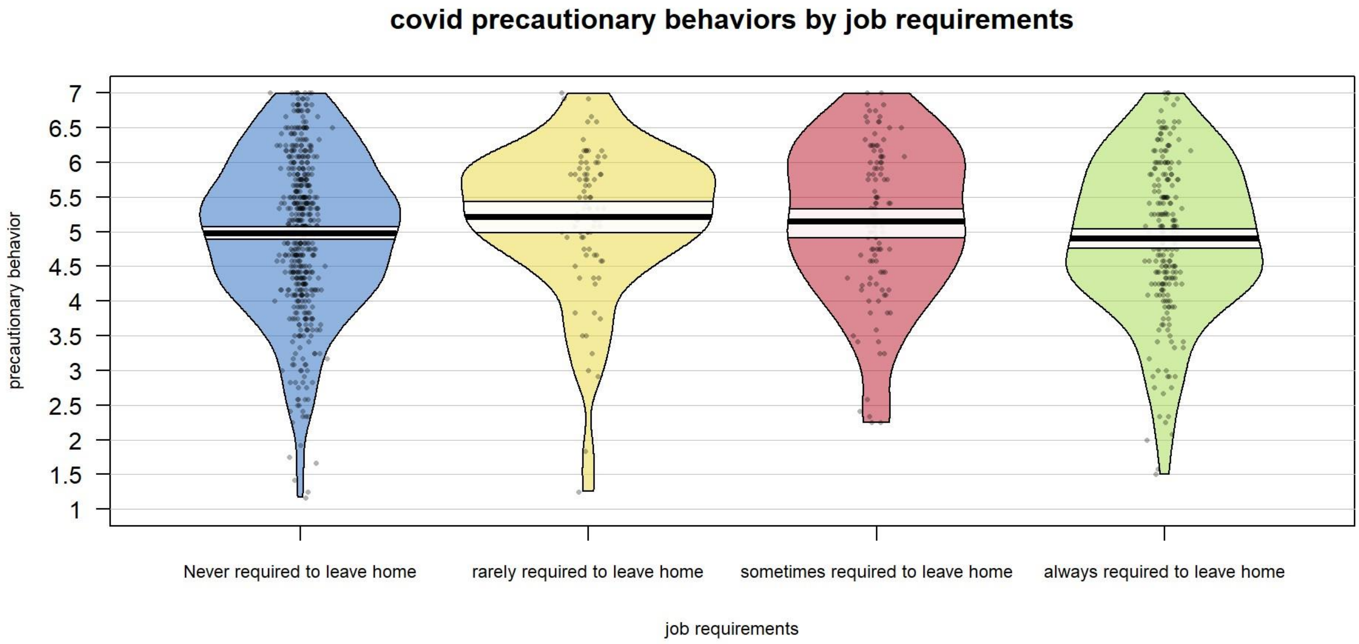

Figure S32. Pirate plot comparing differences in COVID-19 health precautions by job requirements, Study 2. Scatterplot points are raw data, jittered to reduce overlap. Beans show smoothed density of scatterplot points. Bars and boxes represent means and Bayesian 95% highest density intervals, respectively.

Pre-existing health conditions

Participants who reported having any health conditions that may render them more susceptible to severe COVID19 illness reported, on average, engaging in more COVID-19 health precautions. The possible health conditions presented to participants were as follows: autoimmune disease, immunological deficiency, diabetes, hypertension, coronary heart disease, asthma, kidney disease, or other. A binary variable was created, with participants who reported having at least one of the aforementioned health conditions on the one hand, and participants who did not report any of those health conditions on the other.

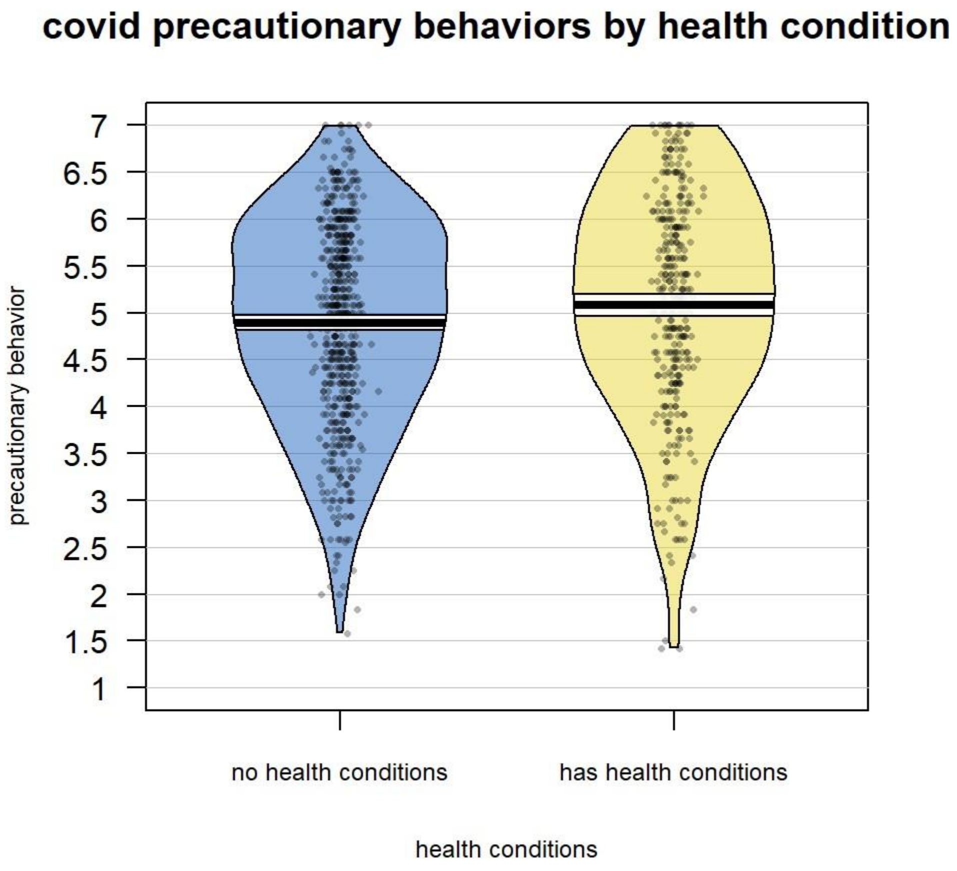

Figure S33. Pirate plot comparing differences in COVID-19 health precautions by health conditions, Study 1. Scatterplot points are raw data, jittered to reduce overlap. Beans show smoothed density of scatterplot points. Bars and boxes represent means and Bayesian 95% highest density intervals, respectively.

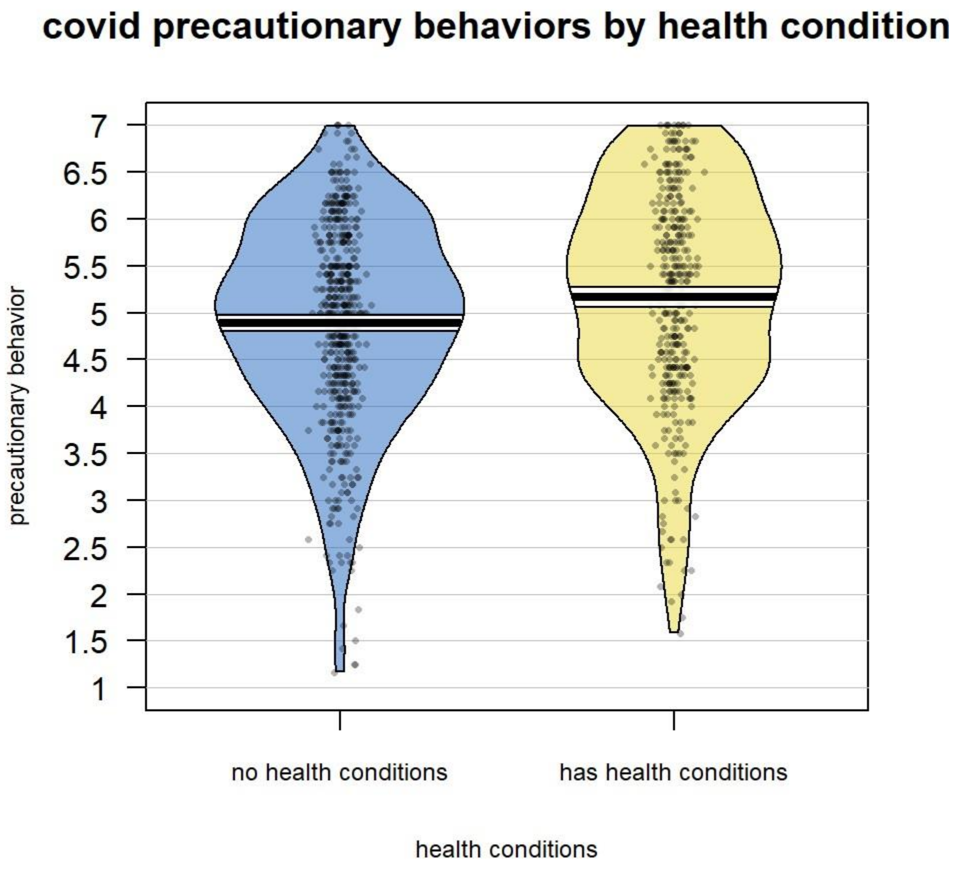

Figure S34. Pirate plot comparing differences in COVID-19 health precautions by health conditions, Study 2. Scatterplot points are raw data, jittered to reduce overlap. Beans show smoothed density of scatterplot points. Bars and boxes represent means and Bayesian 95% highest density intervals, respectively.

4. Item-by-item analyses of political party differences in precautionary COVID-19 health behaviors

In the main text, we reported finding in both studies an effect of political party support on average levels of the precautionary COVID-19 behaviors composite, such that Democrats reported engaging in more precautions on average than Republicans or Independents. Here, we break down that composite scale into its individual items, asking which items may be driving observed differences between Republicans, Democrats, and Independents. A series of one-way

ANOVAs on ranks and Wilcoxon rank sum tests (to examine pairwise differences) were conducted, in order to explore differences in precautionary behaviors by major political party affiliation, with each constituent item being analyzed individually, the results of which are summarized in the tables below.

**Precaution item**	**Kruskal-Wallis χ2**	**Kruskal-Wallis p-value**	**Republican-Democrat p-value**	**Republican-Independent p-value**	**Independent-Democrat p-value**
Frequency of washing hands	9.87	.007	.015	.900	.015
Frequency of sanitizing hands	5.54	.063	.809	.070	.070
Frequency of disinfecting surfaces	4.72	.095	.388	.434	.096
Frequency of taking supplements intended to boost immune system	1.11	.574	.684	.878	.684
Effort spent on acquiring household disinfectants	7.01	.030	.113	.569	.043
Effort spent on acquiring soap and hand sanitizer	10.00	.007	.340	.089	.005

Effort spent on acquiring masks and gloves	15.3	4.66e-4	.002	.769	.004
Frequency of using mask	51.3	7.37e-12	9.95e-10	.479	3.74e-8
Frequency of using gloves	10.8	.005	.029	.657	.010
Frequency of staying farther than 6 feet from people in public	12.2	.002	.005	.496	.017
Extent to which lockdown rules were observed	24.7	4.31e-6	2.03e-5	.449	4.36e-4
Extent to which social distancing was observed in general	17.2	1.81e-4	1.38e-4	.122	.033

Table S7. Results of one-way ANOVAs on ranks and Wilcoxon rank sum tests testing individual precautionary behavior items by political party, Study 1. For the Wilcoxon pairwise comparisons, p-values have been adjusted for multiple comparisons using the Benjamini-Hochberg procedure.

**Precaution item**	**Kruskal-Wallis χ2**	**Kruskal-Wallis p-value**	**Republican-Democrat p-value**	**Republican-Independent p-value**	**Independent-Democrat p-value**
Frequency of washing hands	3.86	.145	.151	.373	.479
Frequency of sanitizing hands	4.74	.094	.332	.439	.105
Frequency of disinfecting surfaces	3.65	.161	.454	.454	.166
Frequency of taking supplements intended to boost immune system	.03	.987	.952	.952	952
Effort spent on acquiring household disinfectants	6.97	.031	.067	.797	.063
Effort spent on acquiring soap and hand sanitizer	9.45	.009	.020	.838	.020
Effort spent on acquiring masks and gloves	17.1	1.94e-4	.001	.609	.002
Frequency of using mask	65.4	6.27e-15	1.97e-15	2.75e-4	9.06e-5

Frequency of using gloves	6.38	.041	.070	.990	.070
Frequency of staying farther than 6 feet from people in public	5.48	.065	.053	.229	.514
Extent to which lockdown rules were observed	39.6	2.49e-9	1.93e-9	.017	5.70e-4
Extent to which social distancing was observed in general	37.2	8.15e-9	4.07e-9	2.18e-4	.059

Table S8. Results of one-way ANOVAs on ranks and Wilcoxon rank sum tests testing individual precautionary behavior items by political party, Study 2. For the Wilcoxon pairwise comparisons, p-values have been adjusted for multiple comparisons using the Benjamini-Hochberg procedure.

Although results varied somewhat between studies, compared to Republicans or Independents, Democrats generally reported washing their hands more; making a greater effort to acquire soap, hand sanitizer, masks, and gloves; using masks and gloves more in public; observing social distancing more stringently; and following lockdown orders more. The effects were strongest for mask wearing in particular, as well as social distancing, effort spent acquiring masks and gloves, and following lockdown orders. There generally were not strong differences

between Democrats on the one hand, and either Republicans or Independents on the other, in the use of supplements intended to boost the immune system, and the frequency with which surfaces were disinfected. Independents and Republicans did not systematically differ in mean precautions across both studies.

5. Relationship between economic conservatism and pathogen disgust sensitivity

In the main text, we speculate that among Republicans, concern for economic considerations may conflict with precautionary responses to COVID-19. Indeed, among Republicans, we found that economic conservatism negatively correlated with concerns about the health consequences of the pandemic, as well as with precautionary health behaviors (see Supplement S3- Supporting Analyses). Given the relationship between pathogen disgust sensitivity and precautionary behaviors in response to COVID-19, we considered the possibility that economic conservatism may also negatively correlate with disgust, particularly among Republicans.

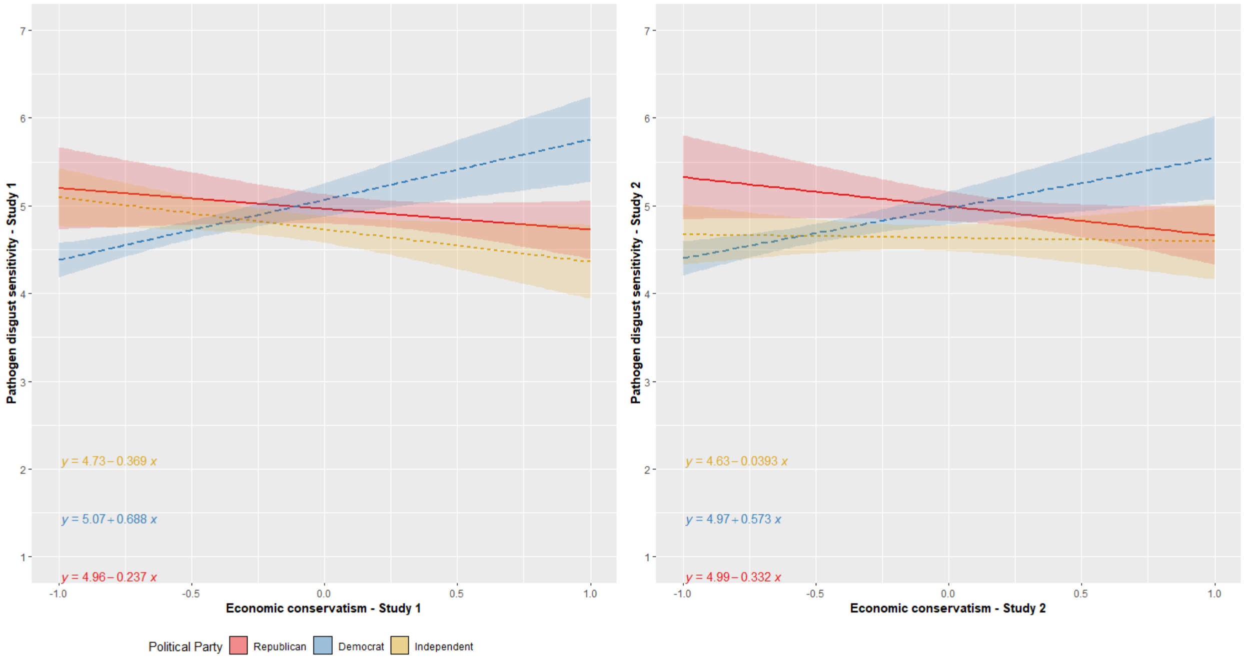

Figure S35. Studies 1 and 2 conditional effects of moderated linear regressions in which the (centered) pathogen disgust sensitivity composite was regressed on economic conservatism, political party affiliation, and the two-way interaction between party affiliation and economic conservatism. Bands around regression lines are 95% confidence intervals. On each plot, regression equations indicate the conditional simple slopes relationships between pathogen disgust sensitivity and economic conservatism by political affiliation (slopes are unstandardized).

Results were somewhat conceptually similar to the party-specific relationships between economic conservatism and precautionary COVID-19 behaviors. Disgust positively correlated with economic conservatism among Democrats, consistent with the positive association between precautionary behaviors and economic conservatism among supporters of that party. However, among Independent and Republicans, economic conservatism did not consistently negatively correlate with pathogen disgust.

6. Results without lockdown order item in COVID-19 health precautions composite

In the main text analyses, the COVID-19 precautionary health behaviors composite included a large range of items intended to capture different categories of prophylactic behavior, including the extent to which participants were following local and state lockdown restrictions. Owing to the prominent role lockdowns have played in shaping behavioral responses to the pandemic, we believe that it was important to measure compliance with lockdown orders in the course of measuring precautionary behavior, hence its inclusion in the precautionary behaviors composite. However, while the other items in the precautions composite—such as engaging in hand washing and wearing masks—are consistently applicable across the United States, lockdown orders have varied widely both geographically, and over time. Therefore, the question of, “To what extent are you following your local and state lockdown restrictions?” did not have consistent meaning for participants both within and across studies. For example, following highly restrictive lockdown orders entails substantially different behavior compared to complying with looser rules. Because we did not ask what those lockdown restrictions were, we

cannot measure what could plausibly be substantial variation between participants in the meaning of that item.

In order to assuage concerns that this unmeasured variation may be influencing the reported results–especially in light of the fact that variation in lockdown orders has likely tracked along geographical lines that mirror political divisions–we repeated the main text analyses using a version of the precautionary COVID-19 behaviors composite that excluded the lockdown order item (this version of the scale was reliable: αs = .85). Excluding the lockdown order item had little effect on the conceptual outcomes of Studies 1 and 2, the general results of which were replicated with the lockdown-less precautions scale. Therefore, despite concerns about variation in meaning for this item between participants, its inclusion in the precautionary behaviors composite had little impact on the conclusions drawn from the results. The analyses without the lockdown item are reported below.

Does COVID-19 precautionary behavior differ by political party?

Examining Democrats, Republicans, and political Independents, there was a significant effect linking party affiliation to levels of precautionary behavior without the lockdown item in both studies (Study 1: F[2, 860] = 11.27, p = 1.47e-5Study 2: F[2, 857] = 10.9, p = 2.12e-5). Post hoc comparisons using the Tukey HSD test indicate that the mean precaution scores for Democrats (Study 1: M = 5.07, SD = 1.08; Study 2: M = 5.11, SD = 1.04) were significantly different than those for Republicans (Study 1: M = 4.71, SD = 1.31, p = 0.00111; Study 2: M = 4.7, SD = 1.31, p = 7.16e-5) and Independents (Study 1: M = 4.67, SD = 1.24, p = 1.02e-4; Study 2: M = 4.81, SD = 1.11, p = .003), but that precautions did not significantly differ between the latter two (Study 1: p = 0.901; Study 2: p = 0.606). These findings are fully consistent with the results reported in the main text.

Do socially conservative political attitudes predict precautionary behavior?

In both studies, the precautionary COVID-19 composite without the lockdown item correlated with socially conservative political attitudes among Democrats, but not Republicans or Independents. These findings are fully consistent with the main text results.

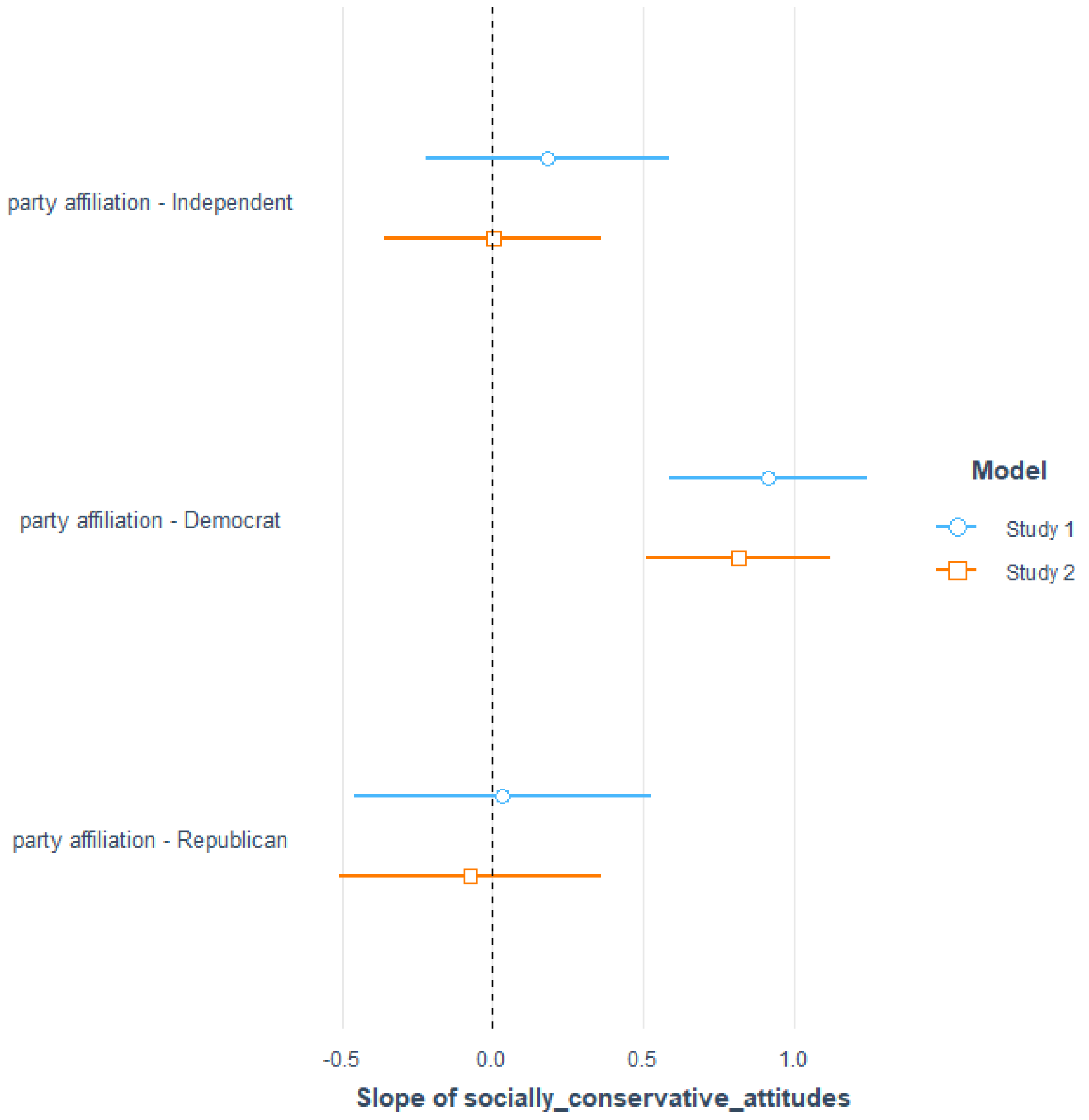

Figure S36. Studies 1 and 2 forest plot showing conditional effects (based on simple slopes analyses) of moderated linear regressions, in which COVID-19 precautionary behaviors (minus the lockdown item) was regressed on socially conservative attitudes, political party affiliation, and their two-way interaction. Plotted coefficients are unstandardized. Lines indicate 95% confidence intervals.

What drives partisan differences in the relationship between socially conservative political attitudes and COVID-19 precautions?

We tested whether the combined effects of economic conservatism, the trust in scientists composite, the trust in liberals and moderates composite, and the liberal media consumption composite jointly suppressed the relationship between socially conservative attitudes and the precautionary behaviors composite minus the lockdown item. First, the combined indirect effect of the four suppressors was negative and significant among Republicans and Independents in both studies (**Study 1** - Republicans: bootstrapped standardized indirect effect = -.16, 95% CI [-.23, -.09]; Independents: indirect effect = -.14, 95% CI [-.22, -.06]); **Study 2** - Republicans: bootstrapped standardized indirect effect = -.11, 95% CI [-.19, -.04]; Independents: indirect effect = -.27, 95% CI [-.36, -.18]), demonstrating suppression.

Second, the combined effects of the suppressors rendered the moderation of political party on the attitudes-precautions (minus lockdown item) relationship no longer significant between Democrats on the one hand, and either Republicans or Independents on the other (Democrat-Republican: βs = -.08 – -.01, ps = .140 – .899; Democrat-Independent: βs = -.01 – -.001, ps = .882 – .990).

Third, in Study 1, a simple slopes analysis reveals that, after including for the effects of the suppressors and their interactions with political party, there were significant conditional effects between socially conservative attitudes and the precautionary behaviors composite without the lockdown item among Democrats (β = 0.26, t(820) = 4.14, p = 3.90e-5), Republicans (β = 0.24, t(820) = 2.63, p = .009), and Independents (β = 0.24, t(820) = 3.27, p = .001). In Study 2, there were conditional effects among Democrats (β = 0.28, t(812) = 4.61, p = 4.65e-6) and Independents (β = 0.28, t(812) = 3.66, p = 2.69e-4), but no significant effect among Republicans

($\beta = 0.13$, $t(812) = 1.64$, $p = .103$). These findings are partially consistent with the results reported in the text.

Further, the conceptual results of these models (significant and positive relationships between COVID-19 precautions and socially conservative attitudes among Democrats and Independents in both studies after accounting for the suppressors, but only in Study 1 among Republicans) were robust to the inclusions of the same demographic variables and other covariates (such as pathogen disgust sensitivity) described in the Main Text.

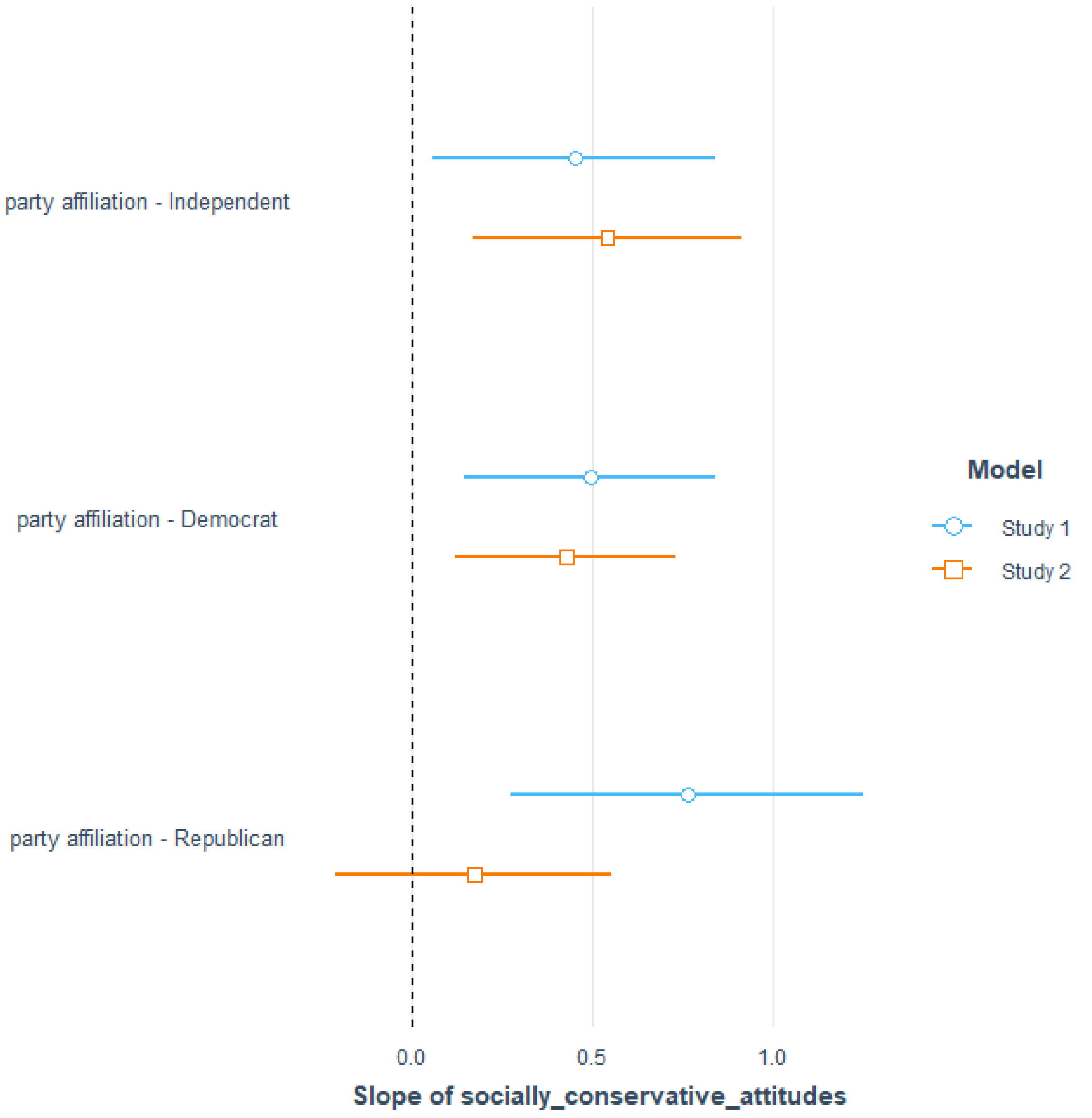

Figure S37. Studies 1 and 2 forest plot showing conditional effects (based on simple slopes analyses) of socially conservative attitudes on the COVID-19 precautions composite (minus the lockdown-related item) from moderated linear regressions. Additionally, these models include both the pre-identified suppressor variables, as well as a wide variety of covariates. These additional covariates were as follows: age, gender, ethnicity, income, education, pre-existing health conditions, self-reported density of local neighborhood, self-reported estimates of local COVID-19 prevalence, the extent to which one's job required leaving the household, and pathogen disgust sensitivity. Plotted coefficients are unstandardized. Lines indicate 95% confidence intervals.

Disgust sensitivity, politics, and precautionary COVID-19 behaviors

Using moderated linear regressions in which the version of the COVID-19 precautionary behaviors composite that excludes the lockdown item was regressed on pathogen disgust sensitivity, political party affiliation, and their two-way interaction, we found that disgust sensitivity was associated with precautionary behaviors among supporters of all three major political party affiliations (*B*s = .22 – .42, *p*s = 3.07e-4 – 5.24e-11)

7. Results separating social conservatism and traditionalism measures

In the main text, we examined the relationship between socially conservative attitudes–which encompasses both specific policy preferences that emphasize social continuity, and general attitudes toward traditions—and precautionary COVID-19 health behaviors. That is, we combined into a single composite both specific social policy preferences (as measured by the social conservatism subscale of the issues index measure), and general attitudes toward social change (as measured by the traditionalism subscale of the Aggression-Submission-Conventionalism scale, which measures the concepts of right-wing authoritarianism using more politically and religiously neutral language). This was useful, given that preferences for social change versus social continuity ramify across many different domains, and combining beliefs regarding different aspects of support for traditions captures a broader slice of the phenomenon of interest.

However, it is also of interest to understand how pathogen-avoidant precautionary behaviors individually relate to different components of socially conservative attitudes writ large. For example, is the relationship between specific social policy preferences and precautionary COVID-19 behaviors conceptually isomorphic with the relationship between general traditionalism attitudes and prophylaxis? Or, do these two facets of socially conservative

attitudes relate differently to precautionary behavior? Here, we repeat the analyses found in the main text, but instead of using the combined socially conservative attitudes composite as a single independent variable, we separate out its two constituent parts: social conservatism (i.e., specific policy preferences toward social change), and traditionalism (i.e., general attitudes toward social change).

In sum, these results suggest that, when parceled out, social conservatism and traditionalism associate similarly, with precautionary behaviors. This points to both the theoretical cohesiveness between these measures–preferences for traditional political policies on the one hand, and positive attitudes toward traditions in general on the other–as well as the practical utility in combining these measures into the socially conservative attitudes composite found in the main text analyses. That the analyses using the combined measure, and the analyses separating out the two individual measures, largely conceptually converge constitutes an importance robusticity check of the main findings.

Do social conservatism and traditionalism predict precautionary behavior?

In both studies, social conservatism and traditionalism separately correlated with precautionary COVID-19 behaviors among Democrats, but not Republicans or Independents (see Figure S38). These results are consistent with the main text, and suggest that at least for these simple effects, both social conservatism and traditionalism are associating similarly with precautionary behavior.

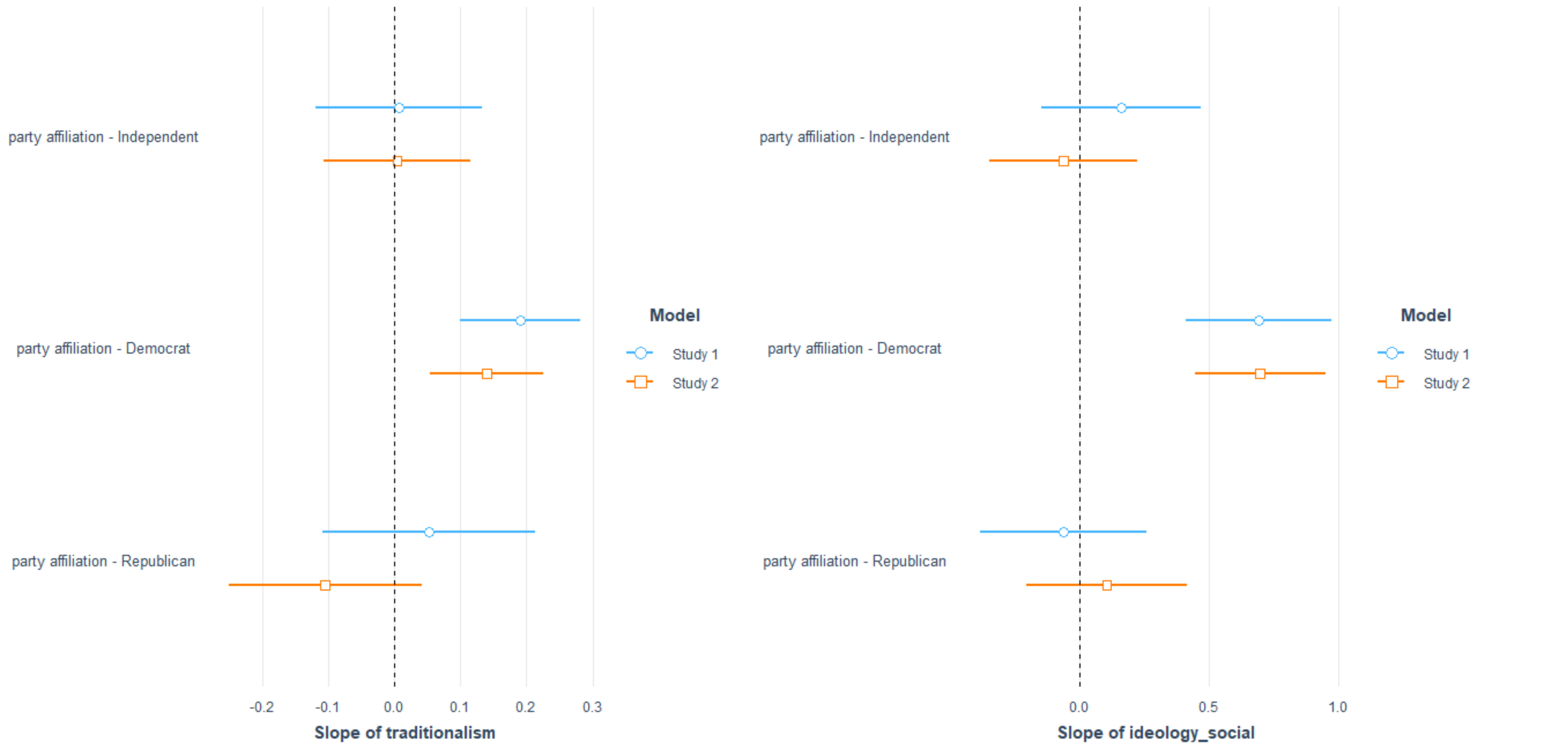

Figure S38. Studies 1 and 2 forest plots showing conditional effects (based on simple slopes analyses) of moderated linear regressions, in which COVID-19 precautionary behaviors was regressed separately on either traditionalism (left pane) or social conservatism (right pane), political party affiliation, and the two-way interaction between party affiliation and the particular ideology variable. Plotted coefficients are unstandardized. Lines indicate 95% confidence intervals.

What drives partisan differences in the relationship between socially conservative political attitudes and COVID-19 precautions?

We then tested whether the combined effects of economic conservatism, the trust in scientists composite, the trust in liberals and moderates composite, and the liberal media consumption composite jointly suppressed the relationship between either traditionalism or social conservatism separately, and the precautionary behaviors composite. First, in Study 1, the combined indirect effect of the four suppressors was negative and significant among Republicans and Independents when using either social conservatism or traditionalism as the independent variable of interest (**social conservatism** - Republicans: bootstrapped standardized indirect effect = -.15, 95% CI [-.22, -.08]; Independents: indirect effect = -.12, 95% CI [-.21, -.04]); **traditionalism** - Republicans: bootstrapped standardized indirect effect = -.10, 95% CI [-.19, -.03]; Independents: indirect effect = -.11, 95% CI [-.19, -.04]), demonstrating suppression. In Study 2, the indirect effect was negative and significant among Independents for both social conservatism and traditionalism (social conservatism: bootstrapped standardized indirect effect = -.27, 95% CI [-.38, -.17]; traditionalism: indirect effect = -.20, 95% CI [-.28, -.12]). Among Republicans, the indirect effect was significant for traditionalism, but only marginal for social conservatism (social conservatism: bootstrapped standardized indirect effect = -.07, 95% CI [-.15, 0]; traditionalism: indirect effect = -.12, 95% CI [-.21, -.04]).

Second, in Study 1, the combined effects of the suppressors rendered the moderation of political party on relationships between precautionary behaviors and either social conservatism or traditionalism no longer significant between Democrats on the one hand, and either Republicans or Independents on the other (Democrat-Republican: βs = -.07 – .03, ps = .232 – .571; Democrat-Independent: βs = -.02 – -.002, ps = .673 – .965). In Study 2, the slope of the relationship between either social conservatism or traditionalism, and precautionary behaviors,

did not significantly differ between Democrats and Independents (βs = -.04 – .04, ps = .326 – .433). However, while there was no significant difference in slopes between Democrats and Republicans when considering traditionalism (β = -.04, p = .452), there was a marginally significant difference for the social conservatism-precautions relationship (β = -.09, p = .079)

Third, in Study 1, simple slopes analyses revealed that, after including the effects of the suppressors and their interactions with political party, there were significant conditional effects between either social conservatism or traditionalism and the precautionary behaviors composite among Democrats (social conservatism: β = 0.27, t(820) = 3.95, p = 8.37e-5; traditionalism: β = 0.15, t(819) = 2.86, p = .004), Republicans(social conservatism: β = 0.15, t(820) = 2.08, p = .038; traditionalism: β = 0.21, t(819) = 2.21, p = .027), and Independents (social conservatism: β = 0.23, t(820) = 3.25, p = .001; traditionalism: β = 0.15, t(819) = 2.05, p = .041). In Study 2, there were conditional effects among Democrats (social conservatism: β = 0.31, t(812) = 5.12, p = 3.91e-7; traditionalism: β = 0.13, t(812) = 2.56, p = .011) and Independents (social conservatism: β = 0.23, t(812) = 3.20, p = .001; traditionalism: β = 0.22, t(812) = 3.09, p = .002), while among Republicans, the conditional effect was significant with social conservatism (β = 0.15, t(812) = 2.23, p = .026), and was non-significant with traditionalism (β = 0.06, t(812) = .65, p = .513).

In sum, while these results are largely consistent with the analyses presented in the main text—suggesting that the identified suppressors are acting on both social conservatism and traditionalism—the above results for Study 2 indicate that among Republicans, the effect on the relationship between traditionalism and precautionary behaviors is weaker compared to their effect on the social conservatism-precautions association.

However, after accounting for the effects of the demographic variables and other covariates described in the Main Text, the relationship between traditionalism and COVID-19 precautions was no longer significant among Democrats in either Studies 1 or 2, among Independents in Study 1, and among Republicans in Study 2. However, conceptually similar to the results using the overall socially conservative attitudes composite and described in the Main Text, the addition of these covariates did not attenuate the relationship between social conservatism and COVID-19 precautions in either study among Democrats and Independents, or in Study 1 among Republicans. These results suggest that the relationship between social conservatism and precautions is more robust to the inclusion of the added covariates compared to the traditionalism-precautions relationships.

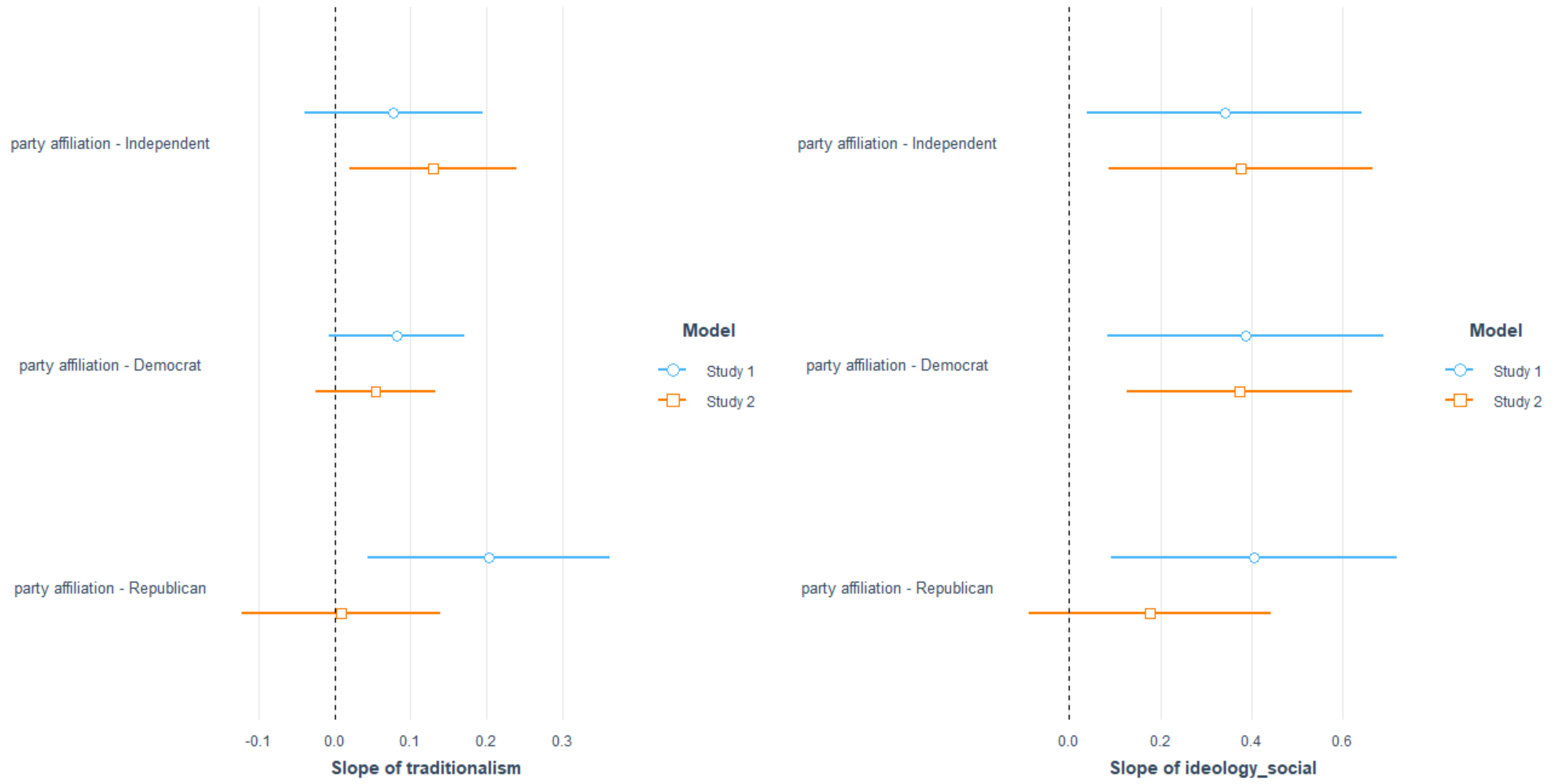

Figure S39. Studies 1 and 2 forest plot showing conditional effects (based on simple slopes analyses) of either traditionalism (left pane) or social conservatism (right pane) on the COVID-19 precautions composite from moderated linear regressions. Additionally, these models include both the pre-identified suppressor variables, as well as a wide variety of covariates. These additional covariates were as follows: age, gender, ethnicity, income, education, pre-existing health conditions, self-reported density of local neighborhood, self-reported estimates of local COVID-19 prevalence, the extent to which one's job required leaving the household, and pathogen disgust sensitivity. Plotted coefficients are unstandardized. Lines indicate 95% confidence intervals.

Disgust sensitivity, politics, and precautionary COVID-19 behaviors

Consistent with the main text analyses, in both studies, pathogen disgust sensitivity correlated with both traditionalism and social conservatism among Democrats, whereas those relationships did not obtain among Republicans. Among Independents, neither traditionalism or social conservatism associated with disgust sensitivity in Study 1, but in Study 2, social conservatism was positively correlated with disgust, while disgust's relationship with traditionalism approached significance. These results are consistent with the analyses presented in the main text.

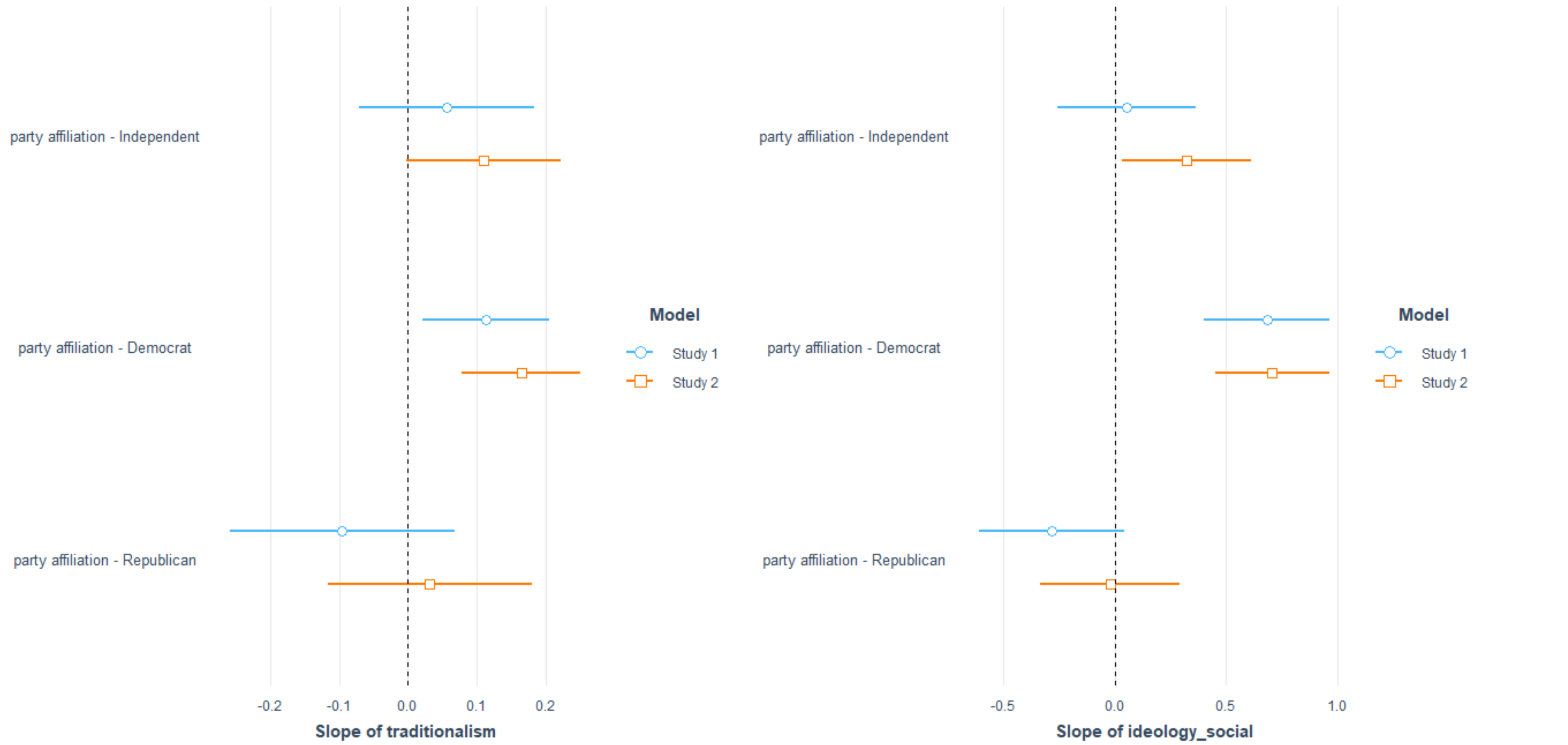

Figure S40. Studies 1 and 2 forest plot showing conditional effects (based on simple slopes analyses) of moderated linear regressions, in which pathogen disgust sensitivity was regressed separately on either traditionalism (left pane) or social conservatism (right pane), political party affiliation, and the two-way interaction between party affiliation and the particular ideology variable. Plotted coefficients are unstandardized. Lines indicate 95% confidence intervals.

8. Effects of perceived closeness to preferred political party affiliation

Mirroring public polling (Pew Research Center, 2020), our data indicate that Republicans and Democrats are responding to the COVID-19 outbreak differently, with Republicans considering the direct health threats posed by the pandemic as less serious relative to Democrats. However, such overarching patterns obscure expectable heterogeneity in the extent to which party affiliation reflects broader aspects of an individual's self-concept and identity. We therefore considered the possibility that perceived closeness to one's preferred political party might relate to COVID-19 precautionary behaviors along party-specific lines. That is, in light of increased skepticism toward the pandemic, the degree of perceived closeness with the Republican party could negatively correlate with precautions, whereas closeness with the Democrat party may positively correlate with prophylaxis. Participants were asked four questions about how closely they identified with their preferred political party affiliation, (e.g., "I identify with other members of the ___ party"). These items were drawn from a social identification scale (Ellemers et al., 1999), and the identity fusion scale (Gómez et al., 2011). We averaged these items into a reliable composite (αs = .89 – .90).

Contrary to the above conjecture, in Study 1, among Republicans, perceived degree of closeness with the Republican party positively correlated with precautions; however, this relationship did not obtain in Study 2. Perceived degree of closeness positively associated with precautions among Independents in both Studies, and among Democrats in Study 2, but not Study 1. Because of the lack of consistent replication between Studies 1 and 2, it would be inappropriate to draw any firm conclusions from these findings.

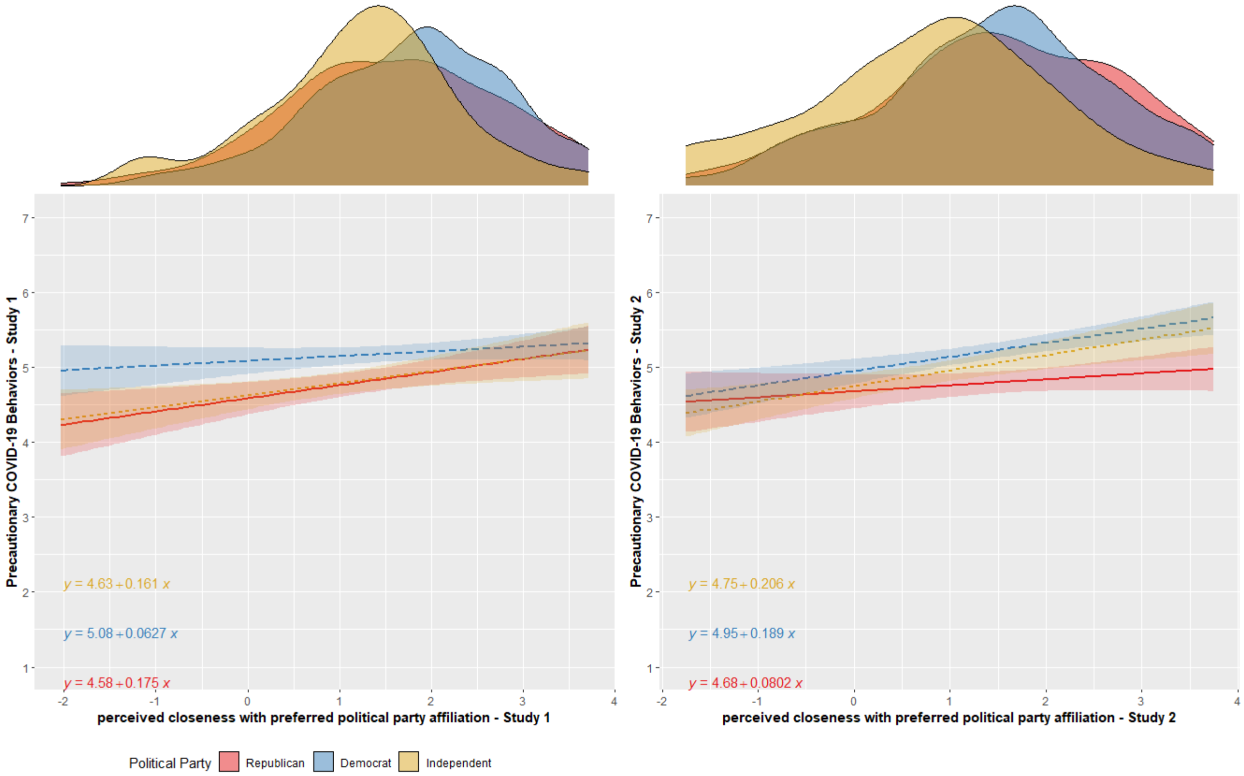

Figure S41. Studies 1 and 2 conditional effects of moderated linear regressions in which precautionary COVID-19 behaviors were regressed on perceived closeness with one's own preferred political party affiliation, political party affiliation, and their two-way interaction. Bands around regression lines are 95% confidence intervals. On each plot, regression equations indicate the conditional simple slopes relationships between COVID-19 precautions and perceived closeness with preferred political party affiliation by political affiliation (the perceived closeness composite measure has been z-scored, hence slopes are standardized).

9. Effects of time on the relationship between political party affiliation and COVID-19 precautions

In the Pilot Study, there was no significant difference between Democrats and Republicans in mean COVID-19 precautions (see S2 Appendix), whereas in Studies 1 and 2, Democrats reported slightly more COVID-19 precautions than did Republicans (see Main Text). As an exploratory analysis, we considered the possibility that, as time progressed, the pandemic became increasingly politicized, potentially resulting in Republicans taking fewer precautions

relative to Democrats. Because the Pilot Study and Studies 1 and 2 were conducted several months apart (April 17th, May 29th, and July 11th, 2020, respectively), we tested whether partisan differences in COVID-19 precautions increased over time across the three studies.

In order to test this possibility, a two-way ANOVA was conducted pooling participants from all three studies that examined the effect of time (three time points, corresponding to each of the three studies) and political party affiliation (only Democrats and Republicans, because Independents were not included in the Pilot Study) on COVID-19 precautions. Note that we used the COVID-19 precautionary behaviors composite from the Pilot Study (see S1 Appendix) in order to compare across all three studies, because some items from the COVID-19 precautionary behaviors composite used in Studies 1 and 2 were not included in the Pilot Study. The main effect of time was not statistically significant ($F[1, 1597] = .05$, $p = .950$), nor was the interaction between time and political party affiliation ($F[1, 1597] = 1.52$, $p = .219$), see Figure S42. These results are not consistent with the hypothesis that increasing politicization resulted in a greater difference in COVID-19 precautions between Democrats and Republicans over the period examined.

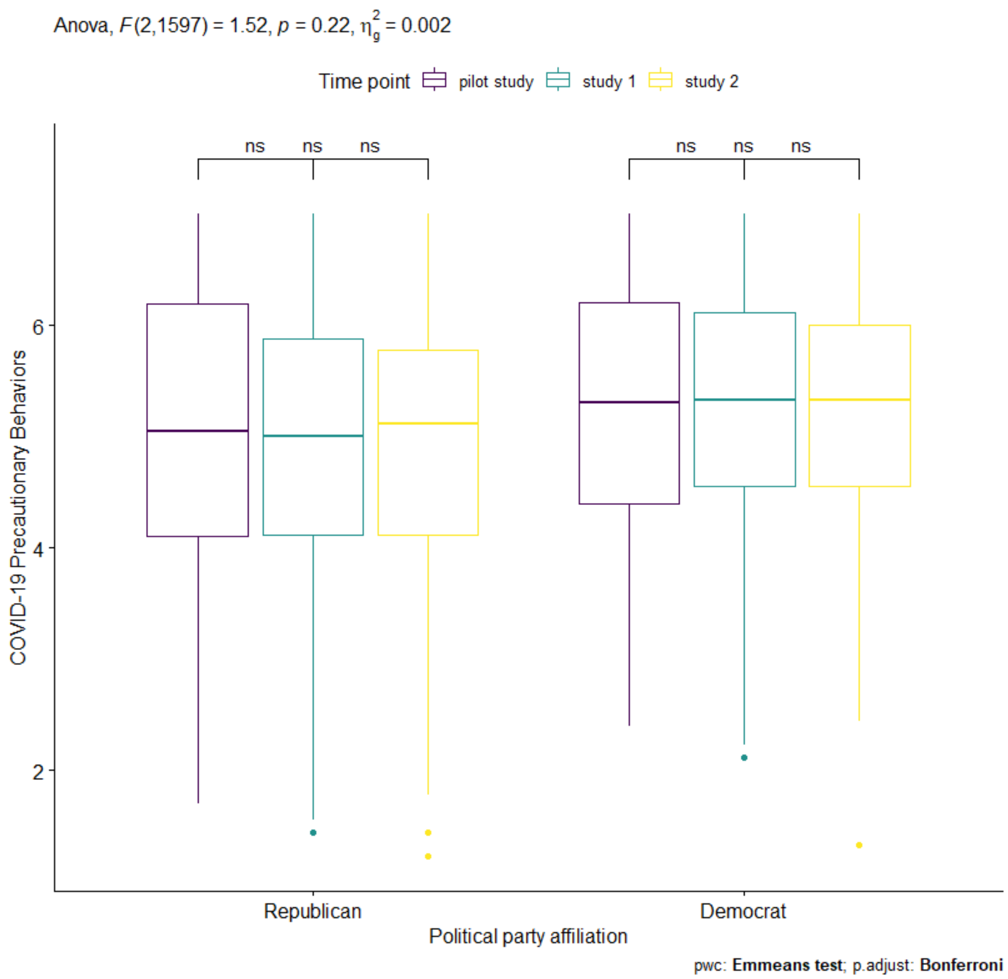

Figure S42. Results of two-way ANOVA examining the effects of time and political party affiliation on COVID-19 precautions. Note that among both Democrats and Republicans, self-reported COVID-19 precautions did not increase across the three time points (spanning from April to July 2020). Pairwise simple effects were obtained using estimated marginal means, and p-values were adjusted using the Bonferroni method.

References

Ellemers, N., Kortekaas, P., & Ouwerkerk, J. (1999). Self-categorisation, commitment to the group and group self-esteem as related but distinct aspects of social identity. *European Journal of Social Psychology - EUR J SOC PSYCHOL*, *29*. https://doi.org/10.1002/(SICI)1099-0992(199903/05)29:2/33.3.CO;2-L

Gómez, A., Brooks, M., Buhrmester, M., Vázquez, A., Jetten, J., & Swann, W. (2011). On the nature of identity fusion: Insights into the construct and a new measure. *Journal of Personality and Social Psychology*, *100*, 918–933. https://doi.org/10.1037/a0022642

Pew Research Center. (2020). *Republicans, Democrats move even further apart in coronavirus concerns*. https://www.pewresearch.org/politics/2020/06/25/republicans-democrats-move-even-further-apart-in-coronavirus-concerns/

Sparks, A. M., Fessler, D. M. T., Chan, K. Q., Ashokkumar, A., & Holbrook, C. (2018). Disgust as a mechanism for decision making under risk: Illuminating sex differences and individual risk-taking correlates of disgust propensity. *Emotion (Washington, D.C.)*, *18*(7), 942–958. https://doi.org/10.1037/emo0000389
