# Supplementary material for: Of pathogens and party lines: Social conservatism positively associates with COVID-19 precautions among U.S. Democrats but not Republicans
Source: PLoS One. 2021 Jun 29;16(6):e0253326. doi: 10.1371/journal.pone.0253326 (PMC8241032; doi:10.1371/journal.pone.0253326)
Supplement: Attachment — Submitted filename: PlusOne_Of_Pathogens_and_Party_Lines_Review_03-31-21 (FINAL).docx [file pone.0253326.s005.docx]

Of Pathogens and Party Lines: Social Conservatism Positively Associates with COVID-19 Precautions among U.S. Democrats but not Republicans

PLOS ONE

The paper reports two correlational M-Turk studies with samples of U.S. American adults and investigates potential ideological differences in COVID-19 threat reactivity. Specifically, the research investigated the relationship between social conservatism and COVID-19 precautionary behavior in light of the hyper-politicized partisan landscape of the United States.

In accordance with a model wherein ideological differences stem from differences in threat reactivity, they found that social conservatives (only among democrats) tended to be more pathogen-avoidant (based on the degrees of COVID-19 precautionary behavior). However, this was not found to be true for either Independents or Republicans. Their approach, both theoretical and empirical, to provide an explanation to why this was not the case for the most conservative partisan group (Republicans), was in my opinion satisfactory and yield a good theoretical and empirical account.

As a whole I believe that when put in more context with the literature on threat-sensitivity and political ideology, which I recommend doing so, this manuscript could also contribute to the question of a *lack* of conservative shift during the COVID-19 crisis in the United States. However, there are some issues that should be addressed before suggesting this manuscript for publication. Issues concerning the general contextualization, the body of literature covered, and more crucially, the reporting of the study’s results should be significantly addressed before recommending this manuscript for publication. **In what follows I include some areas to consider for improvement.**

-In general, I felt that the introduction to the manuscript (p. 3, line 49) could benefit from taking a step back from the theory and the data-driven points. It would be good, before going into the theory, to provide a general description of the political state of affairs and dynamics in the U.S. with relation to the COVID-19 pandemic at the time of the project/data collection. Following from the general rationale of the first sentence of the introduction, I feel that a more detailed description of the situation (the hyper-politization of the pandemic) would facilitate a better segue from the real-world problem to the theoretical research question. Perhaps a couple more sentences (or maybe an additional paragraph) that will situate the reader in the very real and specific time and moment of the COVID-19 Pandemic and the general political landscape (Election Year in the U.S.), will provide further justification to the timely question this manuscript addresses.

-On another but related point, the question of conservative shift (e.g., Bonanno & Jost, 2006; Beall, Hofer, & Schaller, 2016) under real-life common threats in the case of COVID-19 seems to be one important question that the paper does not addresses directly, but that would be important to do so. Maybe addressing this at the beginning, or at some point in the introduction would also put the results and findings in conversation with this general theoretical premise from a threat-sensitivity (or system-threat) perspective. Based on the threat literature and political ideology, some might have predicted potential political ideological shifts (a conservative one) based on exposure to the real life (and the multiple) threats that COVID poses. It seems like they find this pattern to be present only among Democrats (which is interesting in its own way), and that other political factors suppressed this in the case of Republicans.

I wonder if contextualizing the findings with respect to the suppressor variables (e.g., lower trust in scientists, liberal and moderate sources, lesser consumption of liberal news media, and greater economic conservatism) could further help clarify why, at least in this case, a general move from social conservatism toward increases in protective behaviors against COVID-19 was not observed. The incorporation of the question of ideological shifts, as well as the real-life implication of the impact of COVID-19 on the 2020 elections (either in the introduction or general discussion), will strengthen the theoretical contribution of this manuscript.

-(p. 12, line 264) When describing the data collection periods, the specific dates for the data collection period should be included when describing the pilot study as well. It may be useful as well, based on my impression of the supplementary materials, to include time effect differences on the correlations of social conservatism and COVID-19 precautionary behavior, given that they found no significant difference between Republicans and Democrats in their initial pilot study:

“We find no significant baseline differences in mean precautionary behaviors between Republicans (M = 5.05, SD = 1.31) and Democrats (M = 5.20, SD = 1.17) in our sample (t[215.68] = -1.08, *p* = .282).” (p. 9, supplement).

It could very well be, that Time might be playing a role with regards the strength and significance of this relationship. Therefore, it might be appropriate to examine if this positive association keeps fading away with time, as the polarization and politization of COVID-19 precautionary mandates in the United States kept increasing with time. This could go in a footnote, or even in one of the supplements.

- (pp. 19-25) Perhaps my major concern about the current state of the manuscript has to do with the general Results section. I recommend substantial rewriting of this section. As it stands right now, the present iteration of the first section subtitled “***Do socially conservative political attitudes predict precautionary behavior?”*** seems vague and does not follow a strong line of organization. For instance, the first paragraph claims that “*using linear regression with moderation, in both studies, COVID-19 prophylaxis associated with socially conservative political attitudes among Democrats*” (p. 19, line 431)” but they do not provide any statistics to support this claim in the main text. I feel that the authors did a good job of reporting this results in their supplement, but they seem to have excluded important statistical information in the main text. I suggest integrating certain components from their supplementary material into the main text, because there is just too little information provided in the result section of the main text. Furthermore, with regards to this specific section, the description of the figure should not be in the text body, but rather as a *note* for the figure. I am not sure if they intended to include this information here, of if it was meant to be part of the description of the figure.

- For instance, I recommend that the part on p. 20 (lines 400-451) be used as the note for the figure, and the rest about simple slope analyses should be integrated into the main text. The same is true for the results reported on p. 22 (line 480). Because these all seems issues of style and formatting, I am confident that the authors will be able to address these concerns.

In the supplement the authors demonstrate the step-by-step analytical process to determine the general suppressors in a very satisfactory way. I would recommend they also integrate some of this detailed analysis in the main text, because it seems very important (p. 9, supplement).

Finally, I would also recommend including the analyses of conditional effects of moderated linear regressions in which COVID-19 precautions were separately regressed on each individual (centered) suppressor variable for Study 1 and Study 2 (p. 15, supplement). This analysis really makes the argument and findings clearer and more telling. In summary, then, I believe that a general reworking of the Results section is advisable prior to publication.

MINOR CONCERNS:

-(p. 5, line 110) The authors wrote:

“Thus, prior research on the traditional-norms account has not sufficiently taken into account the cost-benefit trade-offs of many threat and pathogen avoidance behaviors, in turn limiting the ecological validity of the findings.”

This is a turning point in the introduction that leads to one of the central premises of their hypotheses, and it highlights the magnitude of the present contribution. As it stands right now, the text seems a bit vague and overly general. To enhance the strength of their argument, I would recommend the authors directly cite the work they are alluding to here and incorporate any other studies that have already tried to address those limitations (successfully or not). I feel the paper would benefit from being as direct and concise as possible with regards to the work they are citing, constructively criticizing, and building upon.

-(p. 6, line 128-138) The authors wrote:

“Some of the precautions recommended or required by public health authorities interfere with engaging in traditional practices (e.g., social distancing precludes family gatherings, public sporting events, in-person religious services, etc.). Accordingly, two possibilities exist regarding the relationship between threat sensitivity, socially conservative attitudes, and COVID-19 precautions. On the one hand, if more threat-sensitive individuals focus on the precautions themselves, construing these as infringing on traditions, then they will report lower precautionary behavior than less threat-sensitive individuals–and, indeed, may view such behaviors as threats in themselves, endangering individual liberties. On the other hand, if more threat-sensitive individuals focus on the danger posed by COVID-19 more so than the conflict with various traditions entailed by precautionary behaviors, then they will report both greater precautionary behavior and greater valuation of traditions than will less threat-sensitive individuals.”

I really like this conceptualization, and it helps facilitate a more holistic understanding of the dynamics of social conservatism and threat-avoidance in a more complex way than one normally encounters (which is a simple linear, “cause and effect” way). One recommendation, however, would be to elaborate on the explanation of the first possibility, because it is more novel in political psychology. I recommend describing the second possibility first (because it is the simpler one), and then discussing the one about “focusing on the precautions themselves.” I recommend turning the 4-line sentence, into perhaps two shorter sentences for the purpose of clarity.

-(p. 11, line 241) typo: “In” after last sentence of section.

-(p. 14, line 301) All the measures included here should be presented in the precise order that they were presented to participants in the study. Also, is the order of measures consistent across studies?

-(p. 14, line 320) I would recommend elaborating further on the development of the *social conservative attitude* scale, because it is the central variable of interest and the operationalization deviates somewhat from the more “traditional” or “common” conception and usage of ideology (the left-right ideological self-placement item). I suggest that the authors devote more space explaining and providing concrete examples about the development and operationalization of this variable in the text of the manuscript.

-(p. 16, line 358) It would be useful if there were sample items provided, rather than a description of the contents of these items.

-(p. 16, line 360) I would recommend using sub-headers for each of the 6 categories, to make the last section easier to follow. As it reads now, this passage may lead to a bit of confusion. For example, the authors write:

(p. 17, line 382) The authors wrote:

“Third, participants were asked about the extent to which they were preparing for an economic downturn, such as by delaying major financial decisions. We averaged these behavior items into an economic precautions composite, which was reliable (αs = .75 – .78).”

I will recommend doing something like this:

3. Economic precautions: Participants were asked about the extent to which they were preparing for an economic downturn by delaying major financial decisions. We averaged these behavioral items into a composite scale, which was reliable (αs = .75 – .78).

-(p. 19, line 416) It would be useful to remind the reader of the total sample size and report the precise number of self-identified “Other” parties.

-(p. 19, line 423 and throughout the results section) Many of the reported statistics (e.g., *p* = 3.33e-6) do not conform to APA style.

-(pp. 20, line 452) They report:

“Finally, in both studies, the interactions between political party affiliation and socially conservative attitudes were significant between Democrats on the one hand, and both Republicans (Study 1: B = -0.80, SE = .29, t(857) = -2.77, p = .006; Study 2: B = -0.79, SE = .26, t(854) = -3.04, p = .002) and Independents (Study 1: B = -0.69, SE = .25,  t(857) = -2.72, p = .007; Study 2: B = -0.78, SE = .23, t(854) = -3.39, p = .001) on the other. Slopes did not significantly differ between Independents and Republicans (Study 1: B = 0.11, SE = .31, t(857) = .35, p = .728; Study 2: B = 0.01, SE = .28, t(854) = .04, p = .969).”

It is not clear what are they trying to say with this. It seems like the only thing they should report here is that the slopes between Independents and Republicans did not differ from each other. The same is true on p. 22 (line 499). I recommend clarifying these points.

-(p. 24, line 547) The authors reported combined statistics like this:

“We then performed simple slopes analyses, finding that, sensibly, in both studies, disgust sensitivity associated with precautionary behaviors among Democrats (Bs = .20 – .24, ps = 7.60e-7 – 2.66e-5), Republicans (Bs = .23 – .36, ps = 2.40e-8 – 4.76e-4), and Independents (Bs = .27 – .40, ps = 5.63e-11 – 1.16e-5).”

I would recommend reporting these statistics separately for each study in a consistent way throughout the Results section.

-(p. 29, line 648-651) They wrote:

“[…] Nevertheless, in both studies, we found significant evidence that suppression by the target variables occurred, such that, when accounting for the suppressors, political party no longer moderated the relationship between socially conservative attitudes and precautions.”

The last sentence in the first limitations paragraph seems a bit contradictory to the main points they are raising just before. I recommend to simply take it out.

-(p. 29, line 652) They wrote:

“Second, because of the cross-sectional, correlational, non-experimental design of the research, it is impossible to draw definitive conclusions regarding causal relationships between the phenomena of interest.”

This statement seems pretty vague, and it would be beneficial to suggest ways of overcoming this limitation. Perhaps elaborate a bit more on the specific causal relationship you would like to address and report ways that one could potentially do so with different types of data or research designs.

***Concluding remarks:***

In sum, the inconsistencies with regards to the organization and content of the results section made the paper somewhat difficult to follow and comprehend. It was difficult to get a clear picture from the Results section in the main text alone, and without the supplemental material it was difficult to get a full picture of the results, as well as the analytical strategies implemented. I strongly recommend that these issues are resolved prior to publication.
